# Dynamic Complex‐to‐Complex Transformations of Heterobimetallic Systems Influence the Cage Structure or Spin State of Iron(II) Ions

**DOI:** 10.1002/anie.201914629

**Published:** 2020-01-09

**Authors:** Matthias Hardy, Niklas Struch, Julian J. Holstein, Gregor Schnakenburg, Norbert Wagner, Marianne Engeser, Johannes Beck, Guido H. Clever, Arne Lützen

**Affiliations:** ^1^ Rheinische Friedrich-Wilhelms-Universität Bonn Kekulé-Institut für Organische Chemie und Biochemie Gerhard-Domagk-Straße 1 53121 Bonn Germany; ^2^ Technische Universität Dortmund Fakultät für Chemie und Chemische Biologie Otto-Hahn-Straße 6 44227 Dortmund Germany; ^3^ Institut für Anorganische Chemie Rheinische Friedrich-Wilhelms-Universität Bonn Gerhard-Domagk-Straße 1 53121 Bonn Germany; ^4^ Current address: Arlanxeo Netherlands B.V. Urmonderbaan 24 6167 RD Geleen The Netherlands

**Keywords:** iron complexes, palladium complexes, self-assembly, subcomponent self-assembly, supramolecular Chemistry

## Abstract

Two new heterobimetallic cages, a trigonal‐bipyramidal and a cubic one, were assembled from the same mononuclear metalloligand by adopting the molecular library approach, using iron(II) and palladium(II) building blocks. The ligand system was designed to readily assemble through subcomponent self‐assembly. It allowed the introduction of steric strain at the iron(II) centres, which stabilizes its paramagnetic high‐spin state. This steric strain was utilized to drive dynamic complex‐to‐complex transformations with both the metalloligand and heterobimetallic cages. Addition of sterically less crowded subcomponents as a chemical stimulus transformed all complexes to their previously reported low‐spin analogues. The metalloligand and bipyramid incorporated the new building block more readily than the cubic cage, probably because the geometric structure of the sterically crowded metalloligand favours the cube formation. Furthermore it was possible to provoke structural transformations upon addition of more favourable chelating ligands, converting the cubic structures into bipyramidal ones.

## Graphical abstract

**Lively exchanges**: Two new heterobimetallic cages with iron(II) cations in a sterically strained coordination sphere undergo complex‐to‐complex transformation. Subcomponent‐exchange reactions with sterically less crowded building blocks changed the spin state from high‐ to low‐spin iron(II), whereas ligand exchange with chelating ligands affected the structural transformation from cubic to bipyramidal cages.

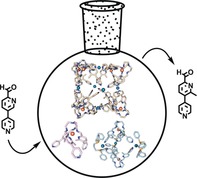

Metallosupramolecular chemistry^1^ has produced many beautiful structures with defined shapes^2^ and fascinating functionality.^3^ Within metallosupramolecular chemistry, a powerful tool is the subcomponent self‐assembly approach, in which aggregates emerge from the assembly of two ligand subunits that form feasible ligands and their complexes in situ. These are formed via the reversible formation of covalent bonds.^4^ This approach was used to prepare numerous structures adopting various properties and features,^5^ for example, host–guest complexes ^[6]^ and switchable systems.6c, 7 The tuning of complex properties is often facilitated by varying one distinct ligand subunit and exchanging one building block.6b, 8 In combination with the reversible character of the covalent bonds, these properties can also allow for building block exchanges in situ, resulting in complex‐to‐complex transformations.7a, 9 Besides subcomponent exchanges, also ligand exchanges,^10^ solvent dependencies,^11^ and light irradiation^12^ were successfully used to perform complex‐to‐complex transformations.

Aside from indisputably beautiful homometallic structures, the subcomponent self‐assembly approach also proved useful for the formation of heterobimetallic cages within the context of the complex‐as‐a‐ligand strategy.^13^ Thus, it is possible to transfer all advantages of the subcomponent self‐assembly approach to systems consisting of more than one type of metal cation. However, to date only a few examples of heterobimetallic complexes have been reported that were built up from subcomponents.13d, 13e, 14

Previously, Drago et al. introduced a ligand system that can be prepared from the commercially available building blocks tris(2‐aminoethyl)amine (TREN), 2‐formylpyridine, 2‐formyl‐6‐methylpyridine, and iron(II) salts.^15^ 2‐Formyl‐6‐methylpyridine‐containing complexes show a stabilization of the high‐spin state of iron(II) cations at room temperature due to steric strain between the methyl groups and nearby pyridine rings, as well as spin‐crossover behaviour in the solid state.^15^, ^16^ These aldehydes were also used to develop 3D architectures with different shapes and sizes that show complex‐to‐complex transformations upon replacement of 2‐formyl‐6‐methylpyridine with less bulky 2‐formylpyridine. These transformations resulted in a change of the spin state of iron(II) cations from high spin to low spin, due to shortening of the Fe−N bonds.9b, 17 The decrease of steric strain might be responsible for the preferential incorporation of 2‐formylpyridine over 2‐formyl‐6‐methylpyridine.9b, 17

In this work we investigated the dynamic behaviour of heterobimetallic structures that assemble through subcomponent self‐assembly. These complexes contain sterically strained iron(II) centres in the paramagnetic high‐spin state. We adopted previously reported subcomponent exchanges and expanded these principles to heterometallic systems. Chemical stimuli transferred the heterobimetallic paramagnetic complexes to their diamagnetic analogues upon reduction of steric strain. In addition, we could perform ligand‐exchange reactions, resulting in the structural transformation of cubic to bipyramidal cages.

Ditopic building block **1** was selected to achieve the formation of *C*_3_‐symmetric iron(II) tris(pyridylimine) moieties^18^ as well as *C*_2_‐ and *C*_4_‐symmetric palladium(II) pyridine moieties. Reaction of 3 equivalents of aldehyde **1** with 1 equivalent of TREN **2** and 1 equivalent of iron(II) tetrafluoroborate hexahydrate in acetonitrile yielded the mononuclear complex **ML‐1**(BF_4_)_2_ as a bright red solid (Scheme 1). This mononuclear complex was characterised by ^1^H NMR and UV/Vis spectroscopy, ESI‐MS spectrometry, vibrating sample magnetometry, and single‐crystal X‐ray diffraction (see the Supporting Information). Mixing 1 equivalent of **ML‐1**(BF_4_)_2_ with 1.5 equivalents of 1,3‐bis(diphenylphosphino)propane palladium(II) triflate ([(dppp)Pd(OTf)_2_]) or 0.75 equivalents of tetrakis(acetonitrile) palladium(II) tetrafluoroborate ([Pd(CH_3_CN)_4_](BF_4_)_2_) in acetonitrile resulted in the formation of heterometallic pentanuclear bipyramidal assembly **BP‐1**(OTf)_6_(BF_4_)_4_ or the tetradecanuclear cubic assembly **CU‐1**(BF_4_)_28_, respectively, each as a bright red microcrystalline solid in high yields (Scheme 1).

**Scheme 1:**
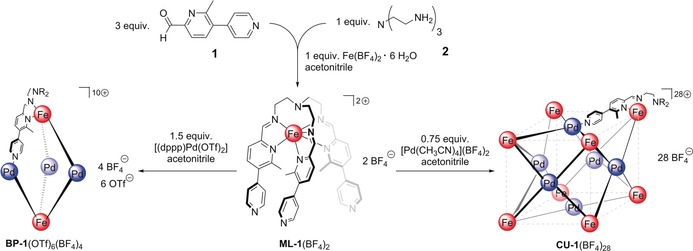
Stepwise self‐assembly of **BP‐1**(OTf)_6_(BF_4_)_4_ and **CU‐1**(BF_4_)_28_ via formation of **ML‐1**(BF_4_)_2_ from **1** and **2**.

The identity and purity of bipyramid **BP‐1**(OTf)_6_(BF_4_)_4_ were proven by ^1^H NMR and UV/Vis spectroscopy and ESI‐MS (see the Supporting Information). When vibrating sample magnetometry and the Evans’ method^19^ were employed, a magnetic susceptibility of *Χ*_m_ *T*=6.0 cm^3^ K mol^−1^ was revealed, which is in very good agreement with the expected value for two uncoupled iron(II) cations in the high‐spin state (6.001 cm^3^ K mol^−1^).^20^ Slow diffusion of diethyl ether into a concentrated solution of **BP‐1**(OTf)_6_(BF_4_)_4_ in acetonitrile gave X‐ray diffraction quality single crystals that could be used to determine its crystal structure (Figure 1). The mixed anionic cage **BP‐1**(OTf)_6_(BF_4_)_4_ crystallises in the monoclinic space group *C*2/*c*, with both homochiral enantiomers [(Δ,Δ) and (Λ,Λ)] present in the unit cell as a racemic mixture and is best described as a trigonal bipyramid or helicate^21^ (for more details see the Supporting Information).

**Figure 1:**
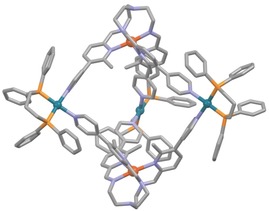
Structure of bipyramidal complex **BP‐1**(OTf)_6_(BF_4_)_4_ as determined by single‐crystal X‐ray diffraction. Hydrogen atoms, counter ions and solvent molecules are omitted for clarity; colour code: grey C, blue N, yellow‐orange P, dark orange Fe, turquoise Pd.

The iron cations are coordinated in an octahedral ligand sphere with an average Fe−N bond length of 2.179 Å, consistent with a paramagnetic high‐spin iron(II) complex.^22^ The palladium centres form the equatorial plane of the complex and are settled in a square‐planar ligand sphere, each coordinated by a bidentate dppp ligand and two 4‐pyridyl moieties.

The identity of the heterobimetallic cube **CU‐1**(BF_4_)_28_ could also be proven by ^1^H NMR and UV/Vis spectroscopy and ESI‐MS (see the Supporting Information). An Evans’ experiment revealed a magnetic susceptibility of *X*_m_ *T*=24.1 cm^3^ K mol^−1^, proving its fully paramagnetic character.^20^ Interestingly, the self‐assembly of **CU‐1**(BF_4_)_28_ was completed after only 16 hours at 50 °C, whereas the self‐assembly of a previously reported diamagnetic analogue13e of this heterobimetallic cube required 120 hours under the same conditions. We attribute this tremendously shorter reaction time of **CU‐1**(BF_4_)_28_ to the larger opening angle of **ML‐1**(BF_4_)_2_, compared to the metalloligand without the additional methyl groups on the pyridine binding to the iron(II) ion. Due to the steric strain around the iron centre, the opening angle becomes larger, and therefore, should be better preorganized to form the heterobimetallic cube.

Very slow evaporation of solvent from an acetonitrile solution of **CU‐1**(BF_4_)_28_ over 3 months yielded single crystals, which diffracted up to 1.2 Å using synchrotron radiation^23^ and allowed for unambiguous structure determination (Figure 2).

**Figure 2:**
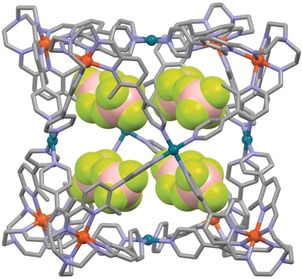
Structure of cube **CU‐1** as determined by single‐crystal X‐ray diffraction using synchrotron radiation. Eight tetrafluoroborate anions occupy the inner void. Hydrogen atoms, solvent molecules, and counter ions outside the cavity are omitted for clarity; colour code: grey C, blue N, light green F, brown‐beig B, dark orange Fe, turquoise Pd.

**CU‐1**(BF_4_)_28_ crystallises in the cubic space group *Fm*−$\bar{3}$ *c* with both homochiral enantiomers [all (Δ) and all (Λ)] present in the unit cell as a racemic mixture. Only one‐third of one ligand and one iron ion is found in the asymmetric unit due to the threefold symmetry axis. All three Fe−N bonds are crystallographically equivalent and show a bond length of 2.105(11) Å, which is consistent with a high‐spin iron(II) complex. The tetravalent palladium ions with their four coordinated pyridine moieties are located on the fourfold symmetry axes of the cubic cage. Eight tetrafluoroborate anions occupy the void of the cage, adapting the cubic arrangement themselves.

The steric strain in **ML‐1**, **BP‐1**, and **CU‐1** results in an elongation of the Fe−N bond lengths and the stabilization of the paramagnetic high‐spin state.9b, 15, 17 Still the situation is energetically unfavourable, and hence, can be exploited in thermodynamically driven complex‐to‐complex transformations during which the steric strain is reduced. The transformation reactions were performed by the addition of a twofold excess of sterically less crowded 2‐formyl‐5‐(4′‐pyridyl)pyridine **3** (per exchangeable methyl‐substituted aldehyde component) to the complex solutions and were monitored by ^1^H NMR spectroscopy. In such a scenario a quantitative incorporation of **3** would lead to a 50:50 or 1:1 ratio of both free aldehydes **1** and **3**. Please note that we had to analyse the signals of the individual aldehyde subcomponents as probes here as integration of ^1^H NMR signals of paramagnetic species is generally not possible, when mixed species with different magnetic properties are present.

Following this approach, addition of 6 equivalents of **3** to a solution of **ML‐1**(BF_4_)_2_ released free 3 equivalents of subcomponent **1** when **3** was incorporated almost quantitatively into the complex, resulting in the formation of previously reported diamagnetic metalloligand **ML‐2**(BF_4_)_2_ in high yield (Scheme 2). Progress of this reaction was indicated by a change in the colour of the solution from bright red to dark purple. After 16 hours at 40 °C the mixture reached equilibrium with free subcomponents **3** and **1** in a 56:44 ratio and a new set of signals in the ^1^H NMR spectrum referring to **ML‐2**(BF_4_)_2_ (see the Supporting Information).

**Scheme 2:**
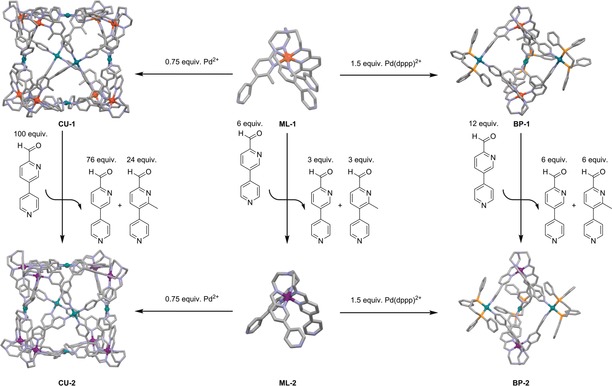
Sterically driven complex‐to‐complex transformations. Red atoms mark iron(II) in the high‐spin state, purple atoms mark iron(II) in the low‐spin state.

The same chemical stimulus can be used to transfer paramagnetic **BP‐1**(OTf)_6_(BF_4_)_4_ quantitatively into the corresponding diamagnetic cage **BP‐2**(OTf)_6_(BF_4_)_4_. After 16 hours at 40 °C the equilibrium ratio of **3** and **1** was determined to be 1:1 and ^1^H NMR signals arose that can be assigned to **BP‐2**(OTf)_6_(BF_4_)_4_ (see the Supporting Information). Also the formation of **BP‐2**(OTf)_6_(BF_4_)_4_ could be shown by UV/Vis spectroscopy (see the Supporting Information). This transformation impressively demonstrates the strong driving force of this subcomponent exchange and the highly dynamic behaviour of this heterobimetallic system. In the course of this exchange reaction, one covalent imine bond, two coordinative Fe−N bonds, and one coordinative Pd−N bond have to be broken, the new building block has to align and to reform all formerly cleaved bonds again for each exchanged subcomponent.

Similar treatment of cubic cage **CU‐1**(BF_4_)_28_ with a greater excess of 100 equivalents of **3** resulted in the transformation to the analogous diamagnetic cube **CU‐2**(BF_4_)_28_. Unlike with **ML‐1**(BF_4_)_2_ and **BP‐1**(OTf)_6_(BF_4_)_4_, however, the subcomponent exchange required considerably more time. After three days at 65 °C an equilibrium ratio between **1** and **3** of 30:70 was determined, which is close to the expected 24:76 ratio. However, the use of only 48 equivalents of **3** did not lead to a complete transformation (see the Supporting Information). Therefore, these experiments show that incorporation of **3** into the cubic assembly is slightly more favoured than incorporation of **1**, but less pronounced than in the case of the bipyramidal assemblies.

Hence, in case of the cubic cage the sterically stressed complex probably is not as disfavoured as for **ML‐1**(BF_4_)_2_ and **BP‐1**(OTf)_6_(BF_4_)_4_. The larger opening angle of **ML‐1**(BF_4_)_2_ caused by the additional methyl groups seems to be a better preorganization for the assembly of cubic structures than the smaller opening angle in **ML‐2**(BF_4_)_2_. This assumption is corroborated by the much faster formation of **CU‐1**(BF_4_)_28_ compared to that of **CU‐2**(BF_4_)_28_, although it is difficult to dissect whether this is due to the kinetics or the thermodynamics of the assembly process.

Besides sterically driven subcomponent‐exchange reactions that influence the spin state of iron(II) cations, the system also allows for structural conversions upon chemical stimulus, maintaining the initial spin state of the complexes (see the Supporting Information). The addition of dppp as a chelating ligand to cubic cages **CU‐1** and **CU‐2** leads to complex‐to‐complex transformations, yielding the bipyramidal complexes **BP‐1** and **BP‐2** and excess metalloligands **ML‐1** and **ML‐2**, respectively (Scheme 3, Supporting Information). In these reactions two 4‐pyridyl donors at palladium(II) cations are replaced by one bidentate dppp ligand, leading to major structural changes. Since the contingent of iron(II) cations in the cubic assemblies is greater than in the bipyramidal cages, excess metalloligand is produced as a side product in these transformations. Addition of 6 equivalents of dppp transforms one cubic cage into two bipyramidal assemblies and four free metalloligands, increasing the overall entropy of the system, which might be an additional driving force for this reaction besides the enthalpically strongly preferred coordination of the dppp ligand to palladium(II) cations to facilitate these conversions.^24^

**Scheme 3:**
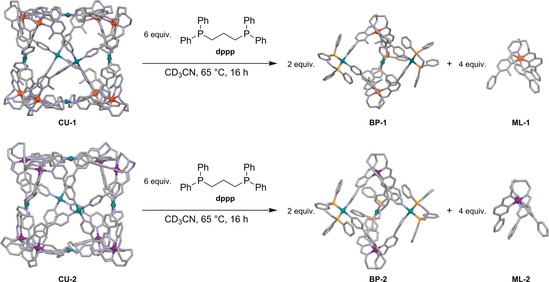
Structural transformations of cubic cages into bipyramidal complexes, producing the metalloligands as side products.

In conclusion, we presented the stepwise assembly of two heterobimetallic cages **BP‐1** and **CU‐1** from the same *C*_3_‐symmetric metalloligand **ML‐1** adopting the well‐implemented molecular library approach. Compared to previously reported diamagnetic complexes **ML‐2**, **BP‐2**, and **CU‐2** 13e the simple addition of a methyl group in 6‐position of the pyridyl ring leads to a substantial amount of steric strain and changes the spin state of the iron(II) cations from diamagnetic low spin to paramagnetic high spin. This impressively shows how rather small changes of the ligand system can cause tremendous changes of the complex properties.

Paramagnetic complexes **ML‐1** and **BP‐1** showed a very high tendency to undergo subcomponent‐exchange reactions, resulting in the first reported complex‐to‐complex transformations of heterobimetallic complexes to heterobimetallic complexes in which metal‐bridging components are exchanged. In this case, 2‐formyl‐6‐methyl‐5‐(4′‐pyridyl)‐pyridine **1** could be replaced by sterically less crowded 2‐formyl‐5‐(4′‐pyridyl)pyridine **3**, transforming the high‐spin complexes into their low‐spin analogues.13e The cubic cage **CU‐1**, however, was found to exchange **1** much less readily, probably because the opening angle of the sterically crowded metalloligand makes it kinetically and thermodynamically favourable to assemble the cubic structure.

Addition of bidentate dppp to cubic assemblies **CU‐1** and **CU‐2** led to a ligand‐exchange reaction, in which two 4‐pyridyl donors around palladium(II) cations were replaced by the chelating ligand. These exchanges resulted in complex‐to‐complex transformations to the bipyramidal cages **BP‐1** and **BP‐2**, respectively, maintaining the spin state of iron(II). Since the iron(II) to palladium(II) ratio decreases on going from the cubic to the bipyramidal cages, the corresponding free metalloligands **ML‐1** and **ML‐2** are formed as side products in these transformations. The presented complex‐to‐complex transformations beautifully show the highly dynamic behaviour of the heterobimetallic systems, allowing the change of magnetic or structural properties upon certain chemical stimuli.

## Conflict of interest

The authors declare no conflict of interest.

## Supporting information

As a service to our authors and readers, this journal provides supporting information supplied by the authors. Such materials are peer reviewed and may be re‐organized for online delivery, but are not copy‐edited or typeset. Technical support issues arising from supporting information (other than missing files) should be addressed to the authors.

Supplementary

## References

[1] *For some reviews, see*:

[1a] R. Chakrabarty , P. S. Mukherjee , P. J. Stang , Chem. Rev. 2011, 111, 6810–6918; 21863792 10.1021/cr200077m PMC3212633

[1b] M. M. J. Smulders , I. A. Riddell , C. Browne , J. R. Nitschke , Chem. Soc. Rev. 2013, 42, 1728–1754; 23032789 10.1039/c2cs35254k

[1c] K. Harris , D. Fujita , M. Fujita , Chem. Commun. 2013, 49, 6703–6712; 10.1039/c3cc43191f 23783176

[1d] L. Chen , Q. Chen , M. Wu , F. Jiang , M. Hong , Acc. Chem. Res. 2015, 48, 201–210; 25517043 10.1021/ar5003076

[1e] C. J. Brown , F. D. Toste , R. G. Bergman , K. N. Raymond , Chem. Rev. 2015, 115, 3012–3035; 25898212 10.1021/cr4001226

[1f] T. R. Cook , P. J. Stang , Chem. Rev. 2015, 115, 7001–7045. 25813093 10.1021/cr5005666

[2a] M. Fujita , M. Tominaga , A. Hori , B. Therrien , Acc. Chem. Res. 2005, 38, 369–378; 15835883 10.1021/ar040153h

[2b] K. Suzuki , M. Tominaga , M. Kawano , M. Fujita , Chem. Commun. 2009, 1638–1640; 10.1039/b822311d 19294246

[2c] T. R. Cook , Y.-R. Zheng , P. J. Stang , Chem. Rev. 2013, 113, 734–777; 23121121 10.1021/cr3002824 PMC3764682

[2d] L.-J. Chen , H.-B. Yang , M. Shinoya , Chem. Soc. Rev. 2017, 46, 2555–2576; 28452389 10.1039/c7cs00173h

[2e] S. Mukherjee , P. S. Mukherjee , Chem. Commun. 2014, 50, 2239–2248; 10.1039/c3cc49192g 24468830

[2f] M. Han , D. M. Engelhard , G. H. Clever , Chem. Soc. Rev. 2014, 43, 1848–1860. 24504200 10.1039/c3cs60473j

[3a] D. Fielder , D. H. Leung , R. G. Bergman , K. N. Raymond , Acc. Chem. Res. 2005, 38, 349–358; 15835881 10.1021/ar040152p

[3b] M. J. Hannon , Chem. Soc. Rev. 2007, 36, 280–295; 17264930 10.1039/b606046n

[3c] H. Amouri , C. Desmarets , A. Bettoschi , M. N. Rager , K. Boubekeur , P. Rabu , M. Drillon , Chem. Eur. J. 2007, 13, 5401–5407; 17361971 10.1002/chem.200700265

[3d] M. Yoshizawa , J. K. Klosterman , M. Fujita , Angew. Chem. Int. Ed. 2009, 48, 3418–3438; 10.1002/anie.200805340 19391140

[3e] T. R. Cook , V. Vajpayee , M. H. Lee , P. J. Stang , K.-W. Chi , Acc. Chem. Res. 2013, 46, 2464–2474; 23786636 10.1021/ar400010v PMC3833955

[3f] M. L. Saha , S. Neogi , M. Schmittel , Dalton Trans. 2014, 43, 3815–3834; 24469101 10.1039/c3dt53570c

[3g] X. Yan , T. R. Cook , P. Wang , F. Huang , P. J. Stang , Nat. Chem. 2015, 7, 342–348; 25803473 10.1038/nchem.2201

[3h] S. Zarra , D. M. Wood , D. A. Roberts , J. R. Nitschke , Chem. Soc. Rev. 2015, 44, 419–432; 25029235 10.1039/c4cs00165f

[3i] S. H. A. M. Leenders , R. Gramaga-Doria , B. de Bruin , J. N. H. Reek , Chem. Soc. Rev. 2015, 44, 433–448; 25340992 10.1039/c4cs00192c

[3j] H. Sesolis , J. Dubarle-Offner , C. K. M. Chan , E. Puig , G. Gontard , P. Winter , A. L. Cooksy , V. W. W. Yam , H. Amouri , Chem. Eur. J. 2016, 22, 8032–8037; 27142245 10.1002/chem.201601161

[3k] P. Das , A. Kumar , P. Howlader , P. S. Mukherjee , Chem. Eur. J. 2017, 23, 12565–12574; 28644555 10.1002/chem.201702263

[3l] I. A. Bhat , R. Jain , M. M. Siddiqui , D. K. Saini , P. S. Mukherjee , Inorg. Chem. 2017, 56, 5352–5360; 28394128 10.1021/acs.inorgchem.7b00449

[3m] R. W. Hogue , S. Singh , S. Brooker , Chem. Soc. Rev. 2018, 47, 7303–7338. 30124687 10.1039/c7cs00835j

[4a] A. M. Castilla , W. J. Ramsay , J. R. Nitschke , Acc. Chem. Res. 2014, 47, 2063–2073; 24793652 10.1021/ar5000924

[4b] J. R. Nitschke , Acc. Chem. Res. 2007, 40, 103–112. 17309191 10.1021/ar068185n

[5a] P. D. Frischmann , V. Kunz , V. Stepaneko , F. Würthner , Chem. Eur. J. 2015, 21, 2766–2769; 25582932 10.1002/chem.201405866

[5b] C. J. E. Haynes , J. Zhu , C. Chimerel , S. Hernández-Ainsa , I. A. Riddell , T. K. Ronson , U. F. Keyser , J. R. Nitschke , Angew. Chem. Int. Ed. 2017, 56, 15388–15392; 10.1002/anie.201709544 29024266

[5c] I. Sinha , P. S. Mukherjee , Inorg. Chem. 2018, 57, 4205–4221. 29578701 10.1021/acs.inorgchem.7b03067

[6a] M. C. Young , L. R. Holloway , A. M. Johnson , R. J. Hooley , Angew. Chem. Int. Ed. 2014, 53, 9832–9836; 10.1002/anie.201405242 25044629

[6b] W. J. Ramsay , F. J. Rizzuto , T. K. Ronson , K. Caprice , J. R. Nitschke , J. Am. Chem. Soc. 2016, 138, 7264–7267; 27213555 10.1021/jacs.6b03858

[6c] T. K. Ronson , B. S. Pilgrim , J. R. Nitschke , J. Am. Chem. Soc. 2016, 138, 10417–10420. 27500974 10.1021/jacs.6b06710

[7a] P. Mal , D. Schultz , K. Beyeh , K. Rissanen , J. R. Nitschke , Angew. Chem. Int. Ed. 2008, 47, 8297–8301; 10.1002/anie.200803066 18729112

[7b] D.-H. Ren , D. Qiu , C.-Y. Pang , Z. Li , Z.-G. Gu , Chem. Commun. 2015, 51, 788–791; 10.1039/c4cc08041f 25426503

[7c] N. Struch , G. Schnakenburg , R. Weisbarth , S. Klos , J. Beck , A. Lützen , Dalton Trans. 2016, 45, 14023–14029; 27534997 10.1039/c6dt02077a

[7d] N. Struch , C. Bannwarth , T. K. Ronson , Y. Lorenz , B. Mienert , N. Wagner , M. Engeser , E. Bill , R. Puttreddy , K. Rissanen , J. Beck , S. Grimme , J. R. Nitschke , A. Lützen , Angew. Chem. Int. Ed. 2017, 56, 4930–4935; 10.1002/anie.201700832 28370757

[7e] A. J. McConnell , Supramol. Chem. 2018, 30, 858–868.

[8a] R. A. Bilbeisi , J. K. Clegg , N. Elgrishi , X. de Hatten , M. Devillard , B. Breiner , P. Mal , J. R. Nitschke , J. Am. Chem. Soc. 2012, 134, 5110–5119; 22043943 10.1021/ja2092272

[8b] D. Lewing , H. Koppetz , F. E. Hahn , Inorg. Chem. 2015, 54, 7653–7659. 26161894 10.1021/acs.inorgchem.5b01334

[9a] X.-P. Zhou , Y. Wu , D. Li , J. Am. Chem. Soc. 2013, 135, 16062–16065; 24117433 10.1021/ja4092984

[9b] A. J. McConnell , C. M. Aitchison , A. B. Grommet , J. R. Nitschke , J. Am. Chem. Soc. 2017, 139, 6294–6297; 28426930 10.1021/jacs.7b01478 PMC5537689

[9c] N. Struch , F. Topić , K. Rissanen , A. Lützen , Dalton Trans. 2017, 46, 10809–10813; 28660950 10.1039/c7dt02182h

[9d] W. Meng , T. K. Ronson , J. K. Clegg , J. R. Nitschke , Angew. Chem. Int. Ed. 2013, 52, 1017–1021; 10.1002/anie.201206990 23238888

[9e] D. Samanta , P. S. Mukherjee , Chem. Eur. J. 2014, 20, 12483–12492; 25111071 10.1002/chem.201402553

[9f] N. Struch , F. Topić , G. Schnakenburg , K. Rissanen , A. Lützen , Inorg. Chem. 2018, 57, 241–250. 29236475 10.1021/acs.inorgchem.7b02412

[10a] W. M. Bloch , J. J. Holstein , W. Hiller , G. H. Clever , Angew. Chem. Int. Ed. 2017, 56, 8285–8289; 10.1002/anie.201702573 PMC5499718 28544072

[10b] M. M. J. Smulders , A. Jiménez , J. R. Nitschke , Angew. Chem. Int. Ed. 2012, 51, 6681–6685; 10.1002/anie.201202050 22674771

[10c] M. Fujita , N. Fujita , K. Ogura , K. Yamaguchi , Nature 1999, 400, 52–55;

[10d] A. J. McConnell , C. S. Wood , P. P. Neelakandan , J. R. Nitschke , Chem. Rev. 2015, 115, 7729–7793; 25880789 10.1021/cr500632f

[10e] W. Wang , Y.-X. Wang , H.-B. Yang , Chem. Soc. Rev. 2016, 45, 2656. 27009833 10.1039/c5cs00301f

[11a] K. Suzuki , M. Kawano , M. Fujita , Angew. Chem. Int. Ed. 2007, 46, 2819–2822; 10.1002/anie.200605084 17340660

[11b] D. M. Weekes , C. Diebold , P. Mobian , C. Huguenard , L. Allouche , M. Henry , Chem. Eur. J. 2014, 20, 5092–5101. 24644255 10.1002/chem.201304317

[12a] S. Chen , L.-J. Chen , H.-B. Yang , H. Tian , W. Zhu , J. Am. Chem. Soc. 2012, 134, 13596–13599; 22881042 10.1021/ja306748k

[12b] M. Han , R. Michel , B. He , Y.-S. Chen , D. Stalke , M. John , G. H. Clever , Angew. Chem. Int. Ed. 2013, 52, 1319–1323; 10.1002/anie.201207373 23208865

[13a] F. Reichel , J. K. Clegg , K. Gloe , K. Gloe , J. J. Weigand , J. K. Reynolds , C.-G. Li , J. R. Aldrich-Wright , C. J. Kepert , L. F. Lindoy , H.-C. Yao , F. Li , Inorg. Chem. 2014, 53, 688–690; 24393071 10.1021/ic402686s

[13b] S. M. Jansze , M. D. Wise , A. V. Vologzhanina , R. Scopelliti , K. Severin , Chem. Sci. 2017, 8, 1901–1908; 28567267 10.1039/c6sc04732g PMC5444114

[13c] J. Guo , Y.-W. Xu , K. Li , L.-M. Xiao , S. Chen , K. Wu , X.-D. Chen , Y.-Z. Fan , J.-M. Liu , C.-Y. Su , Angew. Chem. Int. Ed. 2017, 56, 3852–3856; 10.1002/anie.201611875 28247533

[13d] R. Saha , D. Samanta , A. J. Bhattacharyya , P. S. Mukherjee , Chem. Eur. J. 2017, 23, 8980–8986; 28471006 10.1002/chem.201701596

[13e] M. Hardy , N. Struch , F. Topić , G. Schnakenburg , K. Rissanen , A. Lützen , Inorg. Chem. 2018, 57, 3507–3515. 29185725 10.1021/acs.inorgchem.7b02516

[14a] W. J. Ramsay , F. T. Szczypiński , H. Weissman , T. K. Ronson , M. M. J. Smulders , B. Rybtchinski , J. R. Nitschke , Angew. Chem. Int. Ed. 2015, 54, 5636–5640; 10.1002/anie.201501892 25873434

[14b] Y. Yang , Y. Wu , J.-H. Jia , X.-Y. Zheng , Q. Zhang , K.-C. Xiong , Z.-M. Zhang , Q.-M. Wang , Cryst. Growth Des. 2018, 18, 4555–4561;

[14c] W.-K. Han , H.-X. Zhang , Y. Wang , W. Liu , X. Yan , T. Li , Z.-G. Gu , Chem. Commun. 2018, 54, 12646–12649. 10.1039/c8cc06652c 30357149

[15] M. A. Hoselton , L. J. Wilson , R. S. Drago , J. Am. Chem. Soc. 1975, 97, 1722–1729.

[16a] S. Schenker , A. Hauser , W. Wang , I. Y. Chan , J. Chem. Phys. 1998, 109, 9870–9878;

[16b] P. Adler , A. Hauser , A. Vef , H. Spiering , P. Gütlich , Hyperfine Interact. 1989, 47, 343–356.

[17] D. Schultz , J. R. Nitschke , Angew. Chem. Int. Ed. 2006, 45, 2453–2456; 10.1002/anie.200504447 16526088

[18a] A. Tissot , J.-F. Bardeau , E. Rivère , F. Brisset , M.-L. Boillot , Dalton Trans. 2010, 39, 7806–7812; 20652206 10.1039/c0dt00321b

[18b] C. Brewer , G. Brewer , C. Luckett , G. S. Marbury , C. Viragh , A. M. Beatty , W. R. Scheidt , Inorg. Chem. 2004, 43, 2402–2415. 15046517 10.1021/ic0351747 PMC1995556

[19] D. F. Evans , J. Chem. Soc. 1959, 2003–2005.

[20] E. Breuning , M. Ruben , J.-M. Lehn , F. Renz , Y. Garcia , V. Ksenofontov , P. Gütlich , E. Wegelius , K. Rissanen , Angew. Chem. Int. Ed. 2000, 39, 2504–2507; 10941118

[21a] C. Piguet , G. Bernardinelli , G. Hopfgartner , Chem. Rev. 1997, 97, 2005–2062; 11848897 10.1021/cr960053s

[21b] M. Albrecht , Chem. Soc. Rev. 1998, 27, 281–288;

[21c] M. Albrecht , Chem. Rev. 2001, 101, 3457–3497. 11840991 10.1021/cr0103672

[22] P. Gütlich , A. Hauser , H. Spiering , Angew. Chem. Int. Ed. Engl. 1994, 33, 2024–2054;

[23] A. Burkhardt , T. Pakendorf , B. Reime , J. Meyer , P. Fischer , N. Stübe , S. Panneerselvam , O. Lorbeer , K. Stachnik , M. Warmer , P. Rödig , D. Göries , A. Meents , Eur. Phys. J. Plus 2016, 131, 56.

[24] B. Olenyuk , A. Fechtenkötter , P. J. Stang , J. Chem. Soc. Dalton Trans. 1998, 1707–1728.

