# Supplementary material for: Dynamic Complex‐to‐Complex Transformations of Heterobimetallic Systems Influence the Cage Structure or Spin State of Iron(II) Ions
Source: Angew Chem Int Ed Engl. 2020 Jan 9;59(8):3195–200. doi: 10.1002/anie.201914629 (PMC7028022; doi:10.1002/anie.201914629)
Supplement: Supplementary file 1 — Supplementary [file ANIE-59-3195-s001.pdf]

Angewandte Chemie
Eine Zeitschrift der Gesellschaft Deutscher Chemiker

Supporting Information

Dynamic Complex-to-Complex Transformations of Heterobimetallic Systems Influence the Cage Structure or Spin State of Iron(II) Ions

*Matthias Hardy, Niklas Struch, Julian J. Holstein, Gregor Schnakenburg, Norbert Wagner, Marianne Engeser, Johannes Beck, Guido H. Clever, and Arne Lützen**

anie_201914629_sm_miscellaneous_information.pdf

Table of Contents

Table of Contents S1
General S2
Experimental Procedures S2
Synthesis of the organic ligand **1** S2
Syntheses of the complexes S3
Analytical Spectra S5
Crystallographic Data S23
Crystal structure of **ML-1**$(BF_4)_2$ S24
Crystal structure of **BP-1**$(OTf)_6(BF_4)_4$ S25
Crystal structure of **CU-1**$(BF_4)_{28}$ S26
Complex-to-Complex Transformations S28

General

All reagents and solvents were purchased from commercial sources and used as received without any further purification. NMR spectra were recorded on a Bruker Avance I 400, a Bruker Avance III 500, or a Bruker Avance I 500 spectrometer. Chemical shift values are reported relative to the residual solvent peak. ^{1}H-NMR data are reported as follows: chemical shift (δ) in ppm, multiplicity (s = singlet, bs = broad singlet (0.2 ppm < x < 0.5 ppm), vbs = very broad singlet (x > 0.5 ppm), d = doublet, pd = pseudo doublet, m = multiplet), coupling constant (J) in hertz (Hz), integral, correlation of the proton. Low and high-resolution electrospray ionization mass spectrometry (ESI-MS) spectra were recorded on a Bruker Daltonic LTQ Orbitrap XL or a Bruker Daltonic micro-TOF-Q. UV−vis spectra were recorded on a Specord 200 spectrometer (Analytik Jena AG) at ambient temperature. VSM measurements were performed on a Quantum Design (San Diego USA) PPMS-9 with VSM-Option. The sample was prepared within a VSM Powder Sample Holder (QD-P125E).
Experiments to determine the magnetic susceptibility in solution were performed using a coaxial insert in a NMR tube, using *tert.*-butanol (*t*-BuOH) as internal and external reference substance. The sample was filled into the NMR tube and the external standard into the insert.
2,5-Dibromo-6-methyl pyridine **4** was prepared as published before.[1]

Experimental Procedures

Synthesis of the organic ligand 1

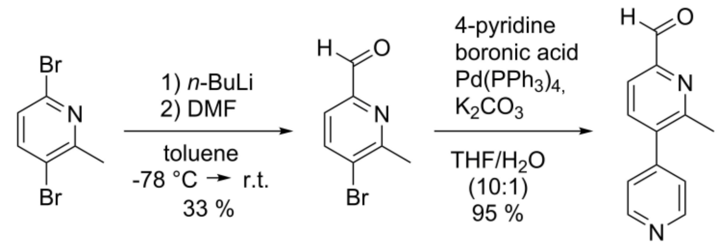

4 **5** **1**

Scheme S1. Synthesis of ligand building block 2-formyl-6-methyl-5-(4'-pyridyl) pyridine **1.**

A lithium-bromine exchange using *n*-BuLi in toluene as the solvent addressed the 2-position,[2] facilitating formylation by quenching with DMF to give **5** in a moderate yield. A Suzuki-Miyaura cross coupling with 4-pyridine boronic acid gave the desired ligand building block **1**.

5-Bromo-2-formyl-6-methyl pyridine 5

2,5-Dibromo-6-methyl pyridine **4** (1.50 g, 5.98 mmol, 1.00 equiv) was dissolved in 35 mL of dry toluene. The solution was cooled to -78 °C under an argon atmosphere and *n*-BuLi (2.5 M in hexane, 2.75 mL, 6.87 mmol, 1.50 equiv) was added dropwise over 15 minutes. After stirring at -78 °C for 2.5 hours, DMF (0.83 mL, 10.76 mmol, 1.80 equiv) was added dropwise. The mixture was stirred 1 hour at -78 °C and was then allowed to slowly warm up to room temperature. After stirring 1 hour at room temperature, 0.5 N hydrochloric acid (6 mL) was added dropwise to terminate the reaction. The aqueous phase was extracted with ethyl acetate (3 x 20 mL). The combined organic layers were dried with magnesium sulfate ($MgSO_4$) and the solvent was removed under reduced pressure. Purification by column chromatography on silica using a cyclohexane/ ethyl acetate/ triethylamine mixture (9:1:0.05, v/v) as the eluent yielded the product as an off-white solid in 33% yield (391.98 mg, 1.96 mmol).

^{1}H NMR (400 MHz, chloroform-d_1, 298 K): 10.00 (s, 1H, H-7), 7.99 (d, $^{3}J_{3,2}$ = 8.1 Hz, 1H, H-3), 7.65 (d, $^{3}J_{2,3}$ = 8.1 Hz, 1H, H-2), 2.76 (s, 3H, H-6). ^{13}C NMR (101 MHz, chloroform-d_1, 298 K): 192.8 (C-7), 158.5 (C-5), 151.0 (C-1), 141.0 (C-3), 127.1 (C-4), 120.4 (C-2), 25.0 (C-6).

---

1 S. Hameed P, V. Patil, S. Solapure, U. Sharma, P. Madhavapeddi, A. Raichurkar, M. Chinnapattu, P. Manjrekar, G. Shanbhag, J. Puttur, V. Shinde, S. Menasinakai, S. Rudrapatana, V. Achar, S. Awasthy, R. Nandishaiah, V. Humnabadkar, A. Ghosh, C. Narayan, V.K. Ramya, P. Kaur, S. Sharma, J. Werngren, S. Hoffner, V. Panduga, C.N.N. Kumar, J. Reddy, M. Kumar KN, S. Ganguly, S. Bharath, U. Bheemarao, K. Mukherjee, U. Arora, S. Gaonkar, M. Coulson, D. Waterson, V.K. Sambandamurthy, S.M. de Sousa, *J. Med. Chem.* **2014**, *57*, 4889–4905.

2 X. Wang, P. Rabbat, P. O'Shea, R. Tillyer, E.J.J. Grabowski, P.J. Reider, *Tetrahedron Lett*. **2000**, *41*, 4335– 4338.

The analytical data match the previously reported data.[1]

2-Formyl-6-methyl-5-(4'-pyridyl) pyridine 1

A suspension of **5** (50.00 mg, 0.25 mmol, 1.00 equiv), 4-pyridine boronic acid (36.87 mg, 0.30 mmol, 1.20 equiv), *tetrakis*(triphenylphosphine)palladium(0) (14.44 mg, 13.00 µmol, 0.05 equiv), and potassium carbonate (41.45 mg, 0.30 mmol, 1.20 equiv) in a solvent mixture of 6 mL of tetrahydrofuran and 0.6 mL of water was degassed three times and stirred under an argon atmosphere for 48 hours at 75 °C. After the reaction mixture was cooled to room temperature, a 1:1 mixture of a saturated sodium chloride solution and a disodium ethylenediaminetetraacetic acid solution was added. The aqueous layer was extracted with ethyl acetate (3 x 15 mL). The combined organic layers were dried with magnesium sulfate ($MgSO_4$), and the solvent was removed under reduced pressure. Purification by column chromatography on silica using a cyclohexane/ ethyl acetate/ triethylamine mixture (1:1:0.05, v/v) as the eluent yielded the desired compound as an off-white solid in 95% yield (47.65 mg, 0.24 mmol).

^{1}H NMR (500 MHz, acetonitrile-d_3, 298 K): 10.05 (s, 1H, H-1), 8.72 (pd, $^3J_{10,9}$ = 6.0 Hz, 2H, H-10), 7.89 (d, $^3J_{4,3}$ = 7.8 Hz, 1H, H-4), 7.82 (d, $^3J_{3,4}$ = 7.8 Hz, 1H, H-3), 7.45 (pd, $^3J_{9,10}$ = 6.0 Hz, 2H, H-9), 2.58 (s, 3H, H-7). ^{13}C NMR (126 MHz, acetonitrile-d_3, 298 K): 193.3 (C-1), 156.5 (C-6), 151.9 (C-2), 149.6 (C-10), 147.0 (C-8), 138.5 (C-5), 138.2 (C-4), 123.9 (C-9), 119.2 (C-3), 22.6 (C-7). ESI(+)-MS ([M] = $C_{12}H_{10}N_2O$): m/z 199.1 ({[M]+H}$^+$).

Syntheses of the complexes

Metalloligand ML-1(BF_4)$_2$

A solution of 2-formyl-6-methyl-5-(4'-pyridyl) pyridine **1** (50.00 mg, 0.25 mmol, 3.00 equiv) and *tris*(2-aminoethyl)amine (**2**, TREN; 12.59 µL, 0.08 mmol, 1.00 equiv) in 8 mL of acetonitrile was degassed by applying a vacuum and flushing with argon three times and stirred for 30 min at room temperature under an argon atmosphere. Subsequently, iron(II) tetrafluoroborate hexahydrate (28.38 mg, 0.08 mmol, 1.00 equiv) was added. The red solution was degassed again and stirred at 50 °C for 16 h under an argon atmosphere. After cooling to room temperature, the solution was transferred into 100 mL of degassed diethyl ether. The red precipitant was collected and carefully washed with diethyl ether several times. After drying in air, the product was obtained as a bright red solid in 92% yield (73.12 mg, 0.08 mmol).

^{1}H NMR (300 MHz, acetonitrile-d_3, 298 K): 236.97 (bs), 169.28 (bs), 68.86 (bs), 44.71 (vbs), 36.45 (bs), 8.92 (bs), 6.89 (bs), -7.16 (bs), -13.32 (bs). ESI(+)-MS ([M] = [$C_{42}H_{42}FeN_{10}$]$^{2+}$): m/z 829.29 ({[M] + BF_4}$^+$), 761.29 ({[M] + F}$^+$), 371.15 ({[M]}$^{2+}$). HRMS. Calcd for {[M]}$^{2+}$: m/z 371.1465. Found: m/z 371.1474. UV−vis [CH_3CN, 170.5 µM, 10 mm cuvette; λ (nm)]: 298, 503.

Trigonal bipyramid BP-1(OTf)$_6$(BF_4)$_4$

A solution of **ML-1**(BF_4)$_2$ (10.00 mg, 10.90 µmol, 2.00 equiv) and 1,3-*bis*(diphenylphosphino)propanylpalladium(II) triflate ([Pd(dppp)(OTf)$_2$]; 13.37 mg, 16.40 µmol, 3.00 equiv) in 1.4 mL of acetonitrile was degassed by applying a vacuum and flushing with argon three times and heated under an argon atmosphere at 50 °C for 16 h. The resulting solution was filtered, and the product was precipitated by the diffusion of diethyl ether vapor into an acetonitrile solution of the complex. The red solid was filtered off and carefully washed with diethyl ether several times. After the solid was dried in a stream of air, the product was obtained as a red solid in 94% yield (22.18 mg, 5.08 µmol).

^{1}H NMR (300 MHz, acetonitrile-d_3, 298 K): 237.65 (bs), 171.52 (bs), 66.83 (bs), 44.25 (vbs), 36.19 (bs), 8.78 (bs), 7.87 (m), 6.70 (m), 3.19 (m), 2.91-2.86 (m), -6.75 (bs), -14.01 (bs). ESI(+)-MS ([M] = [$C_{165}H_{162}Fe_2N_{20}P_6Pd_3$]$^{10+}$): m/z 2102.2 ({[M]$_2$ + BF_4 + 15 OTf}$^{4+}$), 2071.2 ({[M]$_2$ + 3BF_4 + 13OTf }$^{4+}$), 2055.5 ({[M] + 2BF_4 + 6OTf}$^{2+}$; {[M]$_2$ + 4BF_4 + 12OTf}$^{4+}$), 2039.7 ({[M]$_2$ + 5BF_4 + 11OTf}$^{4+}$), 2024.0 ({[M] + 3BF_4 + 5OTf}$^{2+}$; {[M]$_2$ + 6BF_4 + 10OTf }$^{4+}$), 2008.2 ({[M]$_2$ + 7BF_4 + 9OTf}$^{4+}$), 1993.0 ({[M] + 4BF_4 + 4OTf}$^{2+}$; ({[M]$_2$ + 8BF_4 + 8OTf}$^{4+}$), 1977.3 ({[M]$_2$ + 9BF_4 + 7OTf}$^{4+}$), 1961.5 ({[M] + 5BF_4 + 3OTf}$^{2+}$; {[M]$_2$ + 10BF_4 + 6OTf}$^{4+}$), 1362.0 ({[M] + 7OTf}$^{3+}$), 1341.4 ({[M] + BF_4 + 6OTf}$^{3+}$), 1320.4 ({[M] + 2BF_4 + 5OTf}$^{3+}$), 1299.4 ({[M] + 3BF_4 + 4OTf}$^{3+}$), 1278.8 ({[M] + 4BF_4 + 3OTf}$^{3+}$), 1268.5 ({[M]$_2$ + 9BF_4 + 5OTf}$^{6+}$), 1258.4 ({[M] + 5BF_4 + 2OTf}$^{3+}$), 1089.2 ({[M] – Pd(dppp) + 5OTf}$^{3+}$), 1068.5 ({[M] – Pd(dppp) + BF_4 + 4OTf}$^{3+}$), 1047.86 ({[M] – Pd(dppp) + 2BF_4 + 3OTf}$^{3+}$), 1027.2 ({[M] – Pd(dppp) + 3BF_4 + 2OTf}$^{3+}$), 984.4 ({[M] + 6OTf}$^{4+}$), 953.3 ({[M] + 2BF_4 + 4OTf}$^{4+}$), 937.3 ({[M] + 3BF_4 + 3OTf}$^{4+}$), 921.8 ({[M] + 4BF_4 + 2OTf}$^{4+}$), 891.4 ({[**ML-1**] + OTf}$^+$), 829.4 ({[**ML-1**] + BF_4}$^+$), 761.4 ({[**ML-1**] + F}$^+$), 667.1 ({[Pd(dppp)] + OTf}$^+$), 371.2 ({[**ML-1**]}$^{2+}$), 259.1 ({[Pd(dppp)]}$^{2+}$). HRMS. Calcd for {[M] + 2BF_4 + 5OTf}$^{3+}$: m/z 1320.1758. Found: m/z 1320.1771. UV−vis [CH_3CN, 3821 µM, 0.01 mm cuvette; λ (nm)]: 269, 299, 505.

Cube CU-1(BF_4)$_{28}$

A solution of **ML-1**(BF_4)$_2$ (5.00 mg, 5.46 μmol, 8.00 equiv) and *tetrakis*(acetonitrile)palladium(II) tetrafluoroborate ([Pd(CH_3CN)$_4$](BF_4)$_2$; 1.82 mg, 4.09 μmol, 6.00 equiv) in 0.7 mL of acetonitrile was degassed by applying a vacuum and flushing with argon three times and heated under an argon atmosphere at 50 °C for 16 hours. The resulting solution was filtered, and the product was precipitated by the diffusion of diethyl ether vapor into an acetonitrile solution of the complex. The red solid was filtered off and carefully washed with diethyl ether several times. After the solid was dried in a stream of air, the product was obtained as a bright red solid in 89% yield (5.49 mg, 0.61 μmol).

^{1}H NMR (400 MHz, acetonitrile-*d*$_3$, 298 K): 238.81 (bs), 166.73 (bs), 75.03 (bs), 46.08 (vbs), 34.30 (bs), 8.72 (bs), 7.88-7.69 (m), 5.66 (bs), -6.75 (bs), -13.09 (bs). ESI(+)-MS ([M] = $[C_{336}H_{336}Fe_8N_{80}Pd_6]^{28+}$): m/z 1714.996 ($\{[M] + 23BF_4\}^{5+}$), 1415.333 ($\{[M] + 22BF_4\}^{6+}$), 1200.713 ($\{[M] + 21BF_4\}^{7+}$), 1039.498 ($\{[M] + 20BF_4\}^{8+}$), 914.665 ($\{[M] + 19BF_4\}^{9+}$), 813.998 ($\{[M] + 18BF_4\}^{10+}$), 732.713 ($\{[M] + 17BF_4\}^{11+}$), 663.999 ($\{[M] + 16BF_4\}^{12+}$), 606.384 ($\{[M] + 15BF_4\}^{13+}$). HRMS. Calcd for $\{[M] + 21BF_4\}^{7+}$: m/z 1200.409. Found: m/z 1200.429. UV−vis [CH_3CN, 1081 μM, 0.01 mm cuvette; λ (nm)]: 261, 301, 504.

Analytical Spectra

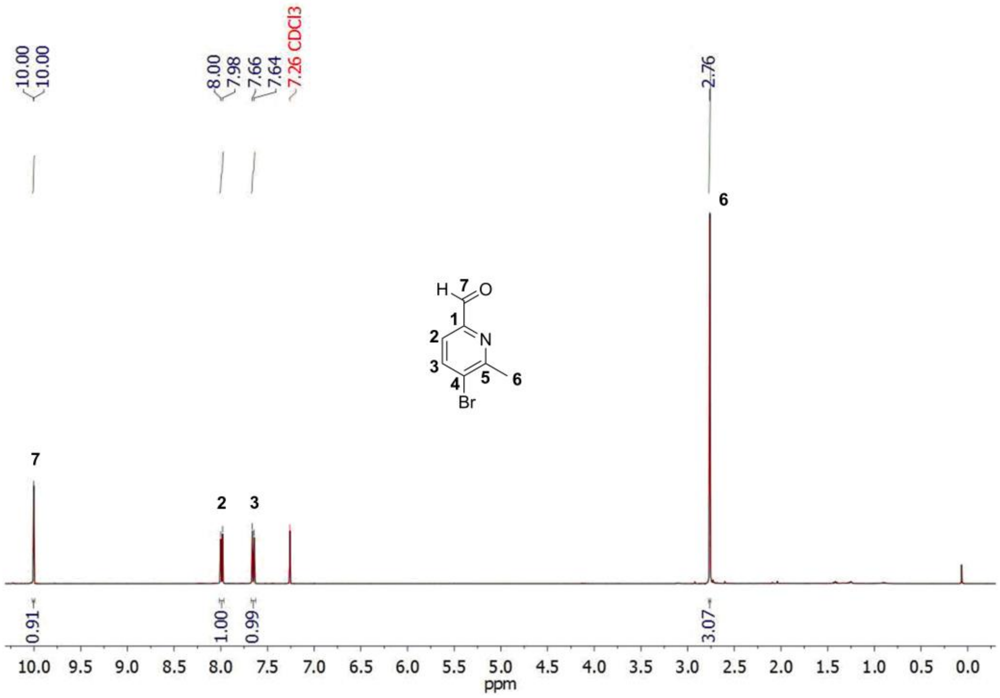

Figure S1. ^{1}H-NMR (400 MHz, chloroform-d_1, 298 K) of 5-bromo-2-formyl-6-methyl pyridine **5**.

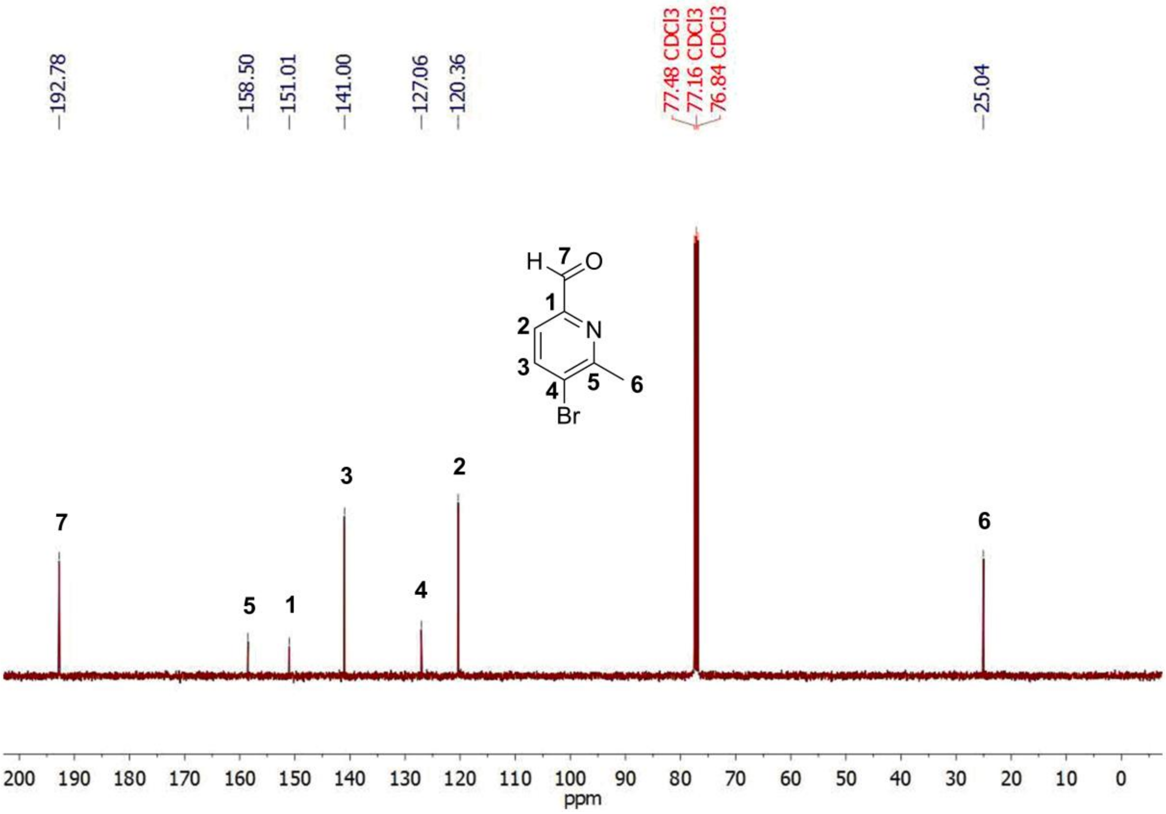

Figure S2. ^{13}C-NMR (101 MHz, chloroform-d_1, 298 K) of 5-bromo-2-formyl-6-methyl pyridine **5**.

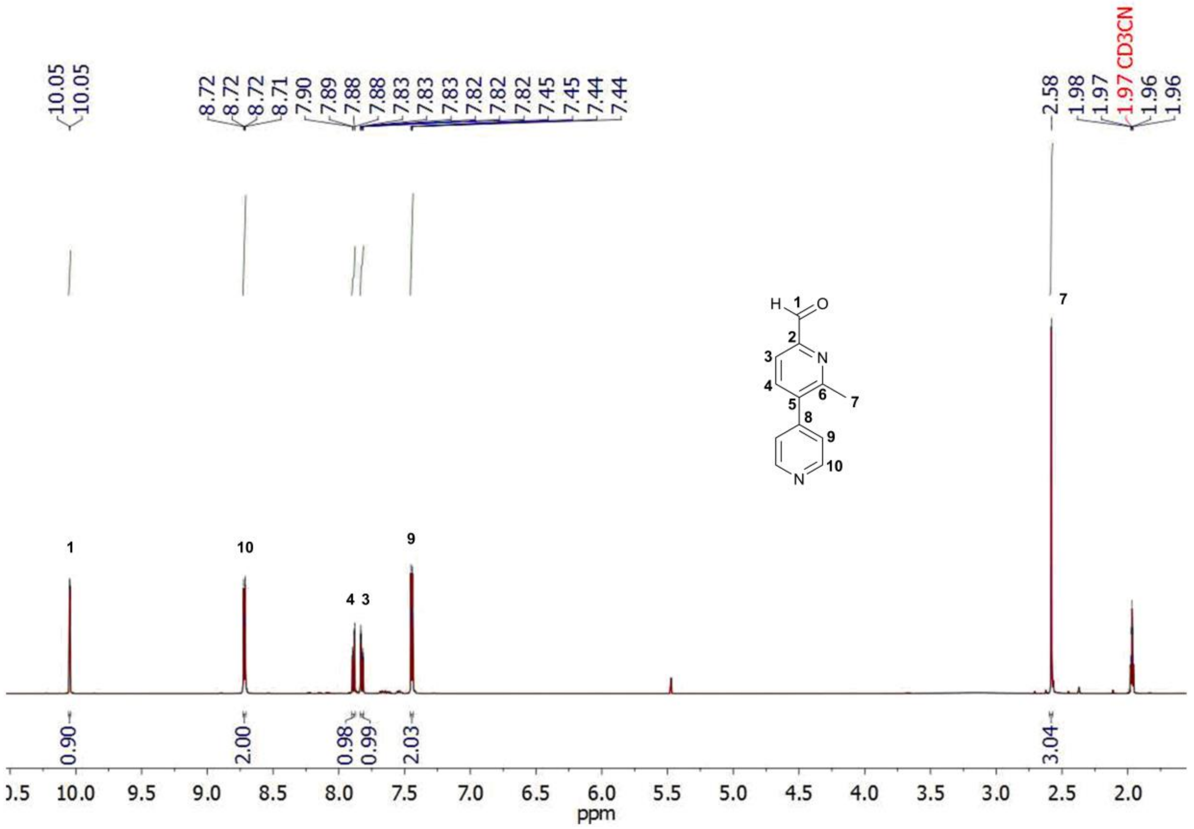

Figure S3. ^{1}H-NMR (500 MHz, acetonitrile-d_3, 298 K) of 2-formyl-6-methyl-5-(4'-pyridyl) pyridine **1**.

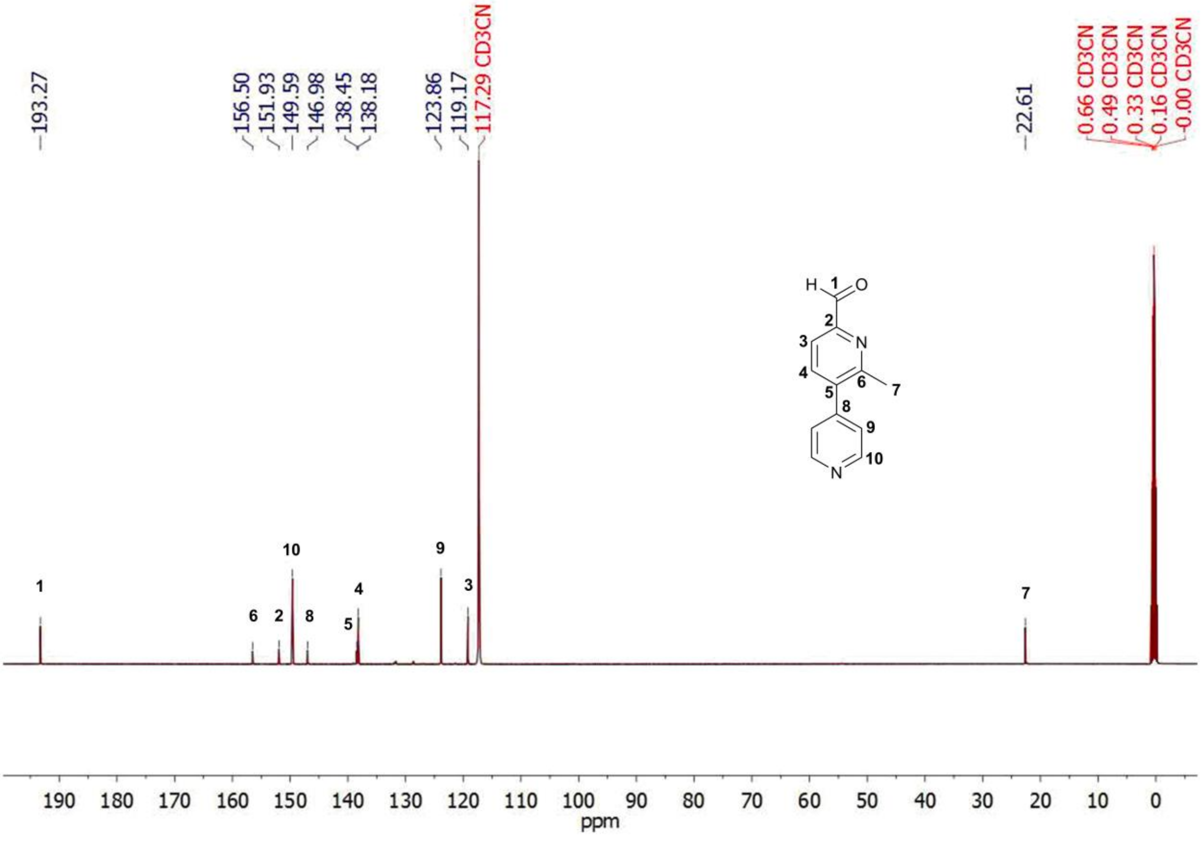

Figure S4. ^{13}C-NMR (126 MHz, acetonitrile-d_3, 298 K) of 2-formyl-6-methyl-5-(4'-pyridyl) pyridine **1**.

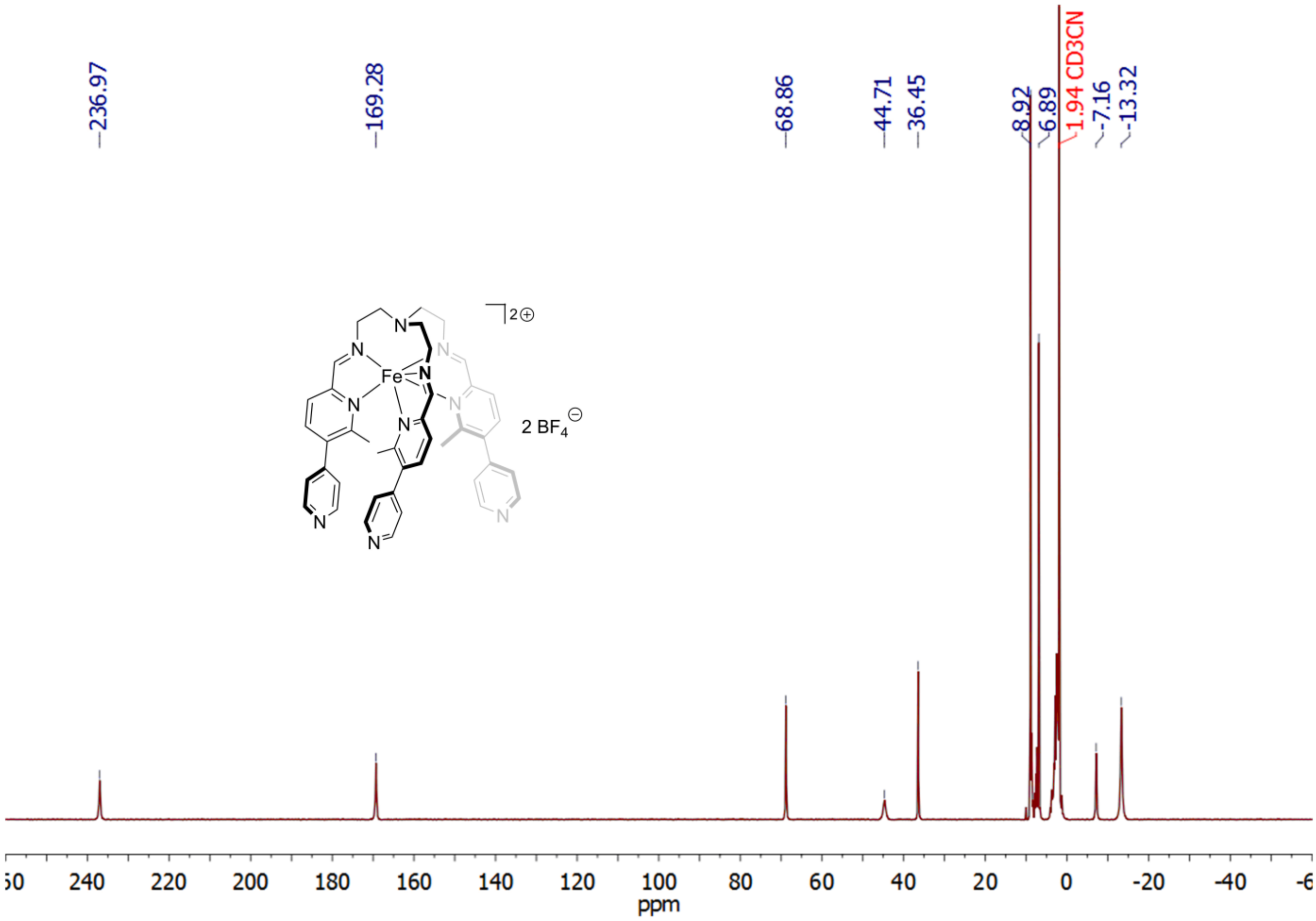

Figure S5. Paramagnetic ^{1}H-NMR spectrum (300 MHz, acetonitrile-d_3, 298 K) of **ML-1**$(BF_4)_2$.

The ^{1}H-NMR spectrum of **ML-1(**$BF_4)_2$ shows only one set of signals, indicating the presence of C_3-symmetric complexes. As dictated by the covalently bridged ligand system, only the *fac*-isomer of the mononuclear complex can be formed and observed.

The chemical shift of $\delta = 237.0$ ppm is consistent with the predicted chemical shift for imine protons in paramagnetic iron(II) compounds with S = 2.[3]

The magnetic susceptibility of **ML-1**$(BF_4)_2$ in solution at room temperature was obtained by employing the Evans' method as described in the literature.[4] The molar magnetic susceptibility multiplied with temperature was determined to be 3.07 cm^3 K mol^{-1} (Table S1, Figure S6) and is consistent with an expected value for X_mT of one uncoupled iron(II) cation in the high-spin state (S = 2) of 3.001 cm^3 K mol^{-1}.[5]

Table S1: Magnetic susceptibility of **ML-1**$(BF_4)_2$ in acetonitrile-d_3 as determined by the Evans' method.

concentration [mM]	temperature [K]	Δδ (*t*-BuOH) [Hz]	X_mT [cm^3 K mol^{-1}]
9.49	298	123.07	3.07

[3] W.C. Isley III, S. Zarra, R.K. Carlson, R.A. Bilbesi, T.K. Ronson, J.R. Nitschke, L. Gagliardi, C.J. Cramer, *Phys. Chem. Chem. Phys.* **2014**, *16*, 10620–10628.
[4] D.F. Evans, *J. Chem. Soc.* **1959**, 2003 – 2005.
[5] E. Breuning, M. Ruben, J.-M. Lehn, F. Renz, Y. Garcia, V. Ksenofontov, P. Gütlich, E. Wegelius, K. Rissanen, *Angew. Chem. Int. Ed.* **2000**, *39*, 2504 – 2507; *Angew. Chem.* **2000**, *112*, 2563 – 2566.

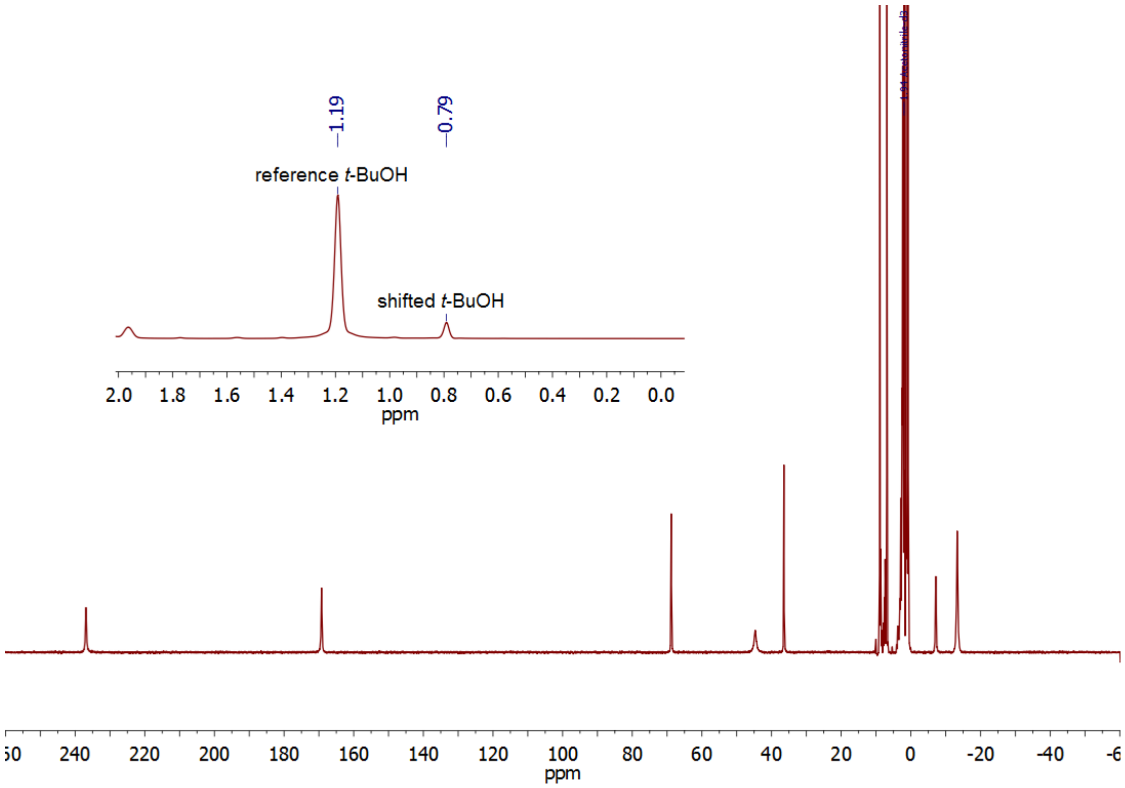

Figure S6. Evans' experiment (300 MHz, acetonitrile-d_3, 298 K) of **ML-1**(BF_4)$_2$.

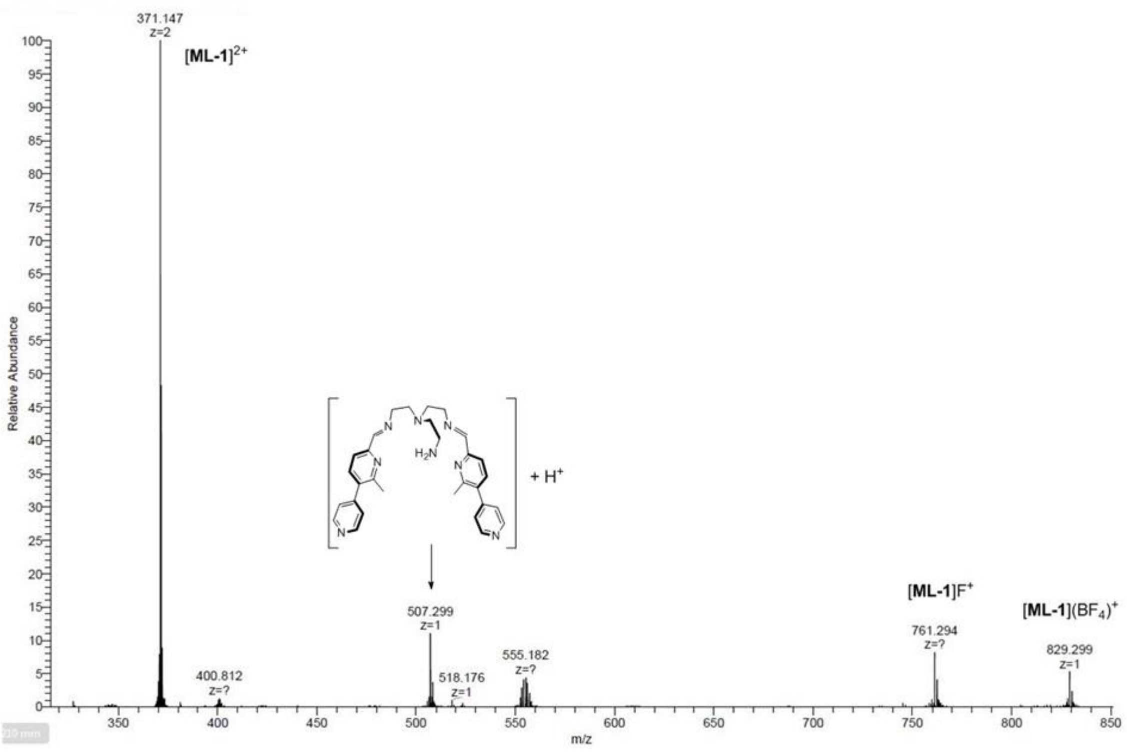

Figure S7. ESI(+)-MS spectrum (from acetonitrile) of **ML-1**(BF_4)$_2$.

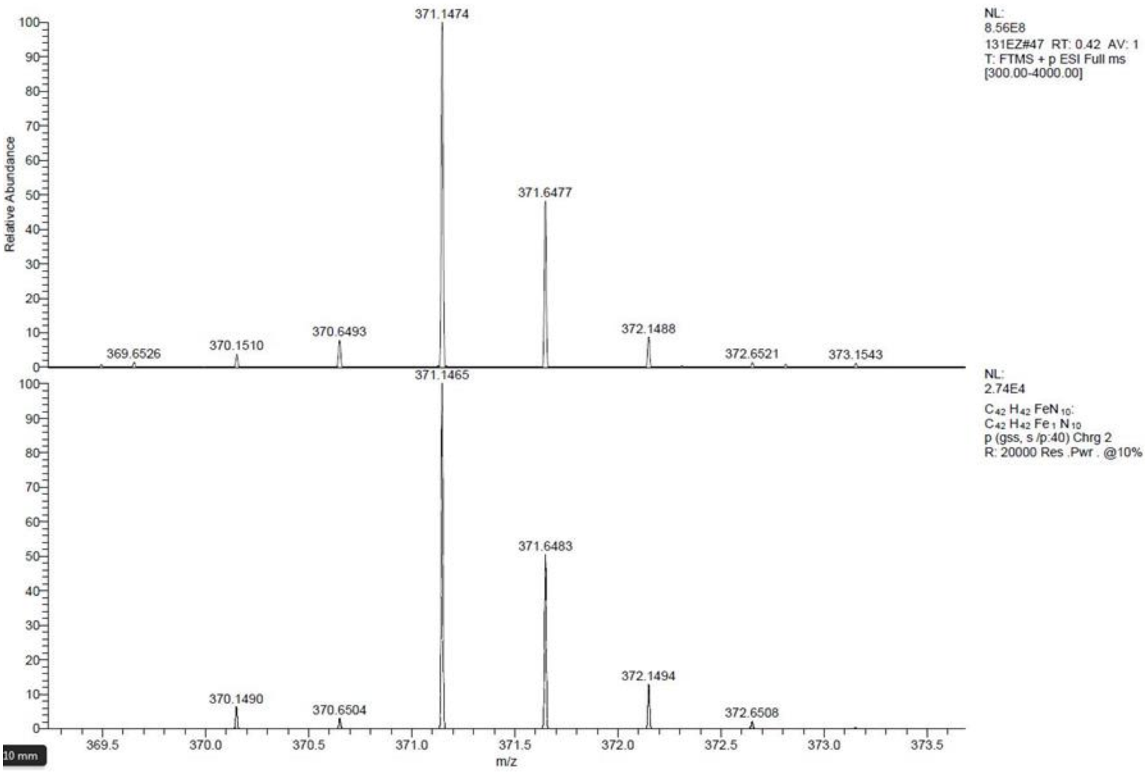

Figure S8. Exact mass of **ML-1**$^{2+}$. Top: Observed spectrum. Bottom: Calculated spectrum.

The ESI-MS spectrum shows a signal for {[**ML-1**]F}$^{+}$. Fluoride results from the fragmentation of a tetrafluoridoborate anion.

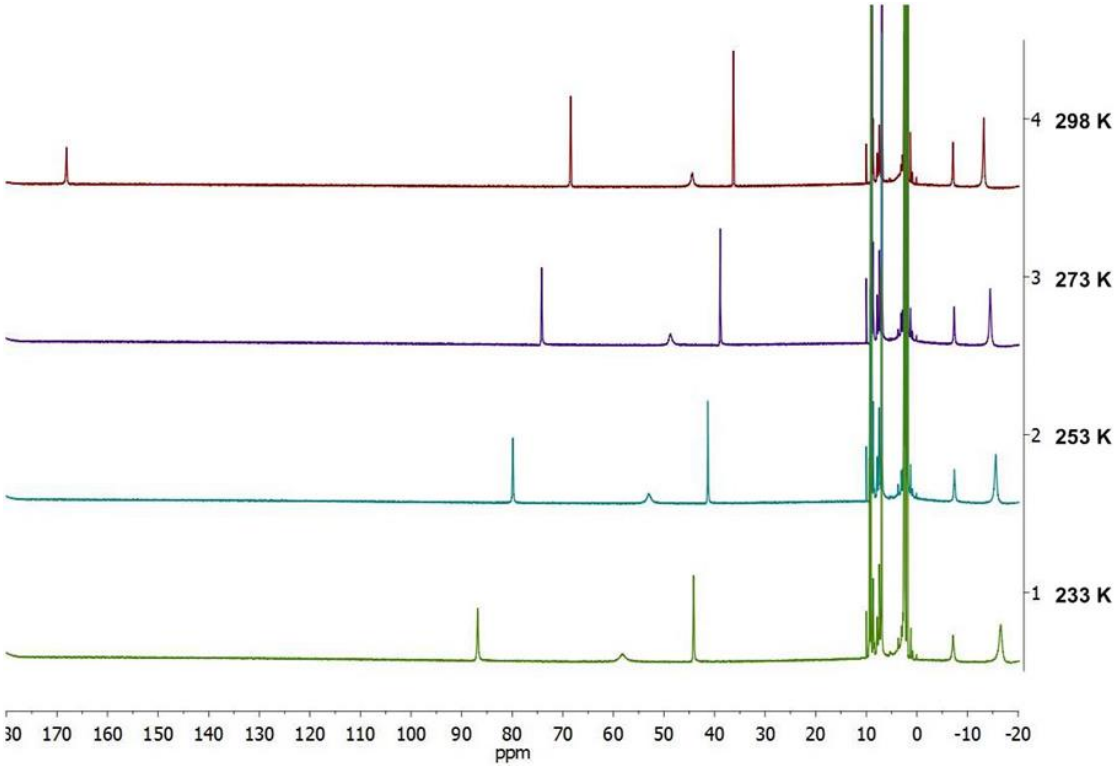

Figure S9. Temperature dependent paramagnetic ^{1}H-NMR spectra (500 MHz, acetonitrile-d_3, 298 K) of **ML-1**$(BF_4)_2$.

The temperature dependent ^{1}H-NMR spectra of **ML-1**$(BF_4)_2$ (Figure S9) show that the paramagnetic high spin state of the iron(II) cation is stabilized in the temperature range from 233 K to 298 K in solution. Please note that chemical shifts of paramagnetic samples are temperature-dependent and the following dependency is valid in these cases: $\delta T \sim XT$ (δ = chemical shift, T = temperature, X = magnetic susceptibility). Therefore, the product δT is constant, if the magnetic susceptibility does not change (Table S2).

Table S2. Comparison of δT values for one selected ^{1}H-NMR signal of **ML-1**$(BF_4)_2$. The observed variation of the product δT is below 1 %, proving that the paramagnetic high-spin state is stabilized in the given temperature range.

Temperature [K]	Chemical shift [ppm]	δT [ppm K]
233	86.8	20241
253	79.9	20233
273	74.2	20264
298	68.4	20376

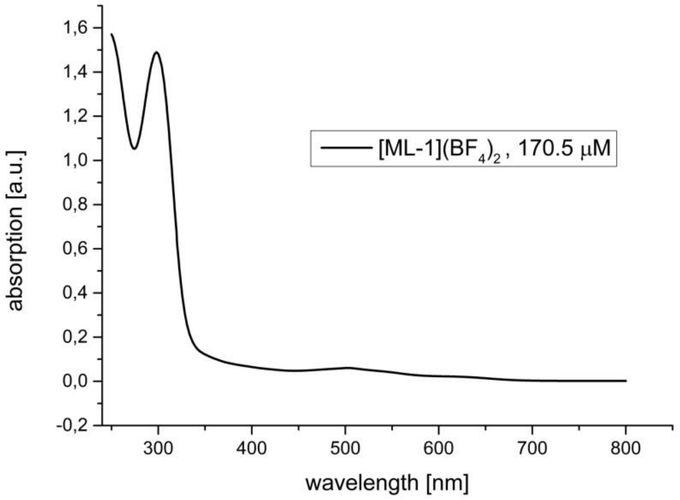

Figure S10. UV-vis spectrum of **ML-1**$(BF_4)_2$ (acetonitrile, 170.5 µM, 10 mm cuvette).

A crystalline sample of **ML-1**$(BF_4)_2$ was used to determine the magnetic properties in the solid state (Figure S11). During sample preparation we observed a very rapid loss of solvent after removing the crystals from the mother liquor, which hindered the exact determination of the mass of the sample. As a result we observed a molar magnetic susceptibility multiplied with temperature of 3.5 cm^3 K mol^{-1} in the temperature range from 100 to 300 K, which is slightly higher than the expected value for one uncoupled iron(II) cation in the high-spin state of X_mT = 3.001 cm^3 K mol^{-1}.[5] However, consistent with the result of the determination of the magnetic susceptibility in solution (Table S1), the measurement shows the paramagnetic character of the complex.

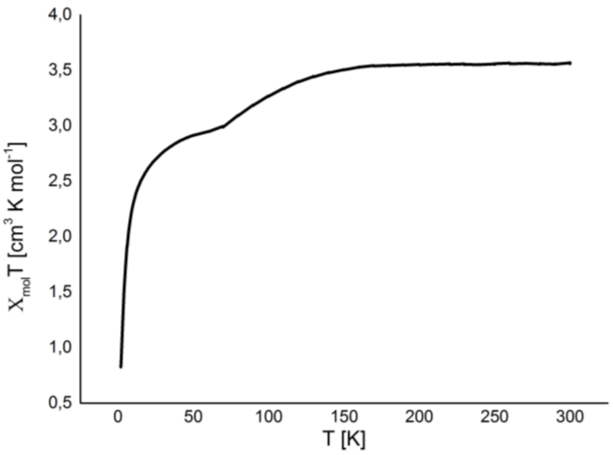

Figure S11. Temperature dependent VSM measurement (10000 Oe) of a crystalline sample of **ML-1**$(BF_4)_2$.

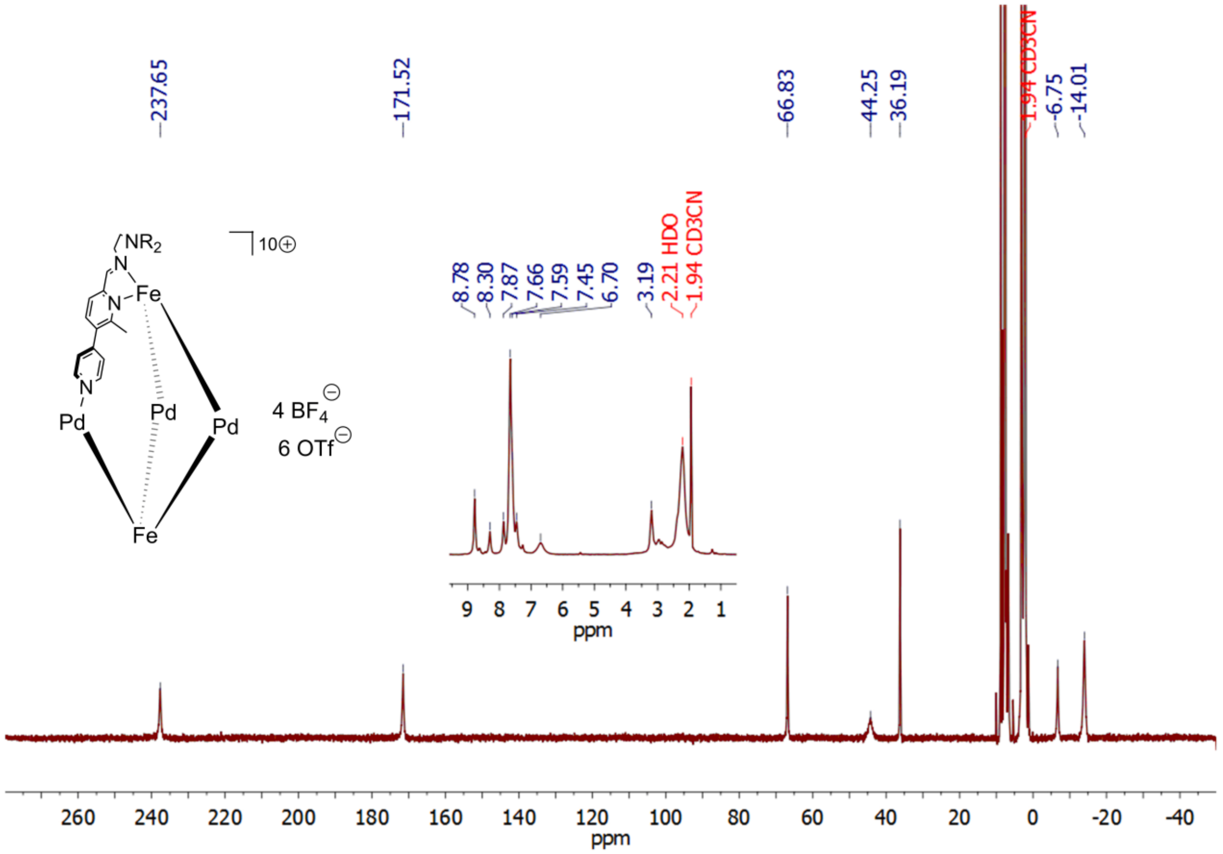

Figure S12. Paramagnetic ^{1}H-NMR spectrum (300 MHz, acetonitrile-d_3, 298 K) of **BP-1**(OTf)$_6$(BF$_4$)$_4$.

The observed chemical shift of δ = 237.7 ppm is consistent with the predicted chemical shift for imine protons in paramagnetic iron(II) compounds with S = 2.[3]
The magnetic susceptibility of **BP-1**(OTf)$_6$(BF$_4$)$_4$ in solution at room temperature was obtained by employing the Evans' method as described in the literature.[4] The molar magnetic susceptibility multiplied with temperature was determined to be 5.95 cm^3 K mol^{-1} (Table S3, Figure S13) and is consistent with an expected value for X_mT of two uncoupled iron(II) cations in the high-spin state (S = 2) of 6.002 cm^3 K mol^{-1}.[5]

Table S3: Magnetic susceptibility of **BP-1**(OTf)$_6$(BF$_4$)$_4$ in acetonitrile-d_3 as determined by the Evans' method.

concentration [mM]	temperature [K]	Δδ (*t*-BuOH) [Hz]	X_mT [cm^3 K mol^{-1}]
4.79	298	120.06	5.95

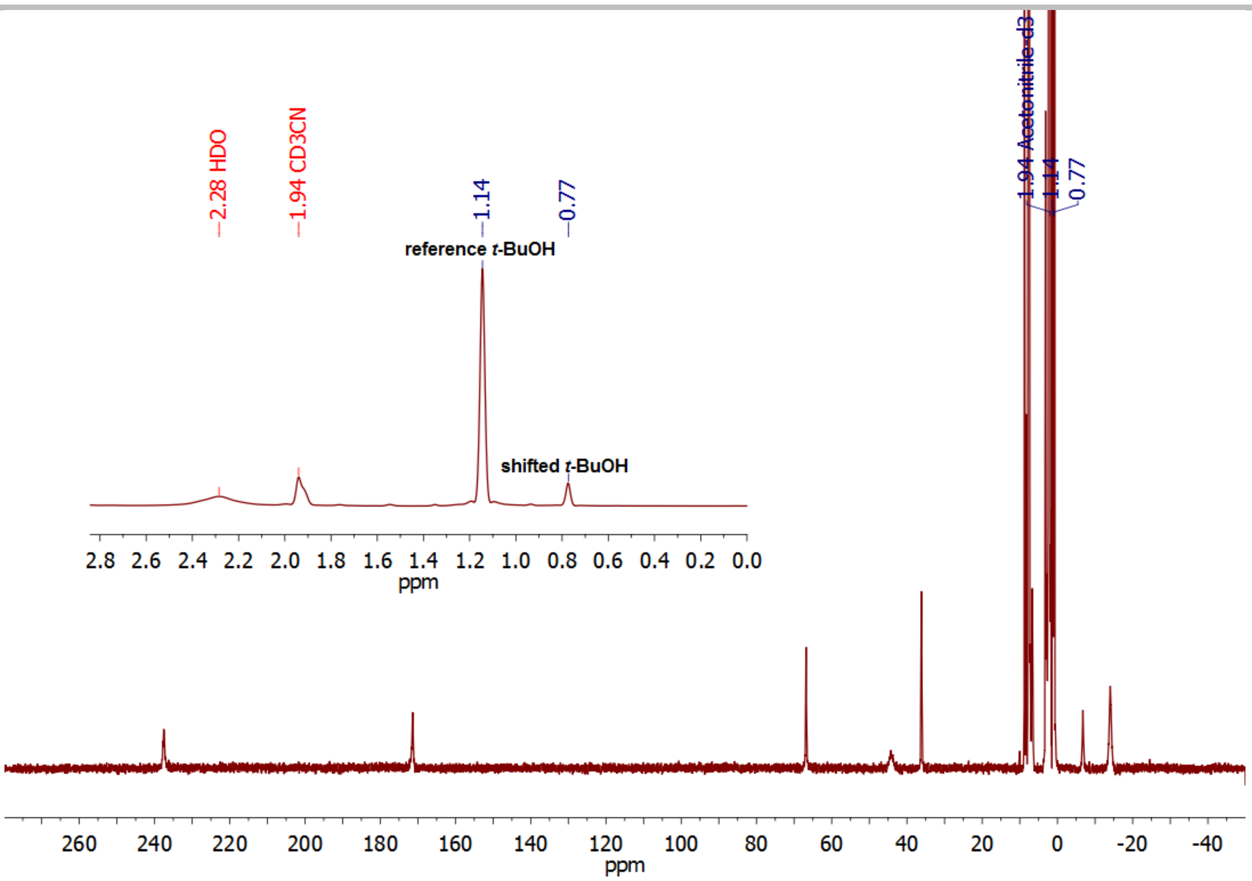

Figure S13. Evans' experiment (300 MHz, acetonitrile-d_3, 298 K) of **BP-1**(OTf)$_6$(BF$_4$)$_4$.

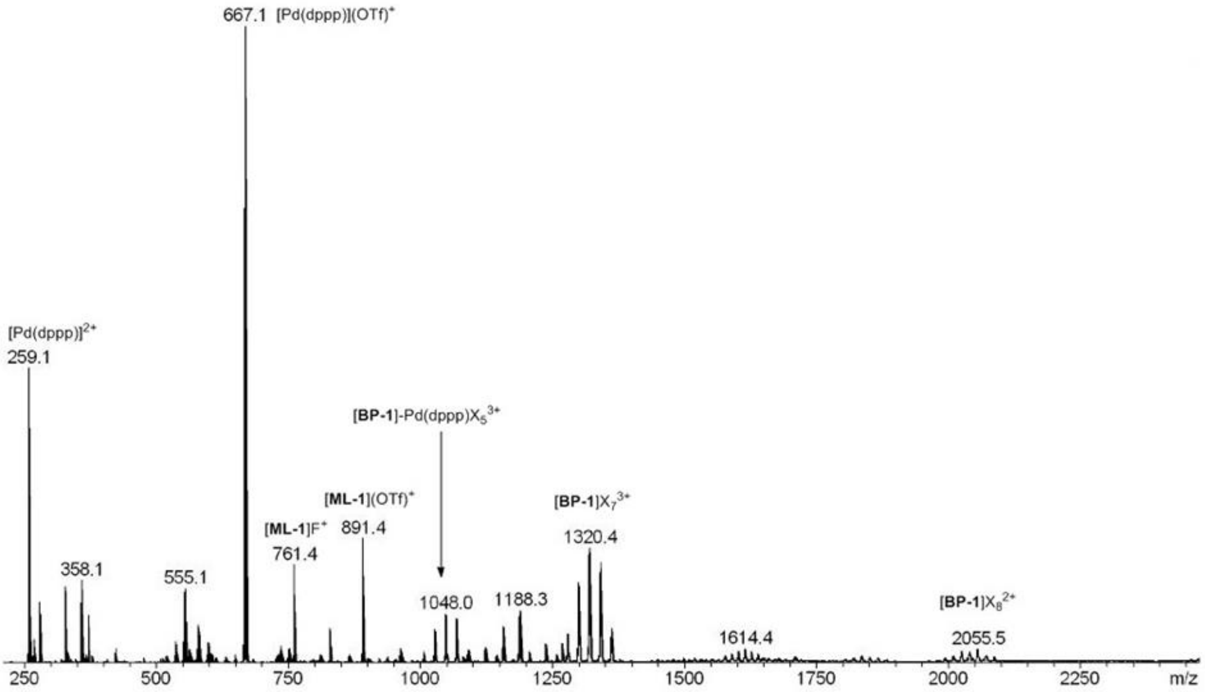

Figure S14. ESI(+)-MS spectrum (from acetonitrile) of **BP-1**(OTf)$_6$(BF$_4$)$_4$.

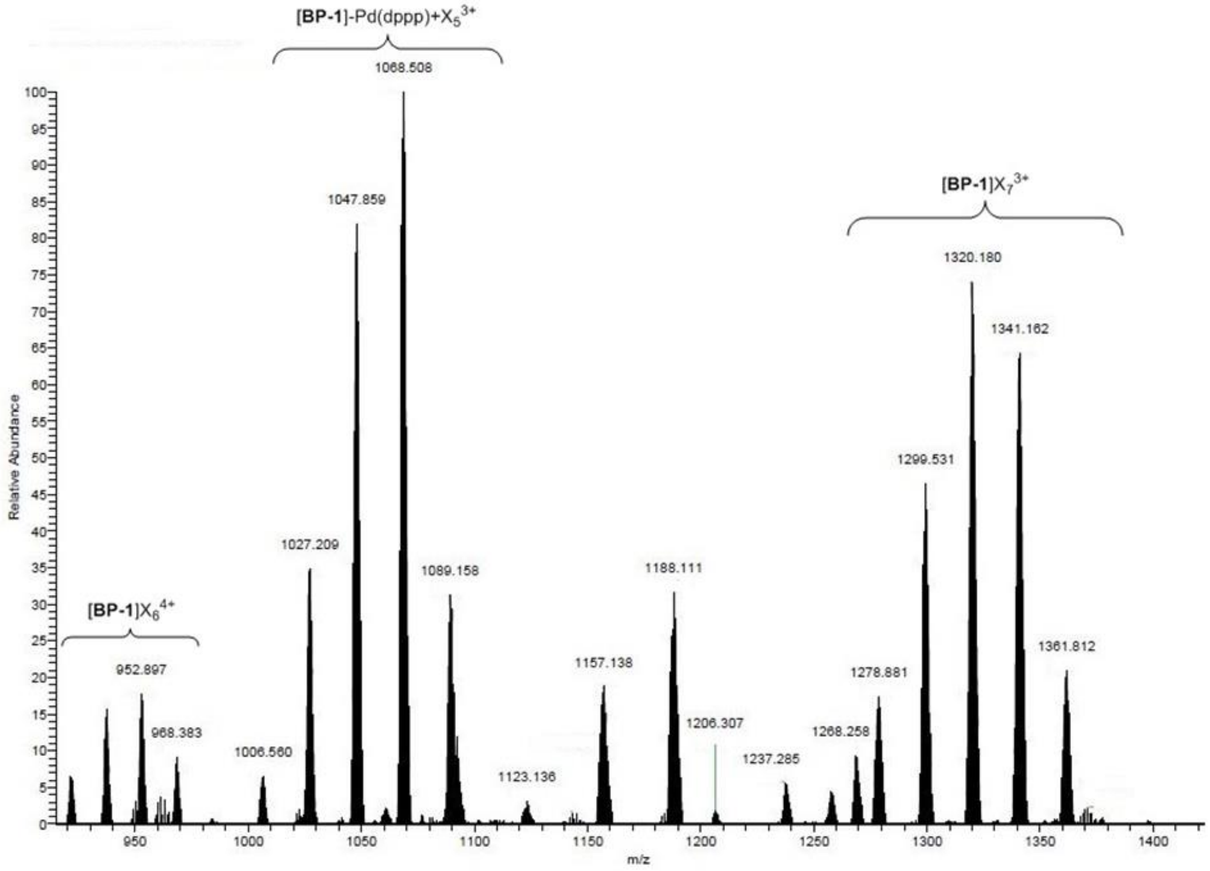

Figure S15. Excerpt of the ESI(+)-MS spectrum (from acetonitrile) of **BP-1**$(OTf)_6(BF_4)_4$.

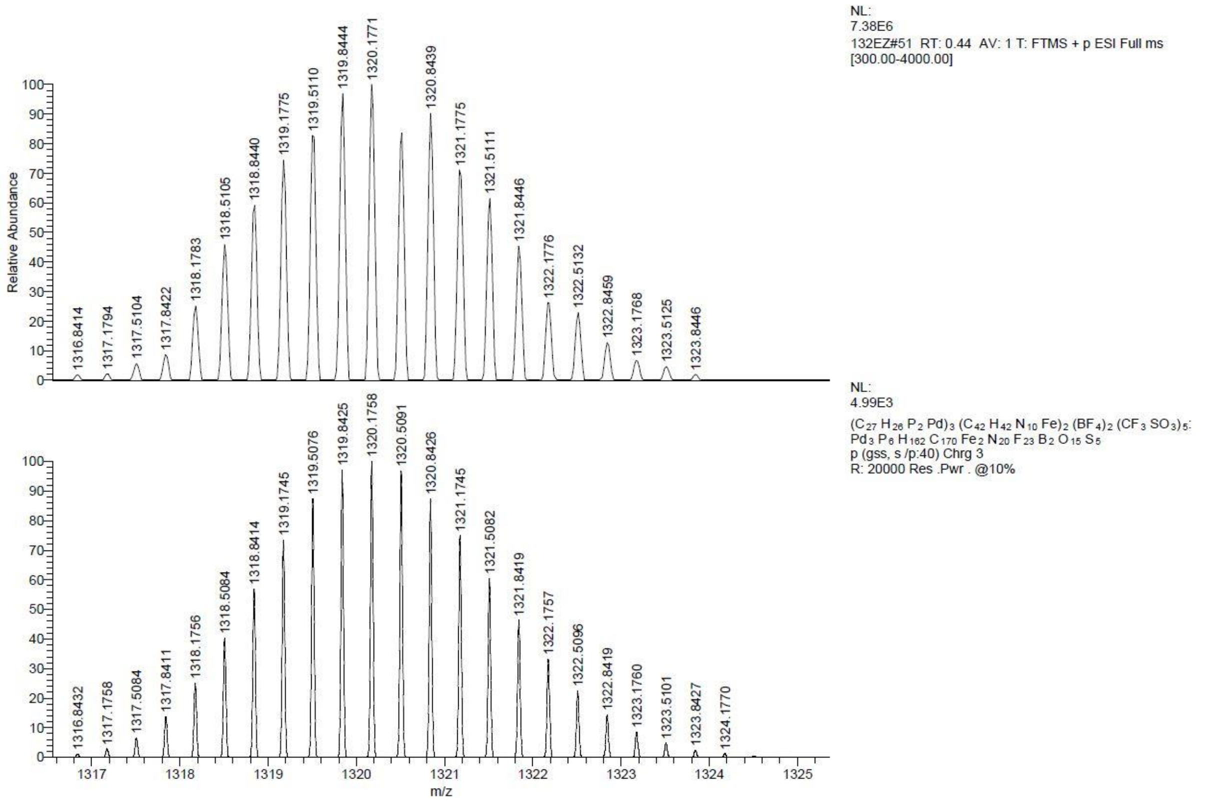

Figure S16. Exact mass of **BP-1**$(OTf)_5(BF_4)_2^{3+}$. Top: Observed spectrum. Bottom: Calculated spectrum.

The ESI-MS spectrum of **BP-1**$(OTf)_6(BF_4)_4$ (Figures S14 and S15) shows that the complex easily fragments with loss of one Pd(dppp)$^{2+}$ building block. This fragmentation probably is a consequence of the rather weak Pd-N coordination bonds and the resulting kinetic lability of the complex, also corroborated by UV-vis spectroscopy (vide infra, Figure S18 and Figure S19).

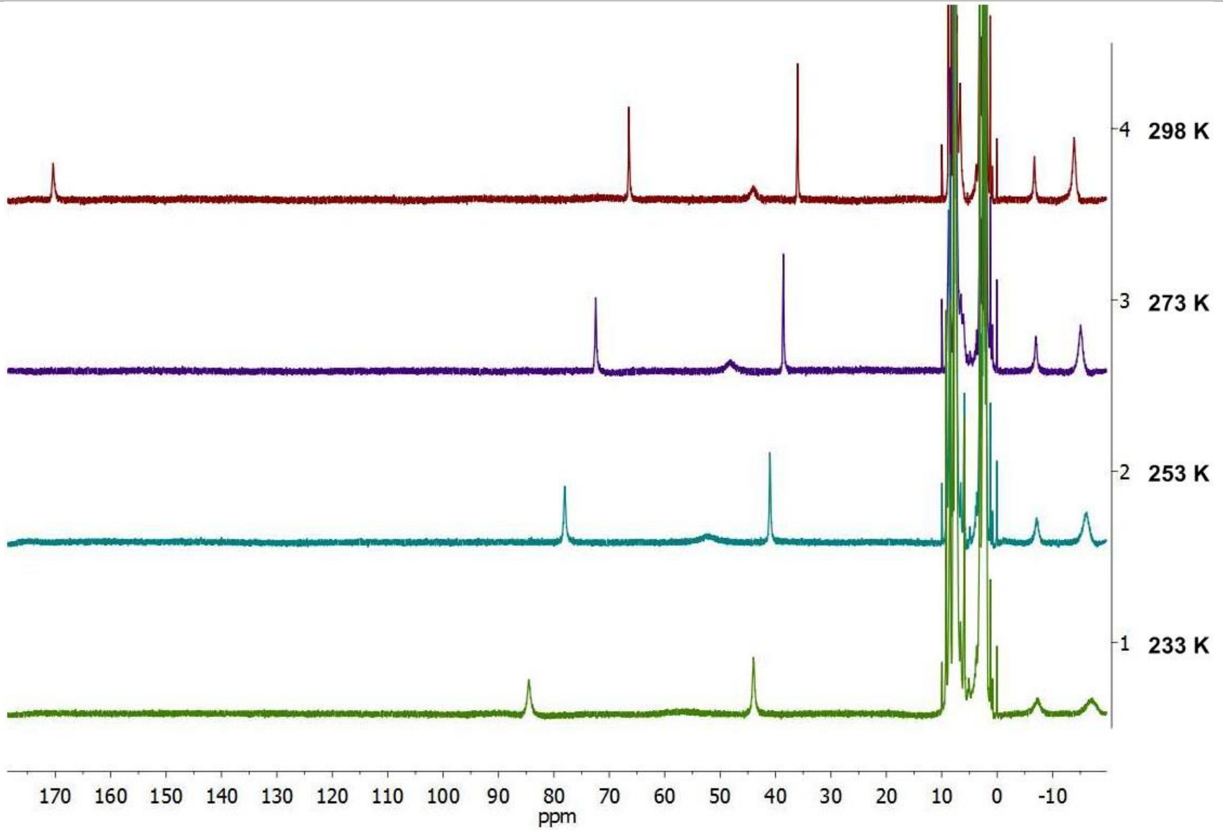

Figure S17. Temperature dependent wide-sweep ^{1}H-NMR spectra (500 MHz, acetonitrile-d_3, 298 K) of **BP-1**$(OTf)_6(BF_4)_4$.

The temperature dependent ^{1}H-NMR spectra of **BP-1**$(OTf)_6(BF_4)_4$ (Figure S17) show that the paramagnetic high spin state of the iron(II) cations is stabilized in the temperature range from 233 K to 298 K in solution. Analogue to the findings with **ML-1**$(BF_4)_2$ (Figure S9, Table S2) the product δT is constant, if the magnetic susceptibility does not change (Table S4).

Table S4. Comparison of δT values for one selected ^{1}H-NMR signal of **BP-1**$(OTf)_6(BF_4)_4$. The observed variation of the product δT is 1 %, proving that the paramagnetic high-spin state is stabilized in the given temperature range.

Temperature [K]	Chemical shift [ppm]	δT [ppm K]
233	84.5	19672
253	78.0	19742
273	72.5	19800
298	66.5	19817

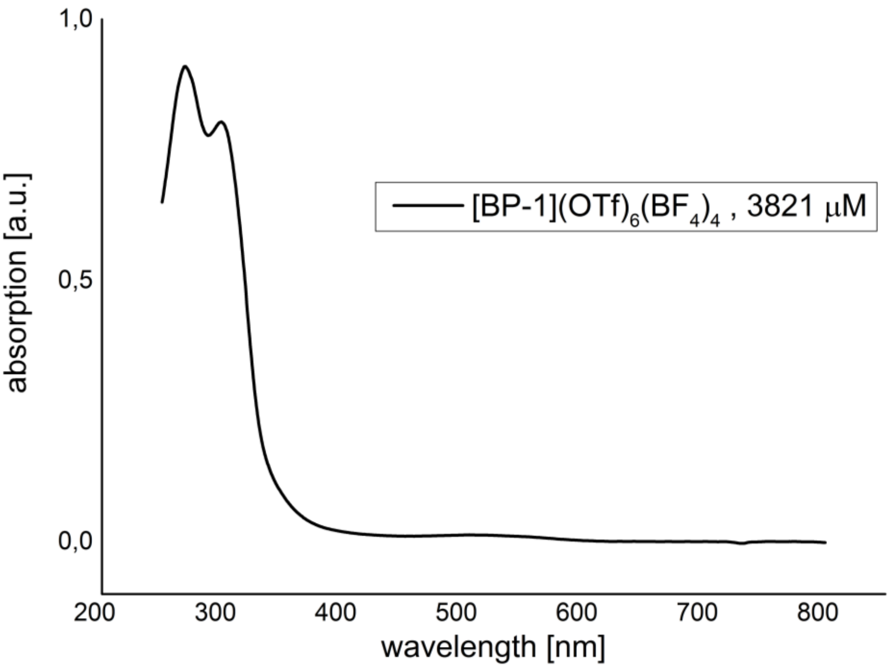

Figure S18. UV-vis spectrum of **BP-1**$(OTf)_6(BF_4)_4$ (acetonitrile, 3821 µM, 0.01 mm cuvette).

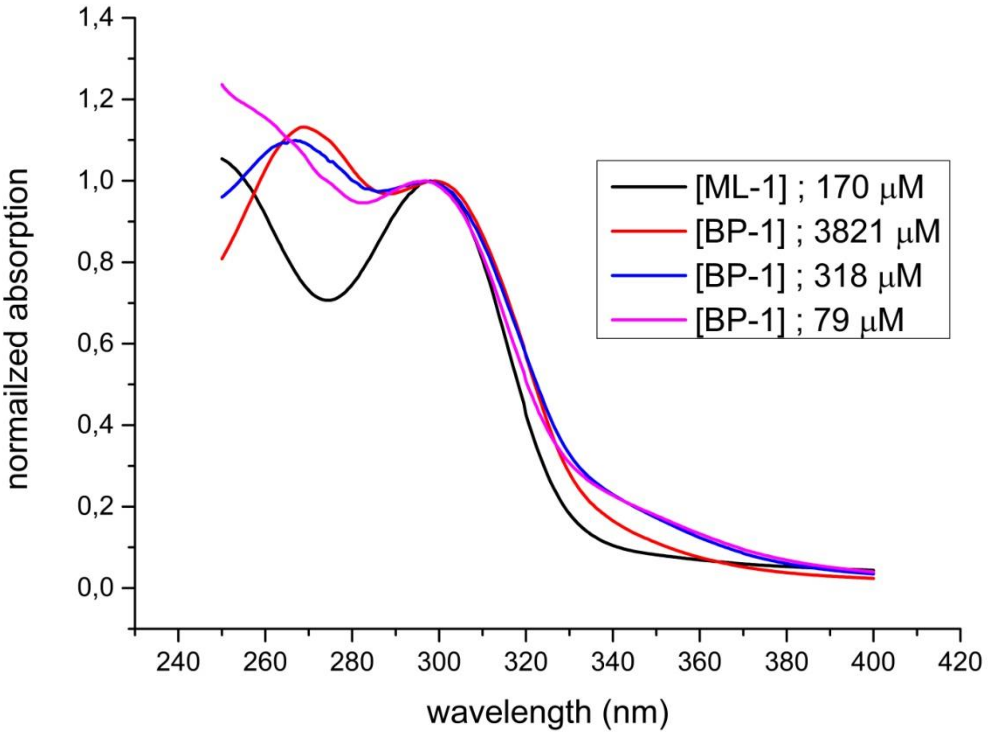

Figure S19. Superimposed and normalised UV-vis spectra (240 – 400 nm) of **ML-1**$(BF_4)_2$ and **BP-1**$(OTf)_6(BF_4)_4$, the latter with different concentrations, each in acetonitrile. Used cuvettes are: **ML-1** (170 µM) 10 mm; **BP-1** (3821 µM) 0.01 mm; **BP-1** (318 µM) 1 mm; **BP-1** (79 µM) 10 mm.

Figure S19 shows concentration dependent UV-vis spectra of **BP-1**$(OTf)_6(BF_4)_4$ in comparison with the spectrum of **ML-1**$(BF_4)_2$. While the spectra from 3821 µM and 318 µM complex solutions show a maximum at 269 nm this maximum was not found with the 79 µM solution sample. Comparison with the UV-vis spectrum of the mononuclear metalloligand indicates the

disassembly of the heterobimetallic cage into its building blocks, the metalloligand [**ML-1**]$^{2+}$ and Pd(dppp)$^{2+}$, most probably because this concentration is below the critical self-assembly concentration of the complex.

A crystalline sample of **BP-1**(OTf)$_6$(BF$_4$)$_4$ was used to determine the magnetic properties in the solid state (Figure S20). During sample preparation we observed the rapid loss of solvent after removing the crystals from the mother liquor. However, the loss of solvent was less pronounced than with **ML-1**(BF$_4$)$_2$. We observed a molar magnetic susceptibility multiplied with temperature of 6.0 cm^3 K mol^{-1} in the temperature range from 200 to 300 K, which is in very good agreement with the expected value for two uncoupled iron(II) cations in the high-spin state of X_mT = 6.002 cm^3 K mol^{-1}.[5]

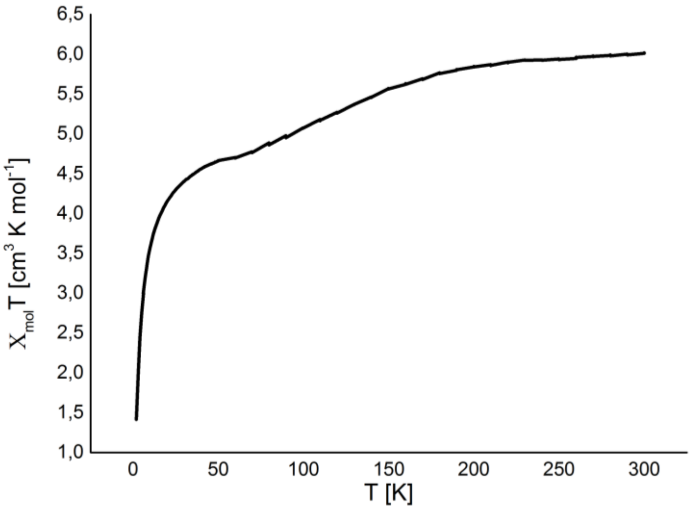

Figure S20. Temperature dependent VSM measurement (10000 Oe) of a crystalline sample of **BP-1**(OTf)$_6$(BF$_4$)$_4$.

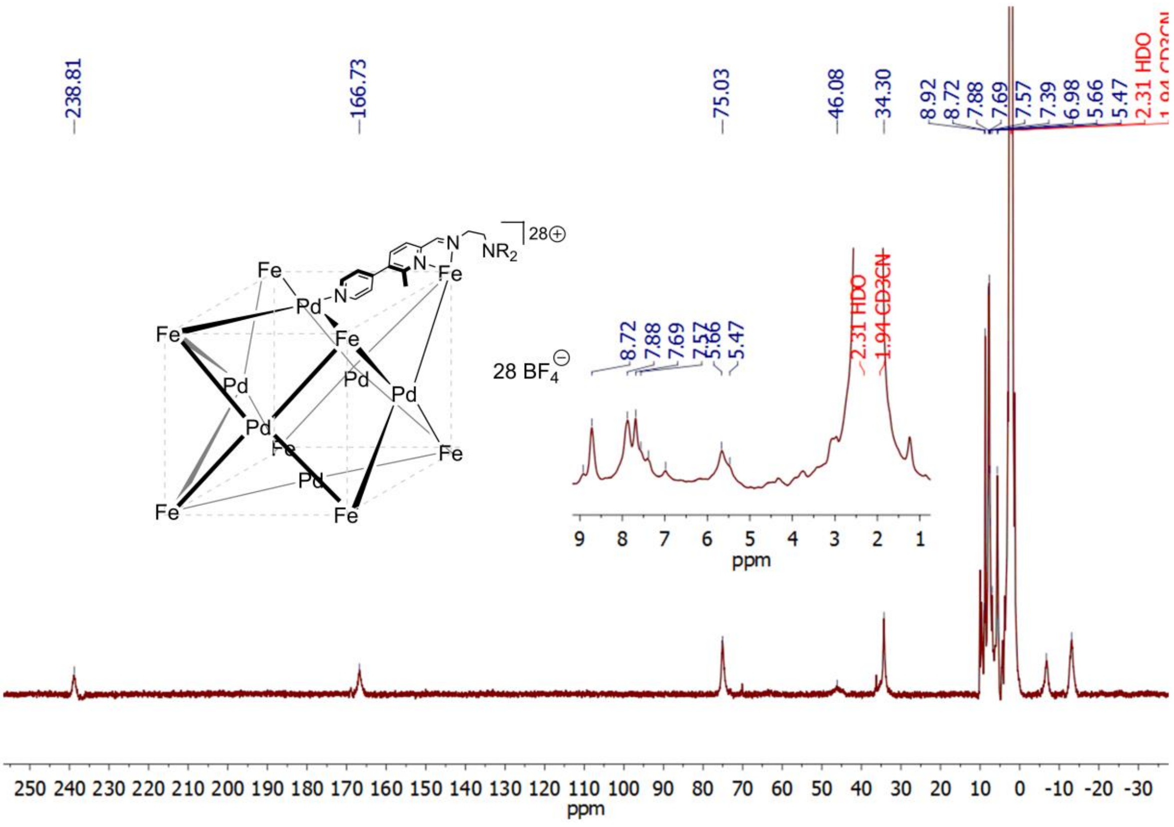

Figure S21. Paramagnetic ^{1}H-NMR spectrum (300 MHz, acetonitrile-d_3, 298 K) of **CU-1**(BF$_4$)$_{28}$.

The chemical shift of δ = 238.8 ppm is consistent with the predicted chemical shift for imine protons in paramagnetic iron(II) compounds with S = 2.[3]

The magnetic susceptibility of **CU-1**$(BF_4)_{28}$ in solution at room temperature was obtained by employing the Evans' method as described in the literature.[4] The molar magnetic susceptibility multiplied with temperature was determined to be 24.10 cm^3 K mol^{-1} (Table S5, Figure S22) and is consistent with an expected value for X_mT of eight uncoupled iron(II) cations in the high-spin state (S = 2) of 24.008 cm^3 K mol^{-1}.[5]

Table S5: Magnetic susceptibility of **CU-1**$(BF_4)_{28}$ in acetonitrile-d_3 as determined by the Evans' method.

concentration [mM]	temperature [K]	Δδ (*t*-BuOH) [Hz]	X_mT [cm^3 K mol^{-1}]
0.62	298	63.03	24.10

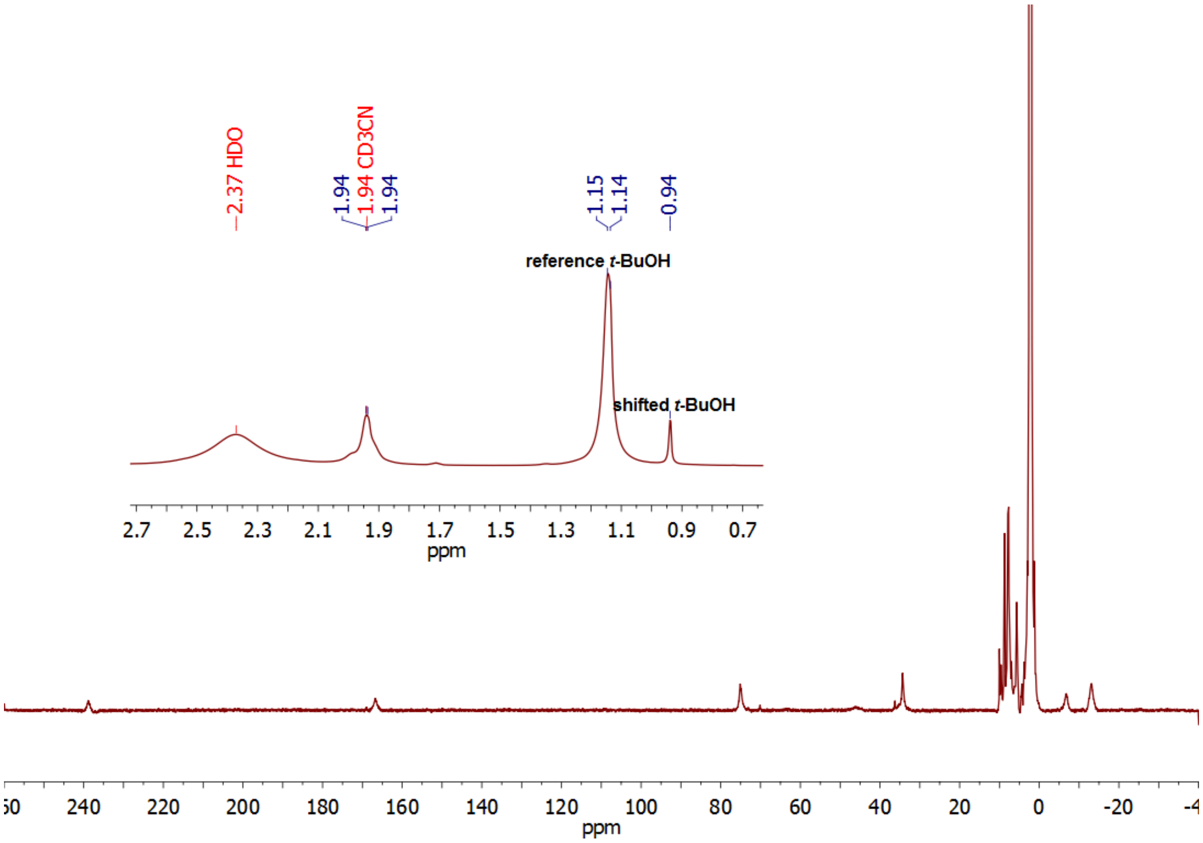

Figure S22. Evans' experiment (300 MHz, acetonitrile-d_3, 298 K) of **CU-1**$(BF_4)_{28}$.

The progress of the self-assembly to **CU-1**$(BF_4)_{28}$ was followed using NMR spectroscopy. Wide sweep ^{1}H-NMR spectra were measured after different times of heating the complex solution after the addition of $[Pd(CH_3CN)_4](BF_4)_2$ to a solution of **ML-1**$(BF_4)_2$ in acetonitrile-d_3 (Figure S23). The spectra show the impressively rapid formation of the heterobimetallic cubic cage. Already after combining all building blocks and leaving the solution at room temperature first signals assigned to the complex were detected. However, these signals are very broad and have a low intensity. After heating the solution to 50 °C for 3 hours the complex formation was finished and all signals referring to the cubic cage were detected. Further heating overnight did not lead to any changes in the ^{1}H-NMR spectra. This very fast complex formation impressively shows both, the kinetic lability of this complex, as well as the much faster self-assembly compared to the analogues diamagnetic system that took 5 days at 65 °C to assemble.[6]

[6] M. Hardy, N. Struch, F. Topić, G. Schnakenburg, K. Rissanen, A. Lützen, *Inorg. Chem.* **2018**, *57*, 3507–3515.

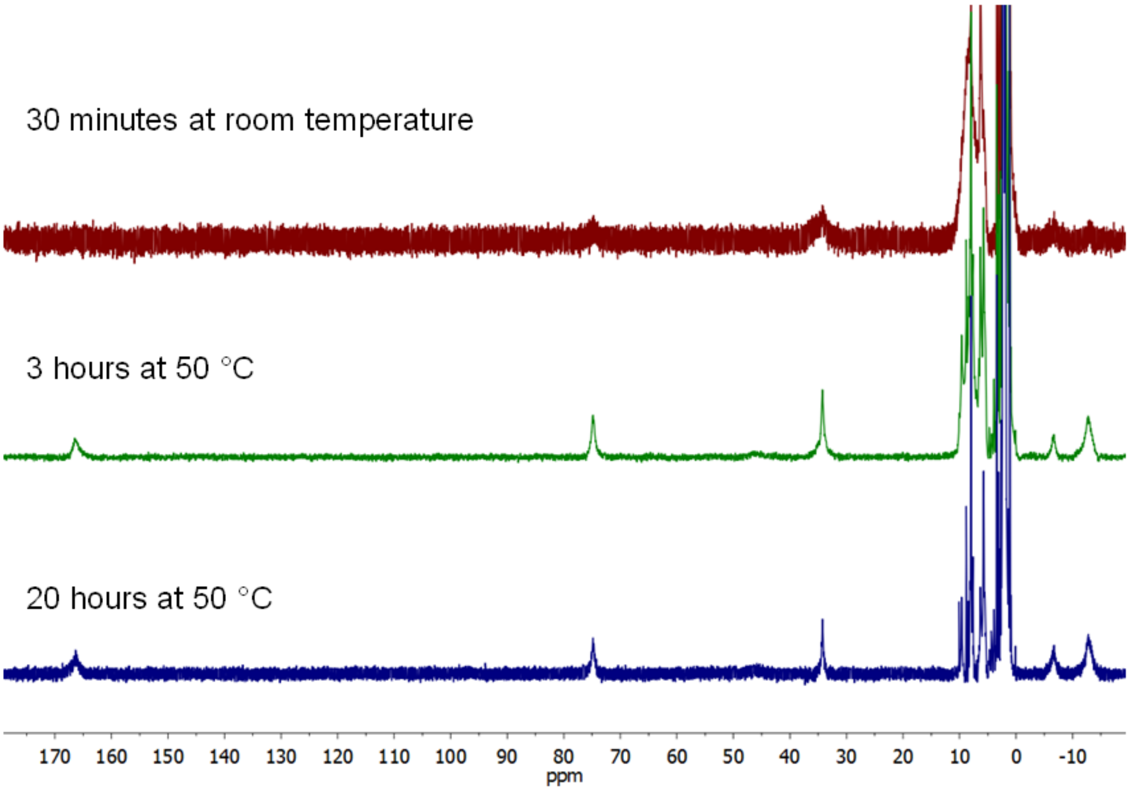

Figure S23. Paramagnetic ^{1}H-NMR spectra of **CU-1**(BF_4)$_{28}$ after different time intervals at different temperatures. Top: 30 minutes at room temperature (400 MHz, acetonitrile-d_3, 298 K), middle: 3 hours at 50 °C (500 MHz, acetonitrile-d_3, 298 K), bottom: 20 hours at 50 °C (400 MHz, acetonitrile-d_3, 298 K).

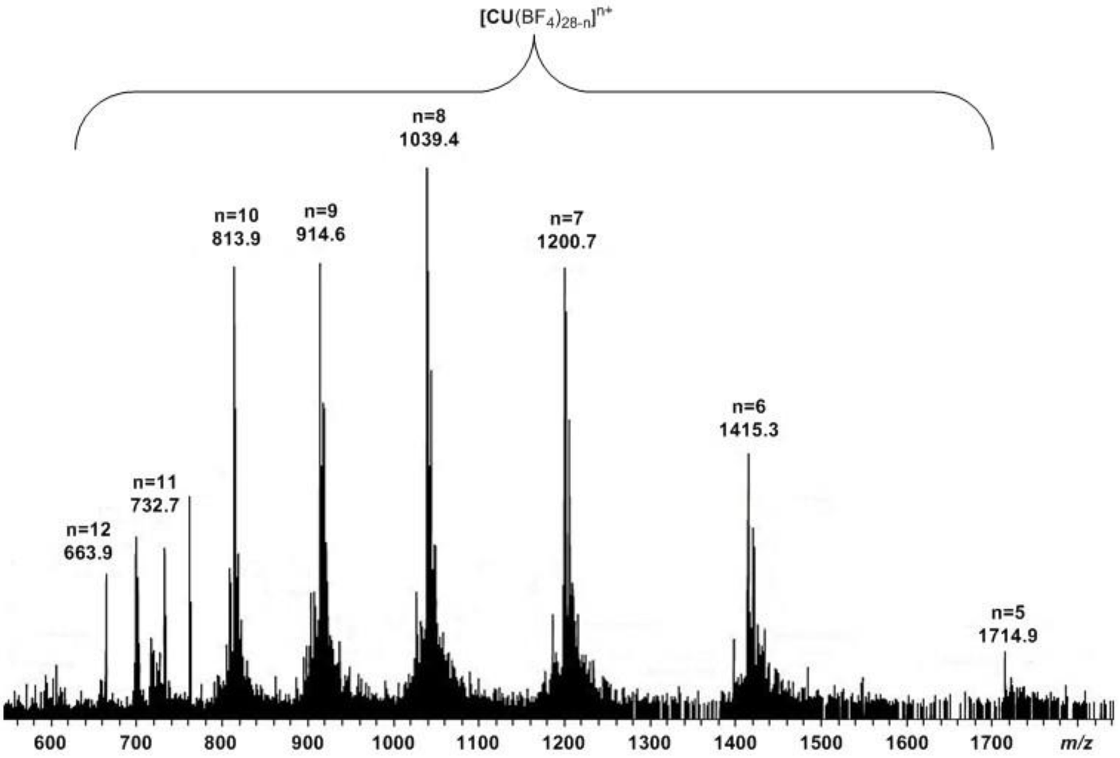

Figure S24. ESI(+)-MS spectrum (from acetonitrile) of **CU-1**(BF_4)$_{28}$.

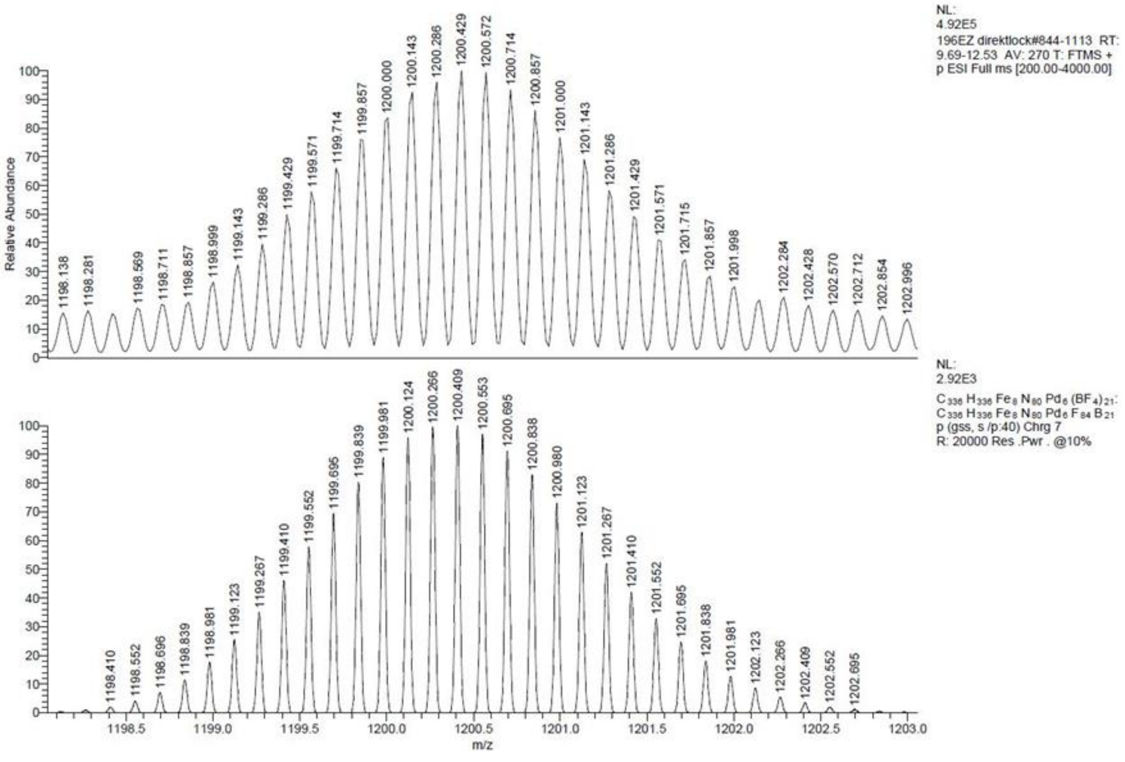

Figure S25. Exact mass of **CU-1**$(BF_4)_{21}^{7+}$. Top: Observed spectrum. Bottom: Calculated spectrum.

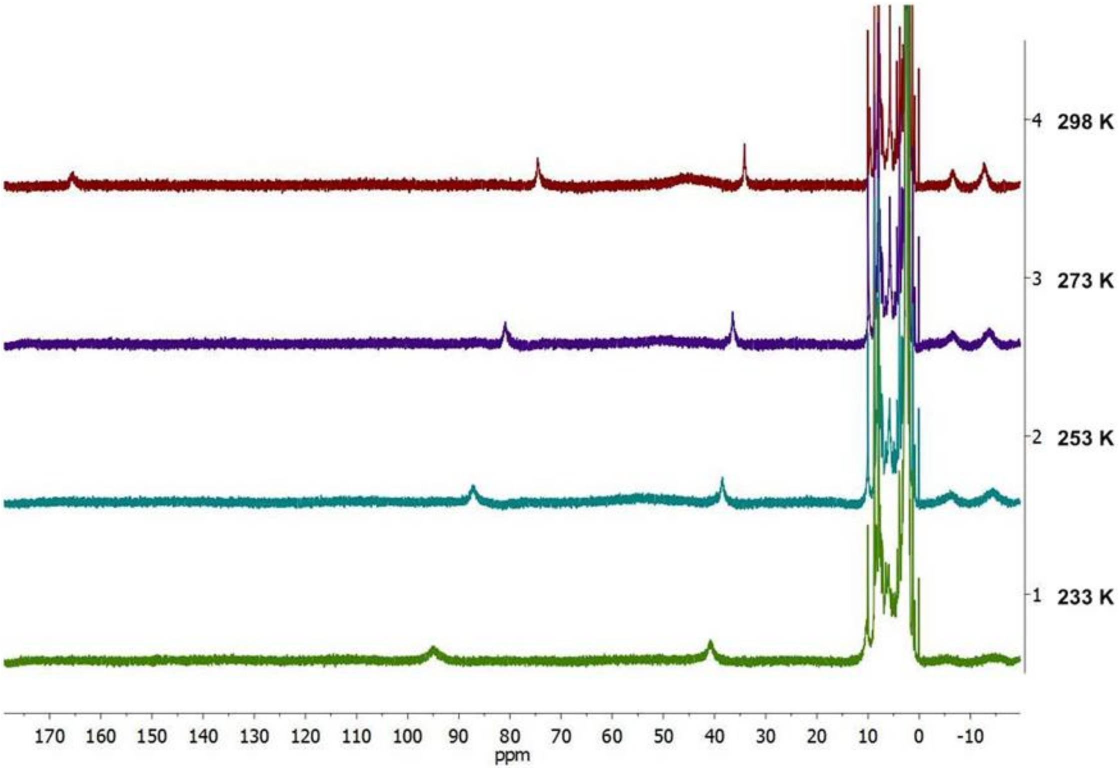

Figure S26. Temperature dependent wide-sweep ^{1}H-NMR spectra (500 MHz, acetonitrile-d_3, 298 K) of **CU-1**$(BF_4)_{28}$.

The temperature dependent ^{1}H-NMR spectra of **CU-1**$(BF_4)_{28}$ (Figure S26) show that the paramagnetic high spin state of the iron(II) cations is stabilized in the temperature range from 233 K to 298 K in solution. Analogue to the findings with **ML-1**$(BF_4)_2$ (Figure S9, Table S2) the product δT is constant, if the magnetic susceptibility does not change (Table S6).

Table S6. Comparison of δT values for one selected ^{1}H-NMR signal of **CU-1**$(BF_4)_{28}$. The observed variation of the product δT is below 1 %, proving that the paramagnetic high-spin state is stabilized in the given temperature range.

Temperature [K]	Chemical shift [ppm]	δT [ppm K]
233	95.0	22126
253	87.2	22070
273	81.0	22105
298	74.5	22201

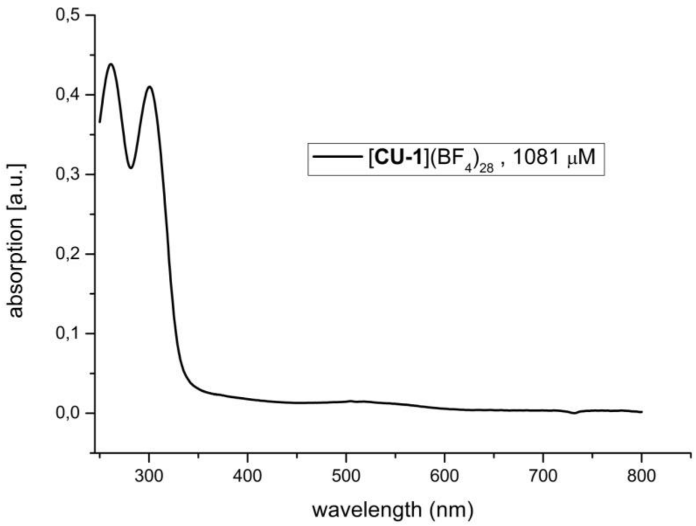

Figure S27. UV-vis spectrum of **CU-1**$(BF_4)_{28}$ (acetonitrile, 1081 µM, 0.01 mm cuvette).

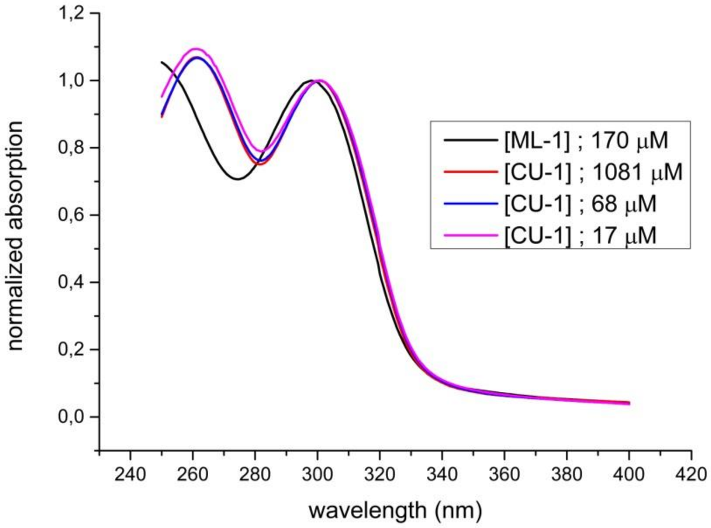

Figure S28. Superimposed and normalized UV-vis spectra (240 – 400 nm) of **ML-1**$(BF_4)_2$ and **CU-1**$(BF_4)_{28}$, the latter with different concentrations, each in acetonitrile. Used cuvettes are: **ML-1** (170 µM) 10 mm; **CU-1** (1081 µM) 0.01 mm; **CU-1** (68 µM) 1 mm; **CU-1** (17 µM) 10 mm.

The superimposed UV-vis spectra of **CU-1**$(BF_4)_{28}$ with different concentrations (Figure S28) show that the UV-vis spectrum of this heterometallic cage is independent from the complex concentration in the investigated concentration range. This indicates that the critical self-assembly concentration is below 17 µmol L^{-1} and may demonstrate the higher thermodynamic stability of **CU-1** over **BP-1**, which already decomposed at a concentration of 79 µmol L^{-1} (Figure S19). This finding might also be responsible for the more readily incorporation of the less bulky subcomponent **3** by **BP-1**$(OTf)_6(BF_4)_4$ compared to **CU-1**$(BF_4)_{28}$ (vide infra).

Crystallographic Data

Compound	**ML-1**$(BF_4)_2$	**BP-1**$(OTf)_6(BF_4)_4$	**CU-1**$(BF_4)_{28}$
CCDC number	CCDC 1945937	CCDC 1945938	CCDC 1945939
Empirical formula	$C_{90}H_{93}B_4F_{16}Fe_2N_{23}$	$C_{168}H_{161}BF_{13}Fe_2N_{20}O_9P_6Pd_3S_3$	$C_{336}H_{336}B_8F_{32}Fe_8N_{80}Pd_6$
Formula weight	1955.81	3574.87	7274.51
Temperature (K)	100(2)	100(2)	80(2)
Wavelength (Å)	MoKα (λ = 0.71073)	MoKα (λ = 0.71073)	0.6888
Crystal system	triclinic	monoclinic	cubic
Space group	P-1	C2/c	*F*m-3c
a (Å)	15.194(2)	58.121(12)	50.026(6)
b (Å)	17.666(3)	20.429(4)	50.026(6)
c (Å)	19.218(3)	43.550(9)	50.026(6)
α (º)	66.566(9)	90	90
β (º)	89.444(8)	109.799(4)	90
γ (º)	74.478(9)	90	90
Volume ($Å^3$)	4532.9(13)	48653(17)	125195(44)
Z	2	8	8
Density (calc.) (Mg/m^3)	1.433	0.976	0.772
Absorption coefficient (mm^{-1})	0.414	0.452	0.358
F(000)	2020.0	14648.0	29792
Crystal size (mm^3)	0.26 × 0.12 × 0.06	0.32 × 0.08 × 0.04	0.050 x 0.050 x 0.020
θ range for data collection (º)	4.648 to 52	4.224 to 50.054	0.789 to 17.291
Reflections collected	65533	170574	61976
Independent reflections [R(int)]	17434 [0.3014]	42121 [0.1085]	1857 [0.0806]
Data / restraints / parameters	17434/498/1272	42121/75/2020	1857 / 329 / 178
Goodness-of-fit on F^2	1.043	1.037	1.632
R_1 [I>2σ(I)]	0.1722	0.0775	0.1139
wR_2 (all data)	0.4097	0.2313	0.3824
Largest diff. peak and hole (e.$Å^{-3}$)	1.27/-1.41	1.45/-0.82	1.536 and -0.348

Crystal structure of ML-1(BF$_4$)$_2$

The slow diffusion of diethyl ether vapor into a concentrated solution of **ML-1**(BF$_4$)$_2$ in acetonitrile gave X-ray quality single crystals, suitable to determine the solid state structure of the metalloligand as the tetrafluoroborate salt (figure S29 and figure S30). The mononuclear complex crystallizes in the achiral triclinic space group P-1. The coordination sphere around the iron cation is best described as octahedral and arises from six nitrogen atoms of the *tris*(pyridylimin) binding site with an average Fe-N bond length of 2.168 Å, being consistent with a paramagnetic high-spin iron(II) complex.[7] As dictated by the ligand system only the *fac*-isomer of **ML-1**(BF$_4$)$_2$ is observed.

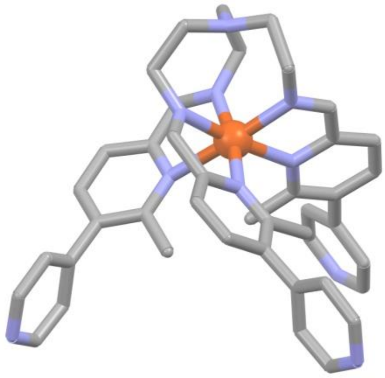

Figure S29. Solid state structure of **ML-1**(BF$_4$)$_2$ as determined by single crystal X-ray diffraction. Hydrogen atoms, solvent molecules and counter anions are omitted for clarity.

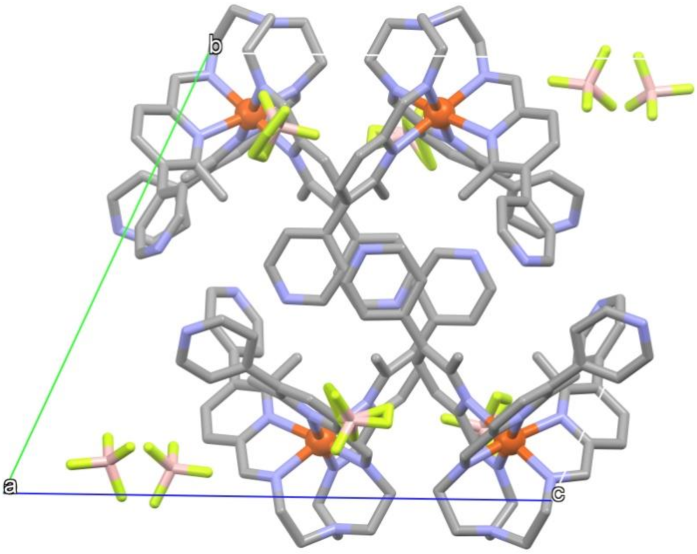

Figure S30. Crystallographic unit cell of **ML-1**(BF$_4$)$_2$ with (Δ)- and (Λ)-enantiomers.

[7] P. Gütlich, A. Hauser, H. Spiering, *Angew. Chem. Int. Ed. Engl.* **1994**, *33*, 2024 – 2054; *Angew. Chem.* **1994**, *106*, 2109–2141.

Crystal structure of BP-1(OTf)$_6$(BF$_4$)$_4$

Very slow diffusion of diethyl ether into a concentrated solution of BP-1(OTf)$_6$(BF$_4$)$_4$ in acetonitrile yielded clear red crystals, suitable to determine the solid structure of the heterobimetallic cage.

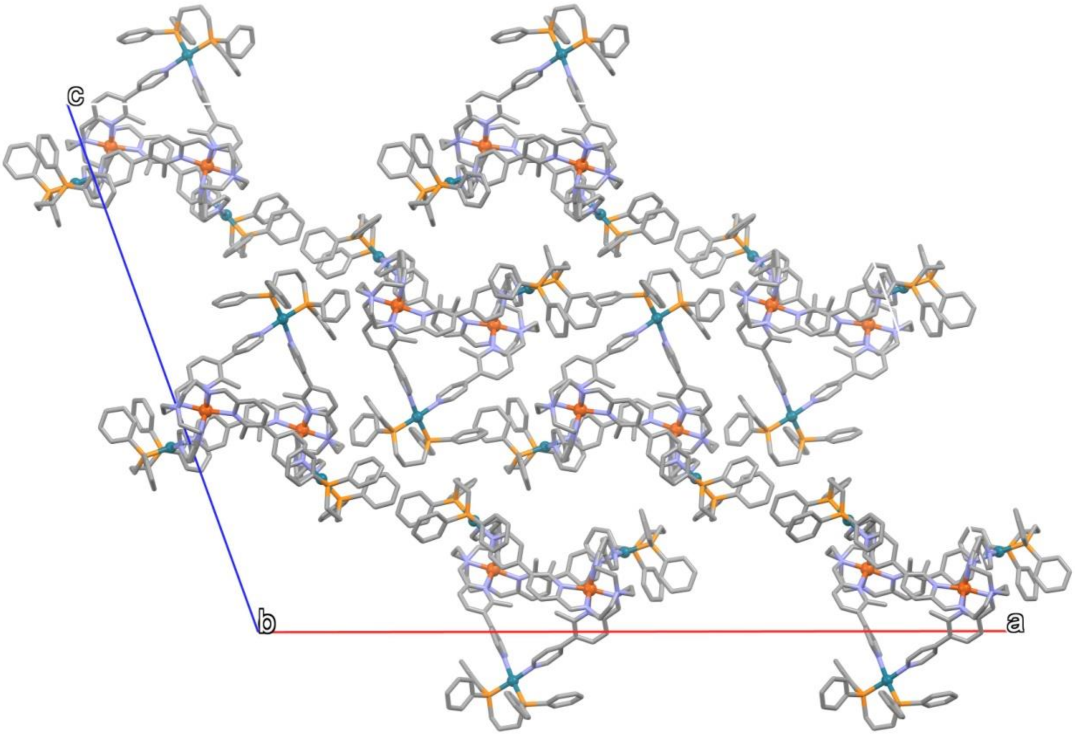

Figure S31. Crystallographic unit cell of **BP-1**(OTf)$_6$(BF$_4$)$_4$ with (Δ,Δ)- and (Λ,Λ)-enantiomers. Hydrogen atoms, solvent molecules and counter anions are omitted for clarity.

The data collections of **ML-1**(BF$_4$)$_2$ and **BP-1**(OTf)$_6$(BF$_4$)$_4$ were performed on a Bruker D8-Venture diffractometer by using multi-layer mirror optics monochromated Mo-K$_\alpha$ radiation (λ = 0.71073 Å). The diffractometer was equipped with a low-temperature device (Cryostream 800er series, 100(2) K). Intensities were measured by fine-slicing φ- and ω-scans and corrected for background, polarization and Lorentz effects. Semi-empirical absorption corrections were applied for all data sets following Blessing's method.[S8] The structures were solved by direct methods and refined anisotropically by the least-squares procedure implemented in the ShelX program system.[S9] The hydrogen atoms were included isotropically using the riding model on the bound carbon atoms. The contribution of the electron density from disordered counterions in **BP-1**(OTf)$_6$(BF$_4$)$_4$, which could not be modelled with discrete atomic positions were handled using the SQUEEZE[10] routine in PLATON.[11] The solvent mask file (.fab) computed by PLATON were included in the SHELXL refinement via the ABIN instruction leaving the measured intensities untouched.

8 R. H. Blessing, *Acta Cryst. A* **1995**, *51,* 33.
9 G. M. Sheldrick*, Acta Cryst. A* **2015**, *71*, 3-8.
10 A. Spek, *Acta Crystallogr. Sect. C* **2015**, *71*, 9–18.
11 A. Spek, *Acta Crystallogr. Sect. D* **2009**, *65*, 148–155.

Crystal structure of CU-1(BF_4)$_{28}$

Single crystals suitable for X-ray diffraction were obtained by very slow evaporation of solvent from an acetonitrile solution of **CU-1**(BF_4)$_{28}$ over 3 months. Single crystals of **CU-1**(BF_4)$_{28}$ was transferred onto a glass slide covered by NVH oil. Seven crystals were quickly mounted onto 40 to 200 µm nylon loops and immediately flash cooled in liquid nitrogen to avoid collapse of the crystal lattice.
Crystals were stored at cryogenic temperature in dry shippers, in which they were safely transported to macromolecular beamline P11,[12] Petra III, DESY, Hamburg, Germany. Samples were mounted using the StäubliTX60L robotic arm. A wavelength of λ = 0.6889 Å was chosen using a liquid N_2 cooled double crystal monochromator. Single crystal X-ray diffraction data was collected at 80(2) K on a single axis goniometer, equipped with an Oxford Cryostream 800 and a Pilatus 6M fast. 1800 diffraction images were collected in a 360° φ sweep at a detector distance of 250 mm, 100% filter transmission, 0.2° step width and 0.2 second exposure time per image. Due to radiation damage indicated by drastically reduced resolution in the second half of the collection, only the first 900 frames were used in final integration with XDS.[13]
The data was cut at 1.18 Å, as the signal to noise ratio has dropped below $I/\sigma(I) < 2.0$. Due to high mosaicity and disorder in the solvent region a higher resolution could not be achieved. Nevertheless the resolution achieved was sufficient to solve the structure by intrinsic phasing/direct methods using SHELXT[14]. SHELXL[15] (version 2014/7) was used for refinement and ShelXle[16] as a graphical user interface. The DSR program plugin was employed for modelling.[17-18] All cycles were refined against F^2 until convergence using the conjugate-gradient algorithm (CGLS). Only for computing the crystallographic information file (CIF) the full-matrix least-squares routine was employed. Hydrogen atoms were included as invariants at geometrically estimated.

Specific refinement details

In order to generate a molecular model and increase robustness of the refinement we have adapted and exploited techniques commonly applied in macromolecular structure refinement. The obtained geometry from the **ML-1**(BF_4)$_2$ structure was used as input for stereochemical restraints generation by the GRADE program using the GRADE Web Server (http://grade.globalphasing.org). The restraint dictionary was applied to the ligand in the refinement using residue name MPY. A GRADE dictionary for SHELXL contains target values and standard deviations for 1.2-distances (DFIX) and 1.3-distances (DANG), as well as restraints for planar groups (FLAT). The refinement of ADP's for non-hydrogen atoms was enabled by using the rigid bond restraint (RIGU)[19] in the SHELXL program in combination with SIMU restraints. The contribution of the electron density from disordered counterions, and solvent molecules, which could not be modelled with discrete atomic positions were handled using the SQUEEZE[7] routine in PLATON.[8] The solvent mask file (.fab) computed by PLATON were included in the SHELXL refinement via the ABIN instruction leaving the measured intensities untouched.

[12] A. Burkhardt, T. Pakendorf, B. Reime, J. Meyer, P. Fischer, N. Stübe, S. Panneerselvam, O. Lorbeer, K. Stachnik, M. Warmer, P. Rödig, D. Göries and A. Meents, *Eur. Phys. J. Plus* **2016**, *131*, 56
[13] W. Kabsch, *Acta Crystallogr. Sect. D* **2010**, *66*, 125–132.
[14] G. M. Sheldrick, *Acta Crystallogr. Sect. A* **2015**, *71*, 3–8.
[15] G. M. Sheldrick, *Acta Crystallogr. Sect. C* **2015**, *71*, 3–8.
[16] C. B. Hubschle, G. M. Sheldrick and B. Dittrich, *J. Appl. Cryst.* **2011**, *44*, 1281–1284.
[17] D. Kratzert, J. J. Holstein and I. Krossing, *J. Appl. Cryst.* **2015**, *48*, 933–938.
[18] D. Kratzert, I. Krossing, *J. Appl. Cryst.* **2018**, *51*, 928-934.
[19] A. Thorn, B. Dittrich and G. M. Sheldrick, *Acta Crystallogr. Sect. A* **2012**, *68*, 448–451.

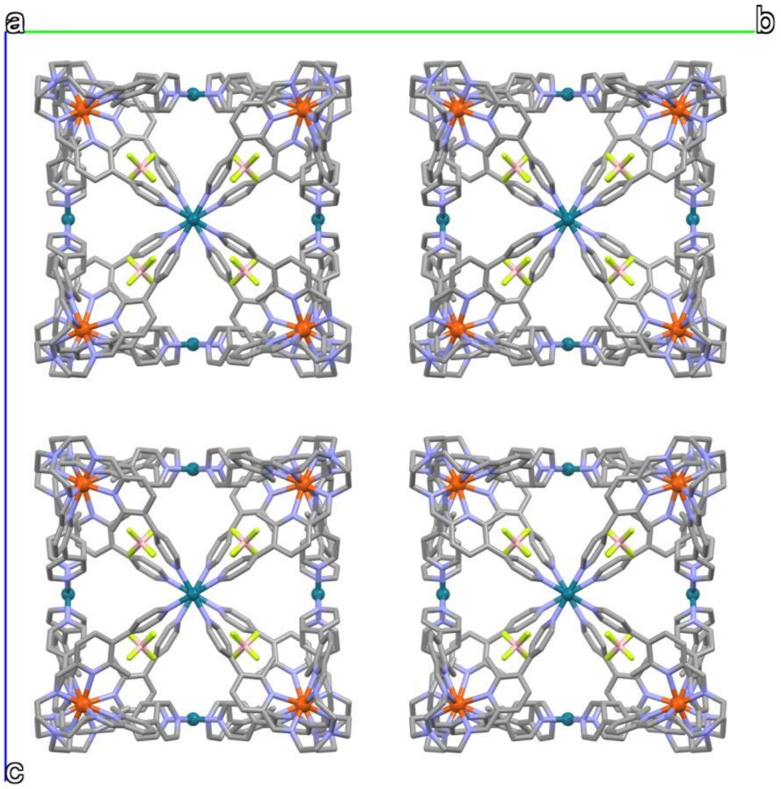

Figure S32. Crystallographic unit cell of **CU-1**(BF_4)$_{28}$ with (all-Δ)- and (all-Λ)-enantiomers. View along the crystallographic a-axis. Hydrogen atoms, solvent molecules and disordered counter anions are omitted for clarity.

Complex-to-Complex Transformations

ML-1(BF_4)$_2$ to **ML-2**(BF_4)$_2$

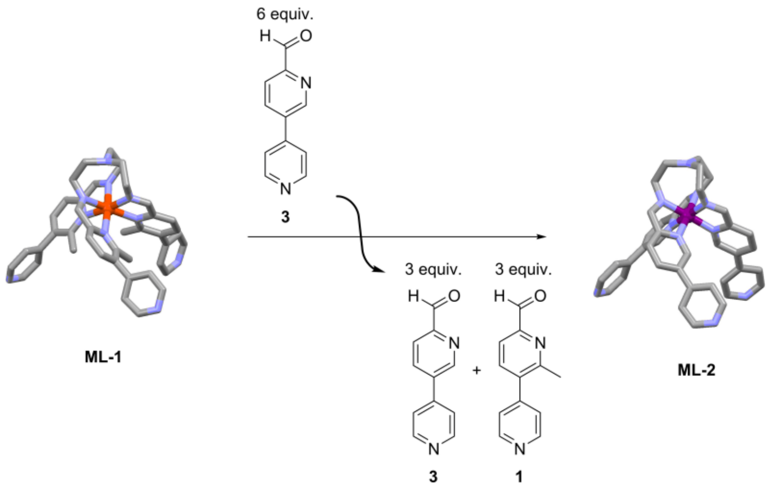

Scheme S2. Complex-to-complex transformation from **ML-1** to **ML-2** using 6 equiv of **3**.

ML-1(BF_4)$_2$ (10.00 mg, 10.90 µmol, 1.00 equiv) together with 2-formyl-5-(4'-pyridyl) pyridine **3** (12.06 mg, 65.50 µmol, 6.00 equiv) was dissolved in 1 mL acetonitrile-d_3. The solution was degassed by applying a vacuum and flushing with argon three times. The reaction was indicated by a change of colour from bright red to dark purple only few minutes after starting the reaction. To reach equilibrium the reaction mixture was heated to 40 °C for 16 hours. Then, a ^{1}H-NMR spectrum was measured to evaluate the reaction progress without any work up. In order to isolate **ML-2**(BF_4)$_2$ the reaction mixture was added drop wise to 25 mL of diethyl ether. The resulting suspension was stirred for 30 minutes at room temperature and then the purple precipitant was collected and washed with generous amounts of diethyl ether. Drying in a stream of air gave **ML-2**(BF_4)$_2$ as a purple solid in 99% yield (9.48 mg, 10.8 µmol).

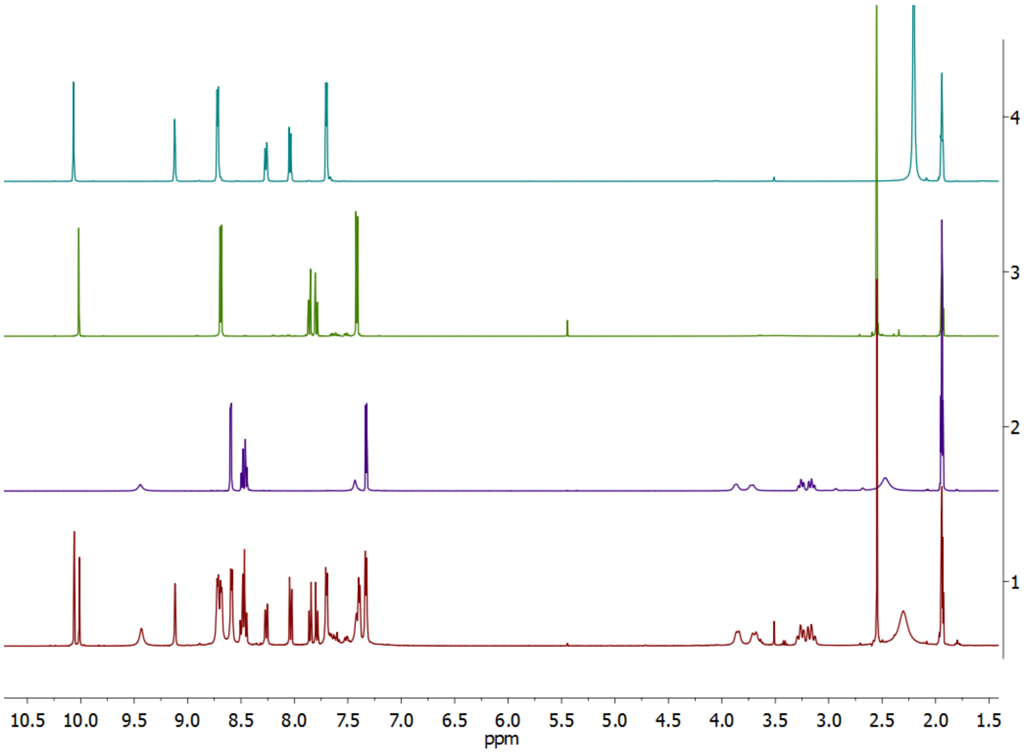

Figure S33. Stacked ^{1}H-NMR spectra of: [1] (400 MHz, acetonitrile-d_3, 298 K) the complex-to-complex transformation from **ML-1** to **ML-2**[6] (Scheme S2); [2] (500 MHz, acetonitrile-d_3, 298 K) **ML-2**(BF_4)$_2$; [3] (500 MHz, acetonitrile-d_3, 298 K) **1**; [4] (400 MHz, acetonitrile-d_3, 298 K) **3**.

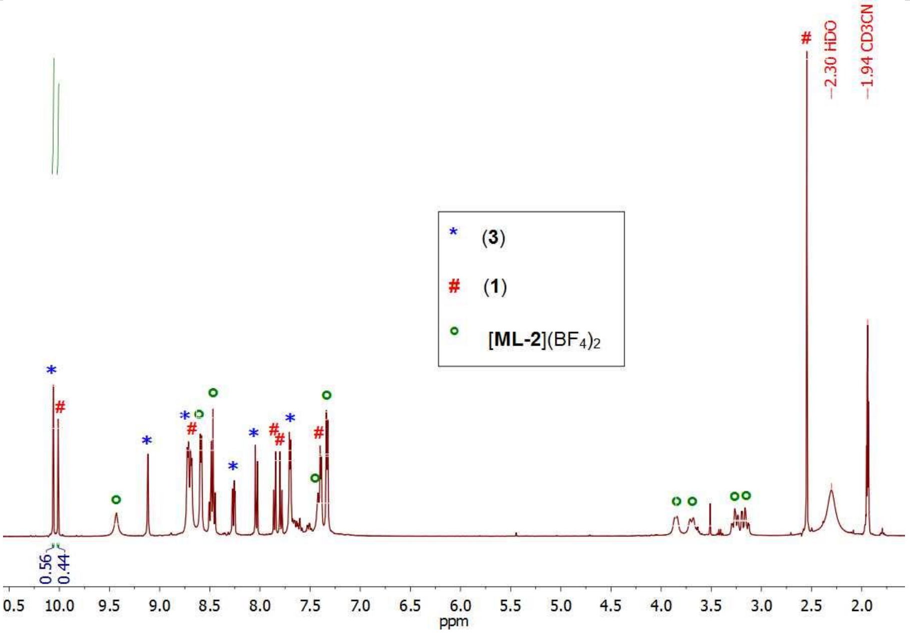

Figure S34. ^{1}H-NMR (400 MHz, acetonitrile-d_3, 298 K) of the complex-to-complex transformation from **ML-1** to **ML-2**[6] (Scheme S2). The corresponding aldehyde signals of **1** and **3** show an equilibrium ratio of 44:56.

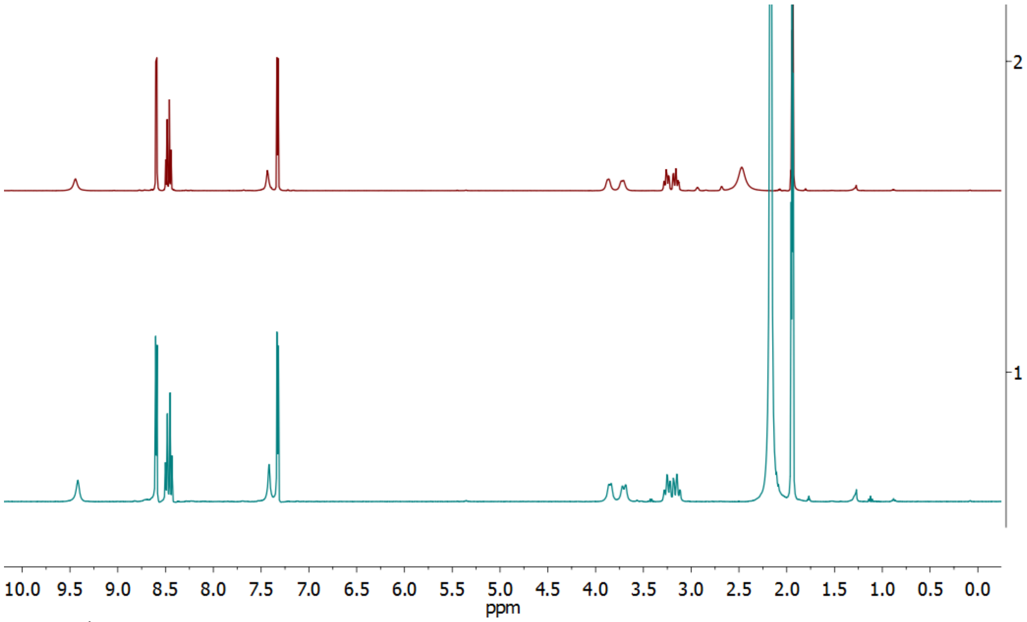

Figure S35. Stacked ^{1}H-NMR spectra of: top: **ML-2**$(BF_4)_2$ (500 MHz, acetonitrile-d_3, 298 K); bottom: isolated **ML-2**$(BF_4)_2$ from the complex-to-complex transformation from **ML-1** to **ML-2** (Scheme S2).

Comparison of the ^{1}H-NMR spectra of **ML-2**$(BF_4)_2$ and the spectrum received from the isolated complex of the complex-to-complex transformation according to scheme 2 indicates the successful formation of **ML-2**$(BF_4)_2$ (Figure S35).
An Evans' experiment with the isolated complex also revealed, that **ML-2**$(BF_4)_2$ from the complex-to-complex transformation shows no more magnetic moment, since the same signal for *t*-BuOH from the internal and external reference was found (Figure S36).

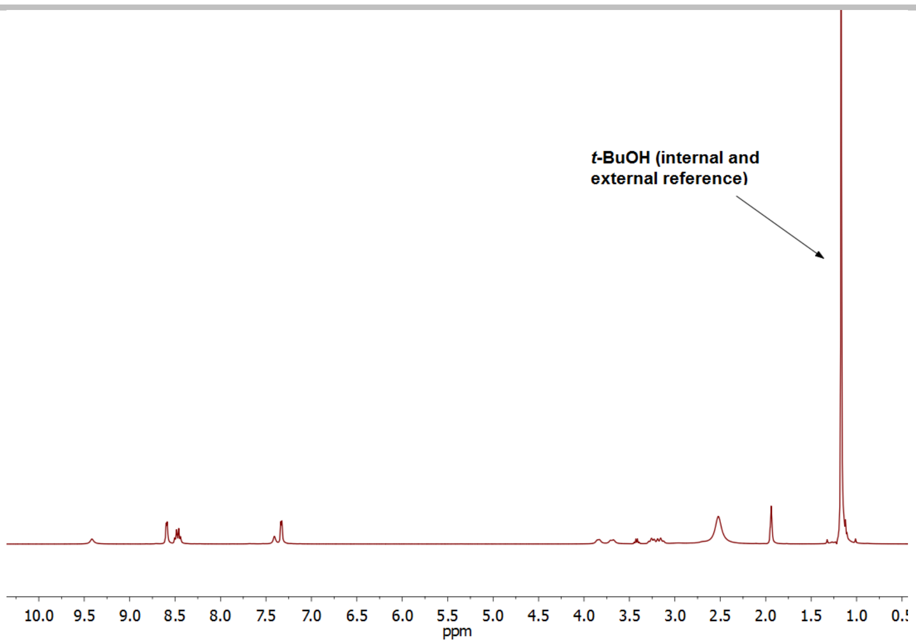

Figure S36. ^{1}H-NMR spectrum (400 MHz, acetonitrile-d_3, 298 K) of the Evans' experiment with isolated **ML-2**$(BF_4)_2$ from the complex-to-complex transformation from **ML-1** to **ML-2** (Scheme S2).

This complex-to-complex transformation is indicated by a significant change in colour. Starting from the bright red **ML-1**$(BF_4)_2$ complex solution, the transformation yields a dark purple solution of the analogues diamagnetic complex **ML-2**$(BF_4)_2$ (Figure S37).

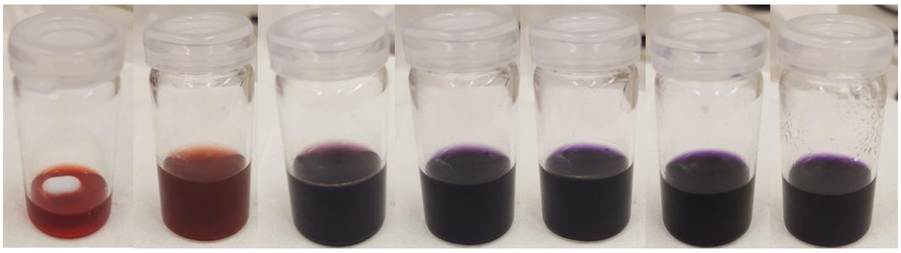

Figure S37. Colour change during the complex-to-complex transformation of **ML-1**$(BF_4)_2$ to **ML-2**$(BF_4)_2$. The reaction was carried out with 6 equivalents of aldehyde **3** at room temperature and the pictures show the reaction progress over 3 hours.

The reaction progress can be tracked by measuring a UV-vis spectrum of the reaction mixture every 15 minutes (Figure S38). The increasing signal at 580 nm can be assigned to the $^1A_1 \rightarrow {}^1T_1$ transition of iron(II) cations[20] of the diamagnetic metalloligand **ML-2**$(BF_4)_2$ and therefore can be used to show the reaction progress.

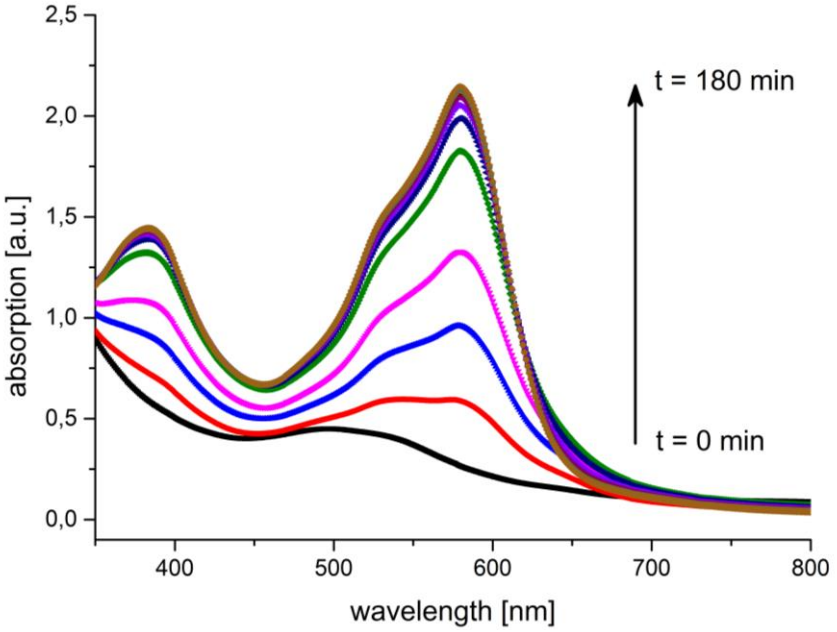

Figure S38. UV-vis spectra (acetonitrile, 1 mm cuvette, room temperature, c(**ML-2**$(BF_4)_2$) = 2.91 mmol/L) of the complex-to-complex transformation of **ML-1**$(BF_4)_2$ to **ML-2**$(BF_4)_2$ taken every 15 minutes over a period of 180 minutes. The increasing signal located at 580 nm can be assigned to the $^1A_1 \rightarrow {}^1T_1$ transition of iron(II) cations of the diamagnetic metalloligand **ML-2**$(BF_4)_2$.

[20] S. Sugano, Y. Tanabe, H. Kamimura, *Pure and Applied Physics*, *Vol. 33*, Academic Press: New York, **1970**.

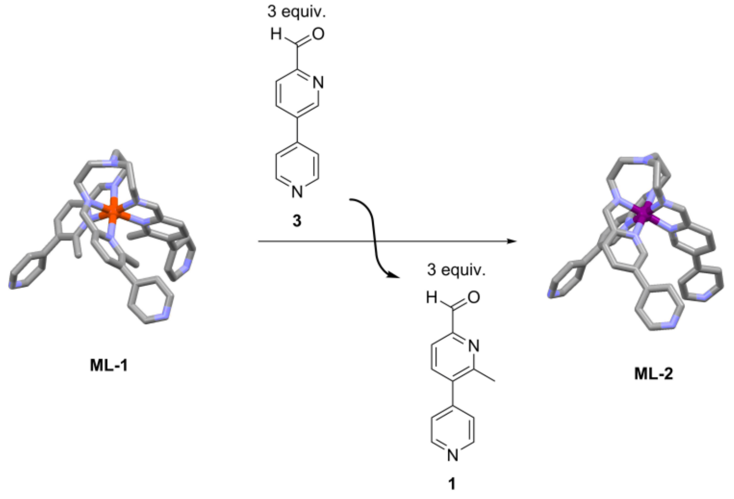

Scheme S3. Complex-to-complex transformation from **ML-1** to **ML-2** using 3 equiv of **3**.

ML-1$(BF_4)_2$ (5.00 mg, 5.46 µmol, 1.00 equiv) together with 2-formyl-5-(4'-pyridyl) pyridine **3** (3.03 mg, 16.40 µmol, 3.00 equiv) was dissolved in 0.7 mL acetonitrile-*d*$_3$. The solution was degassed by applying a vacuum and flushing with argon three times. The reaction was indicated by a change of colour from bright red to dark purple only few minutes after starting the reaction. To reach equilibrium the reaction mixture was heated to 40 °C for 36 hours. Then, a ^{1}H-NMR spectrum was measured to evaluate the reaction progress without any work up. In order to isolate **ML-2**$(BF_4)_2$ the reaction mixture was added drop wise to 25 mL of diethyl ether. The resulting suspension was stirred for 30 minutes at room temperature and then the purple precipitant was collected and washed with generous amounts of diethyl ether. Drying in a stream of air gave **ML-2**$(BF_4)_2$ as a purple solid in 95% yield (4.53 mg, 5.18 µmol).

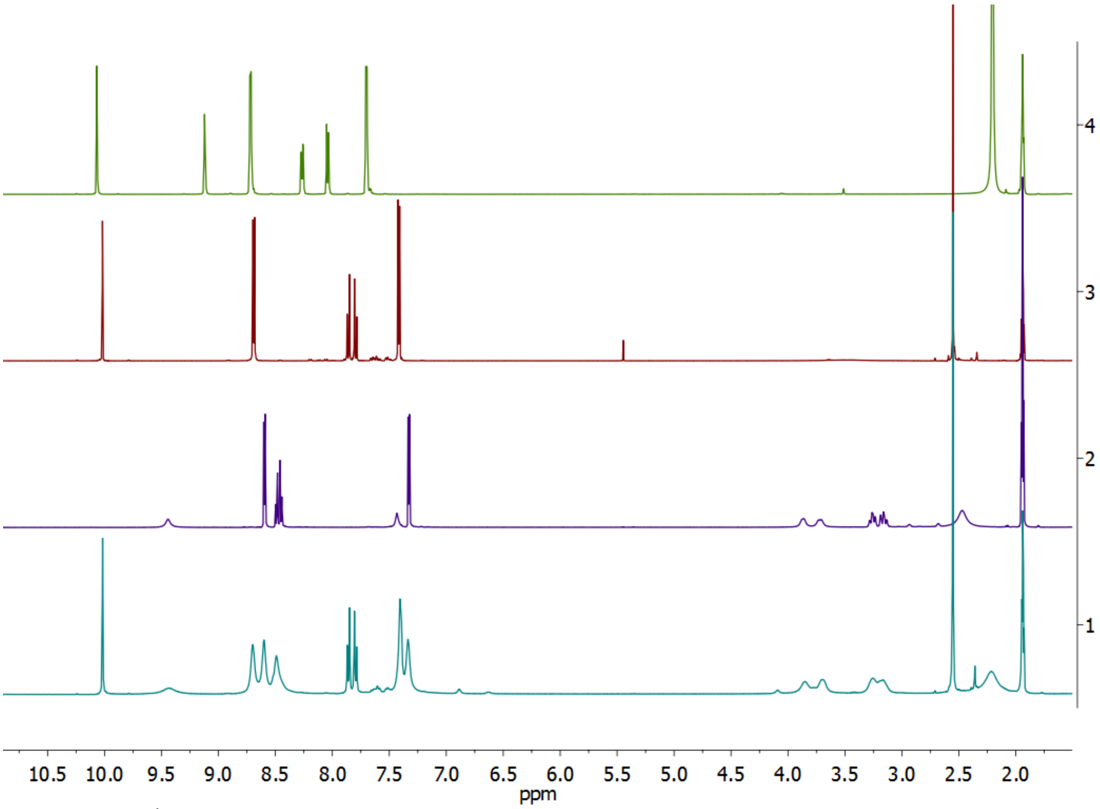

Figure S39. Stacked ^{1}H-NMR spectra of: [1] (400 MHz, acetonitrile-*d*$_3$, 298 K) the complex-to-complex transformation from **ML-1** to **ML-2**[6] (Scheme S3); [2] (500 MHz, acetonitrile-*d*$_3$, 298 K) **ML-2**$(BF_4)_2$; [3] (500 MHz, acetonitrile-*d*$_3$, 298 K) **1**; [4] (400 MHz, acetonitrile-*d*$_3$, 298 K) **3**.

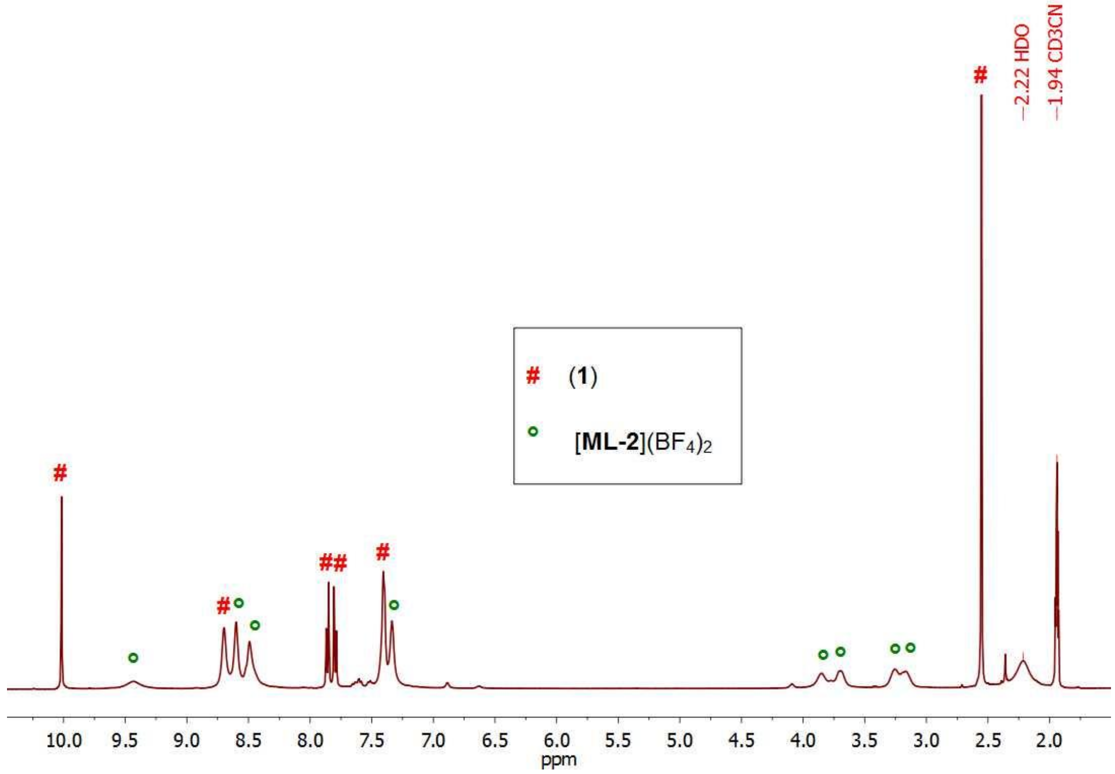

Figure S40. ^{1}H-NMR (400 MHz, acetonitrile-d_3, 298 K) of the complex-to-complex transformation from **ML-1** to **ML-2**[6] (Scheme S3). Missing signals of **3** indicate the quantitative transformation.

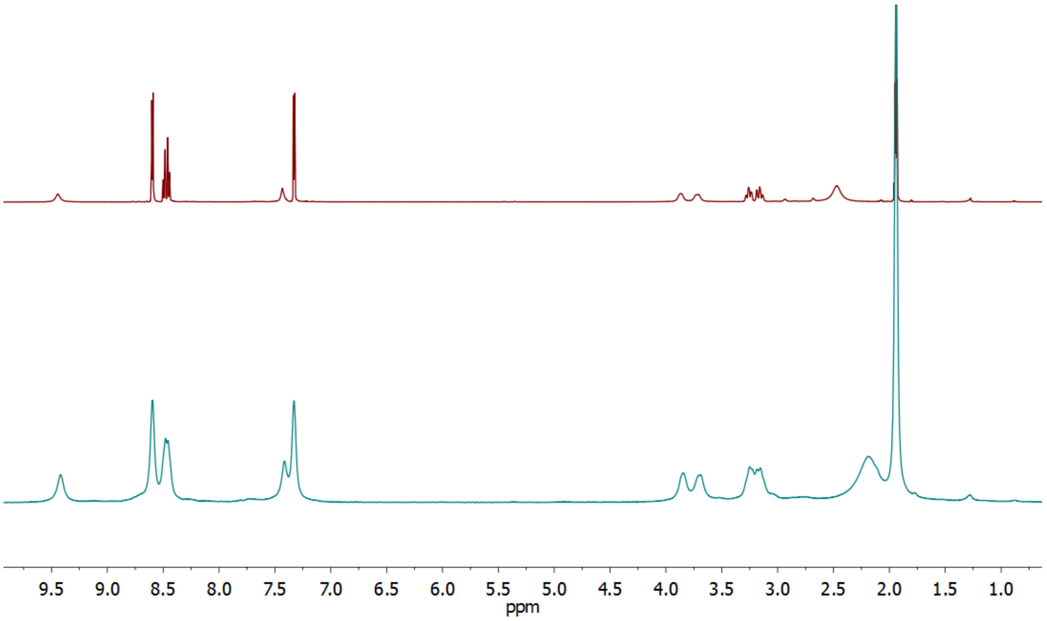

Figure S41. Stacked ^{1}H-NMR spectra of: top: **ML-2**$(BF_4)_2$ (500 MHz, acetonitrile-d_3, 298 K); bottom: isolated **ML-2**$(BF_4)_2$ (400 MHz, acetonitrile-d_3, 298 K) from the complex-to-complex transformation from **ML-1** to **ML-2** (Scheme S3).

Comparison of the ^{1}H-NMR spectra of **ML-2**$(BF_4)_2$ and the spectrum received from the isolated complex of the complex-to-complex transformation according to Scheme 3 indicates the successful formation of **ML-2**$(BF_4)_2$ (Figure S41).
An Evans' experiment with the isolated complex also revealed, that **ML-2**$(BF_4)_2$ from the complex-to-complex transformation shows no more magnetic moment, since the same signal for *t*-BuOH from the internal and external reference was found (Figure S42).

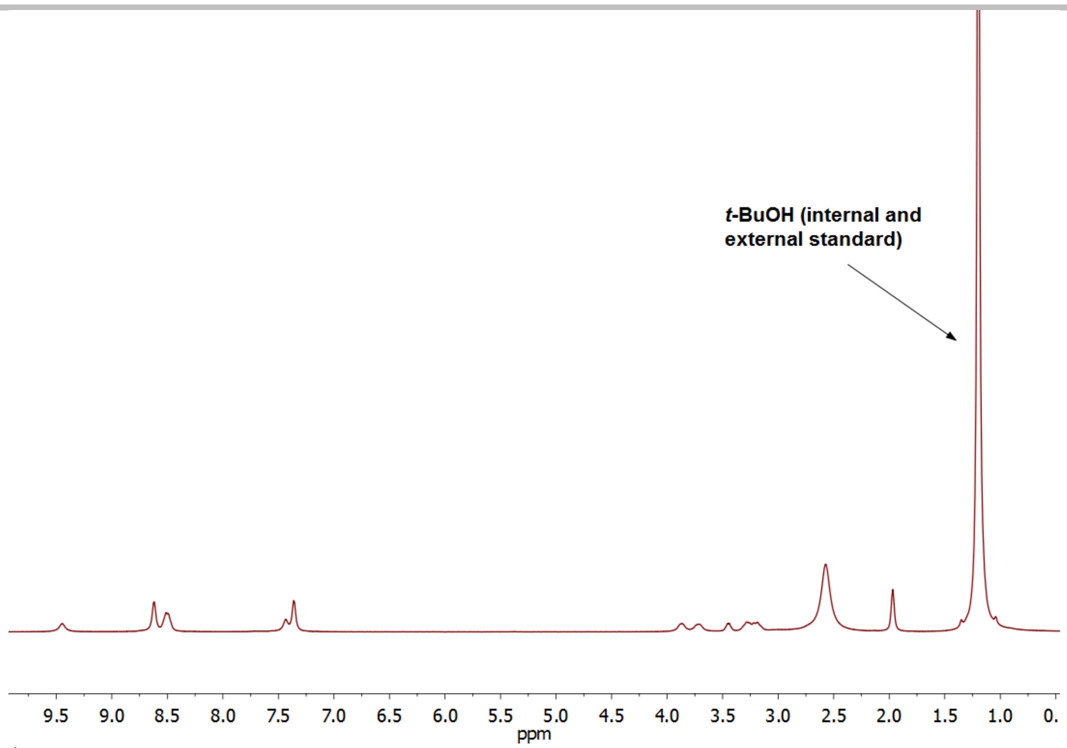

Figure S42. ^{1}H-NMR spectrum (400 MHz, acetonitrile-d_3, 298 K) of the Evans' experiment with isolated **ML-2**$(BF_4)_2$ from the complex-to-complex transformation from **ML-1** to **ML-2** (Scheme S3).

BP-1$(OTf)_6(BF_4)_4$ to **BP-2**$(OTf)_6(BF_4)_4$

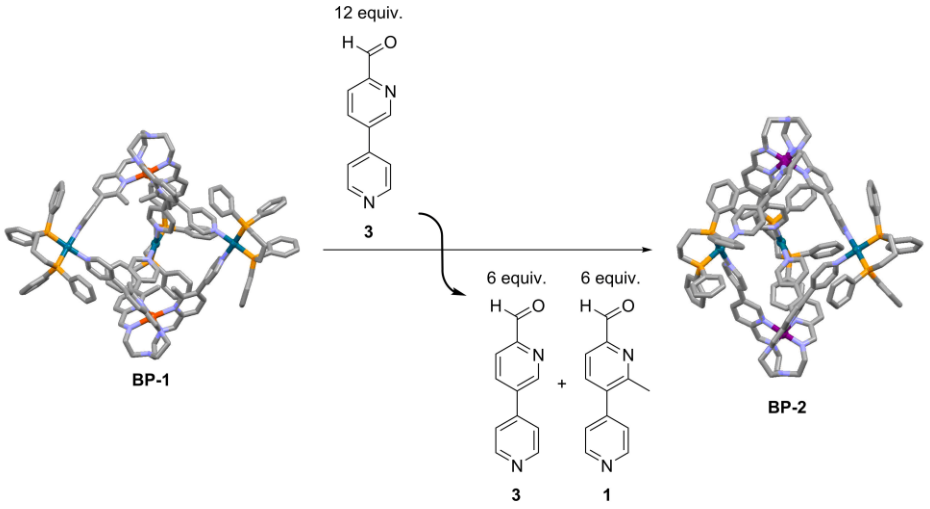

Scheme S4. Complex-to-complex transformation from **BP-1** to **BP-2** using 12 equiv of **3**.

BP-1$(OTf)_6(BF_4)_4$ (11.69 mg, 2.73 µmol, 1.00 equiv) together with 2-formyl-5-(4'-pyridyl) pyridine **3** (6.03 mg, 32.70 µmol, 12.00 equiv) was dissolved in 0.7 mL acetonitrile-d_3. The solution was degassed by applying a vacuum and flushing with argon three times. The reaction was indicated by a change of colour from bright red to dark blue only few minutes after starting the reaction. To reach equilibrium the reaction mixture was heated to 40 °C for 16 hours. Then, a ^{1}H-NMR spectrum was measured to evaluate the reaction progress without any work up. In order to isolate **BP-2**$(OTf)_6(BF_4)_4$ the reaction mixture was added drop wise to 25 mL of diethyl ether. The resulting suspension was stirred for 60 minutes at room temperature and then the blue precipitant was collected and washed with generous amounts of diethyl ether. Drying in a stream of air gave **BP-2**$(OTf)_6(BF_4)_4$ as a blue solid in 97% yield (11.13 mg, 2.64 µmol).

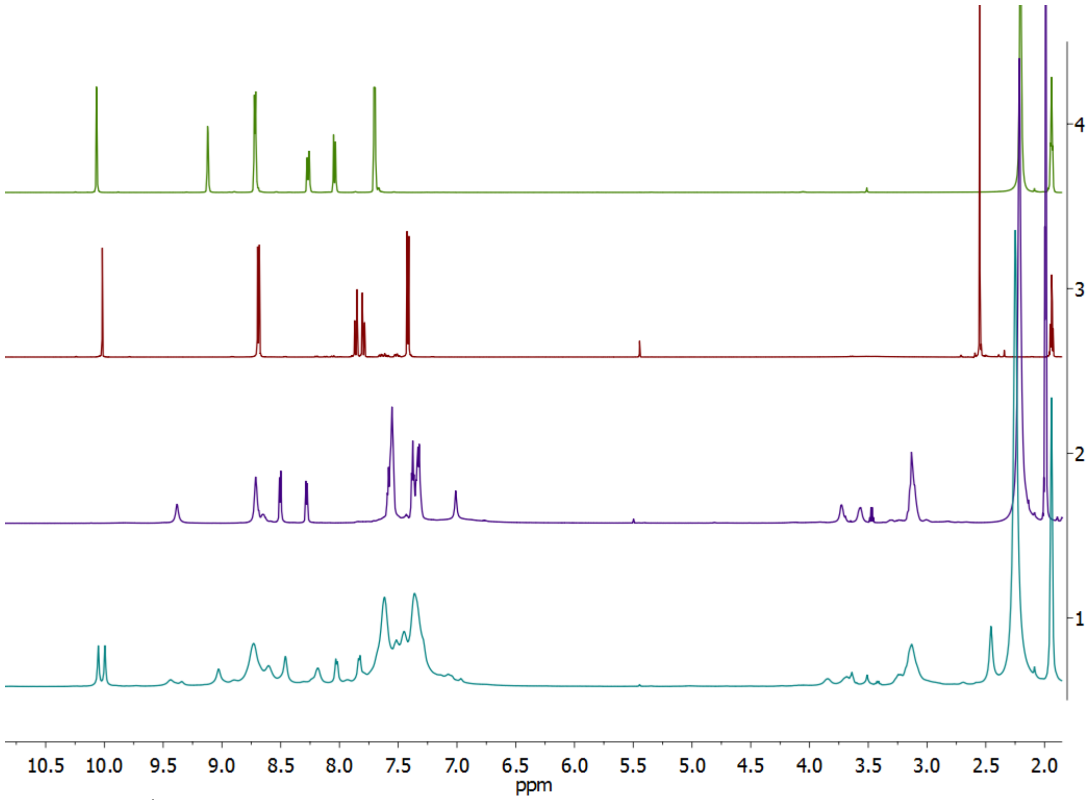

Figure S43. Stacked ^{1}H-NMR spectra of: [1] (400 MHz, acetonitrile-d_3, 298 K) the complex-to-complex transformation from **BP-1** to **BP-2**[6] (Scheme S4); [2] (700 MHz, acetonitrile-d_3, 298 K) **BP-2**$(OTf)_6(BF_4)_4$; [3] (500 MHz, acetonitrile-d_3, 298 K) **1**; [4] (400 MHz, acetonitrile-d_3, 298 K) **3**.

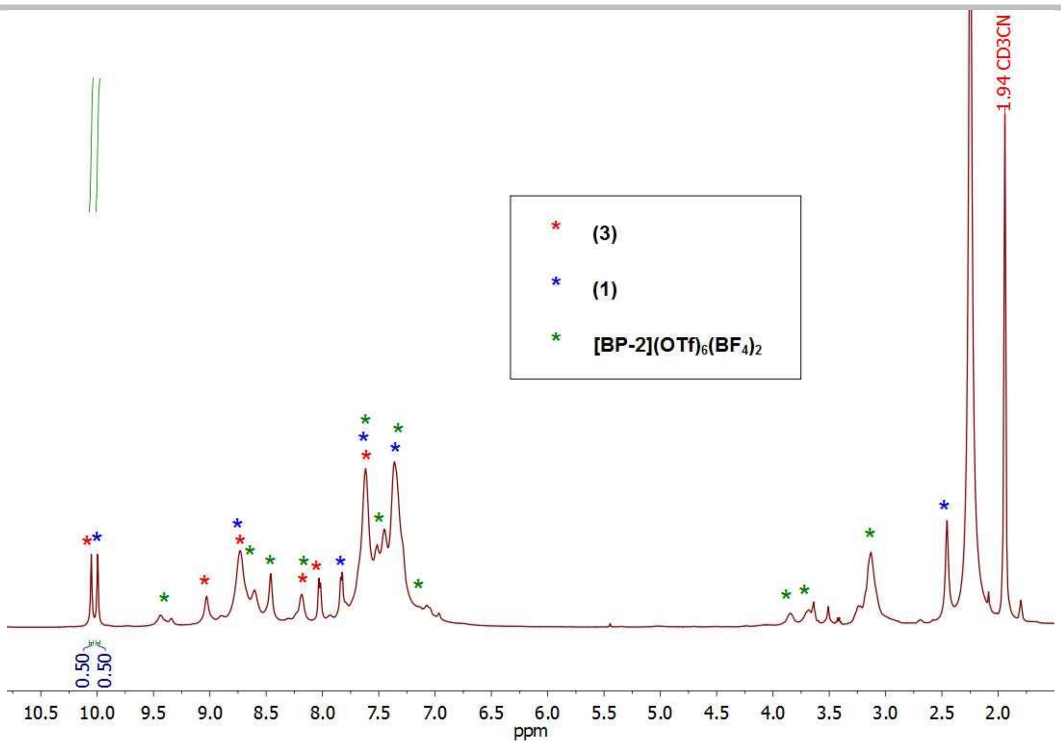

Figure S44. ^{1}H-NMR (400 MHz, acetonitrile-d_3, 298 K) of the complex-to-complex transformation from **BP-1** to **BP-2**[6] (Scheme S4). The corresponding aldehyde signals of **1** and **3** show an equilibrium ratio of 50:50.

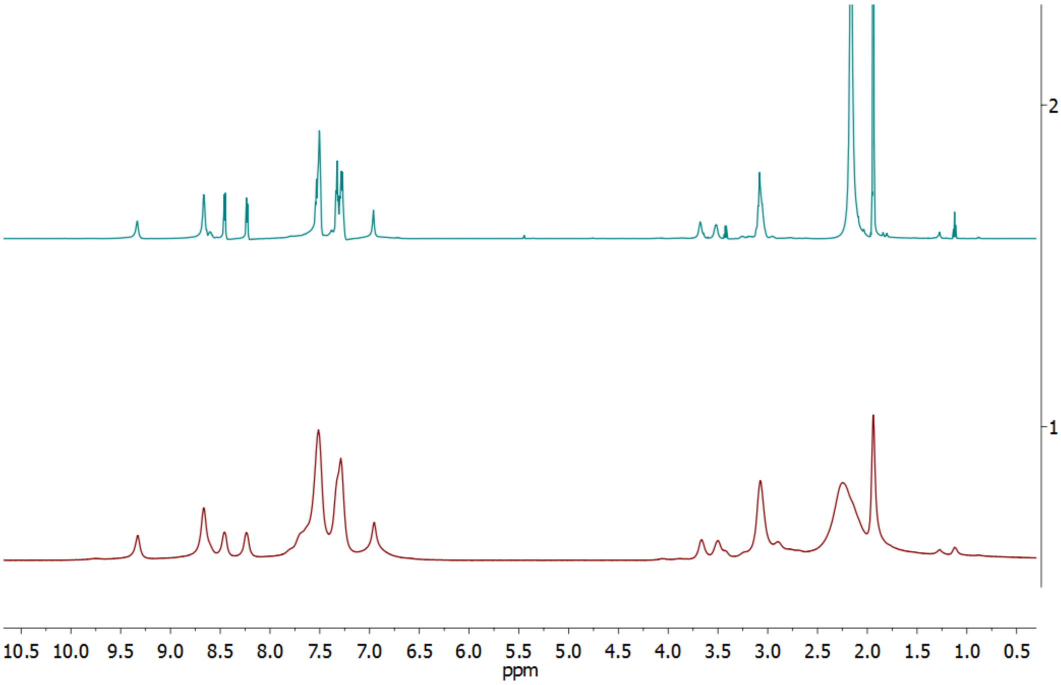

Figure S45. Stacked ^{1}H-NMR spectra of: top: **BP-2**$(OTf)_6(BF_4)_4$ (700 MHz, acetonitrile-d_3, 298 K); bottom: isolated **BP-2**$(OTf)_6(BF_4)_4$ (400 MHz, acetonitrile-d_3, 298 K) from the complex-to-complex transformation from **BP-1** to **BP-2** (Scheme S4).

Comparison of the ^{1}H-NMR spectra of **BP-2**$(OTf)_6(BF_4)_4$ and the spectrum received from the isolated complex of the complex-to-complex transformation according to scheme 4 indicates the successful formation of **BP-2**$(OTf)_6(BF_4)_4$ (Figure S45).
An Evans' experiment with the isolated complex also revealed, that **BP-2**$(OTf)_6(BF_4)_4$ from the complex-to-complex transformation shows no more magnetic moment, since the same signal for *t*-BuOH from the internal and external reference was found (Figure S46).

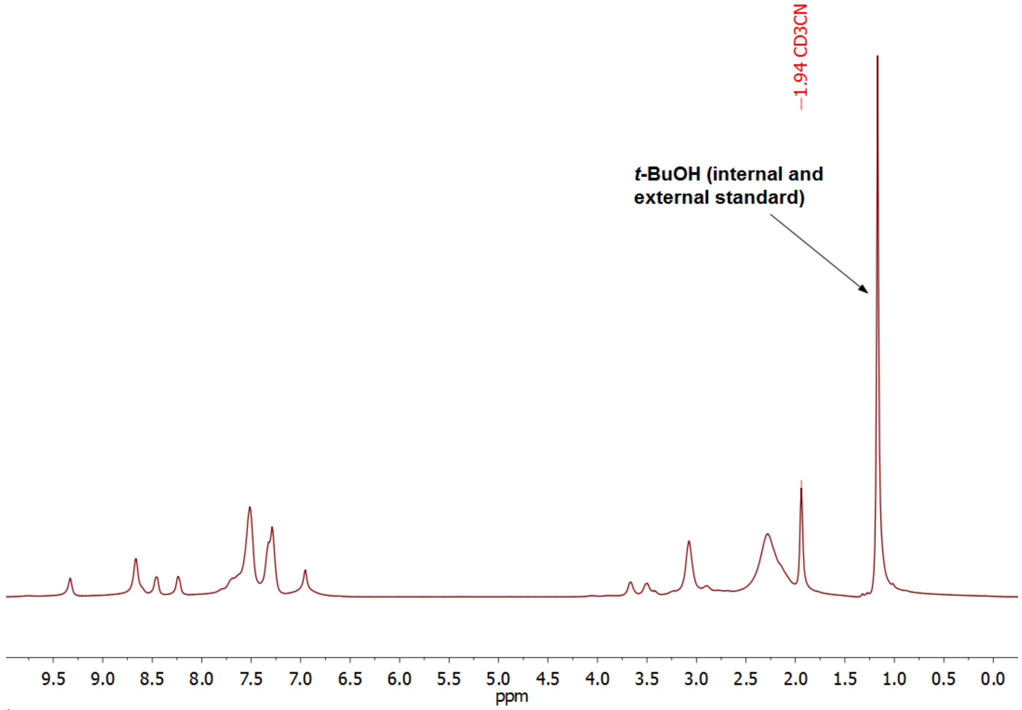

Figure S46. ^{1}H-NMR spectrum (400 MHz, acetonitrile-d_3, 298 K) of the Evans' experiment with isolated **BP-2**$(OTf)_6(BF_4)_4$ from the complex-to-complex transformation from **BP-1** to **BP-2** (Scheme S4).

This complex-to-complex transformation is indicated by a significant change in colour. Starting from the bright red paramagnetic complex solution, the transformation yields a dark blue solution of the analogues diamagnetic complex (Figure S47).

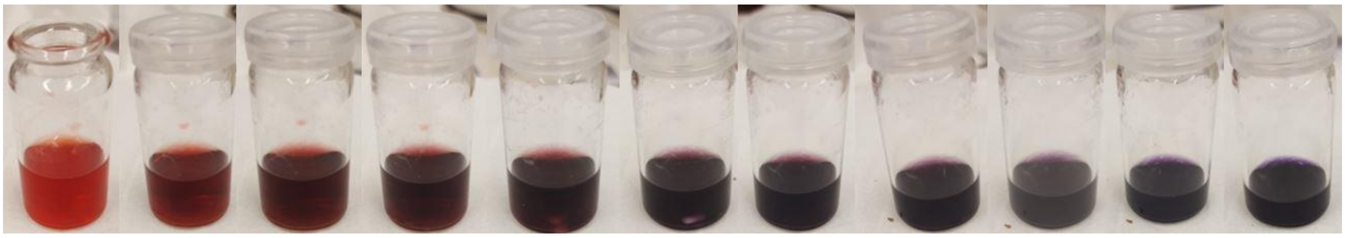

Figure S47. Colour change during the complex-to-complex transformation of **BP-1**$(OTf)_6(BF_4)_4$ to **BP-2**$(OTf)_6(BF_4)_4$. The reaction was carried out with 12 equivalents of aldehyde **3** at room temperature and the pictures show the reaction progress over 11.5 hours.

The reaction progress can be tracked by measuring a UV-vis spectrum of the reaction mixture after different reaction times (Figure S48). The increasing signal at 590 nm can be assigned to the $^{1}A_1 \rightarrow {}^{1}T_1$ transition of iron(II) cations of the diamagnetic bipyramid **BP-2**$(OTf)_6(BF_4)_4$ and therefore can be used to show the reaction progress.

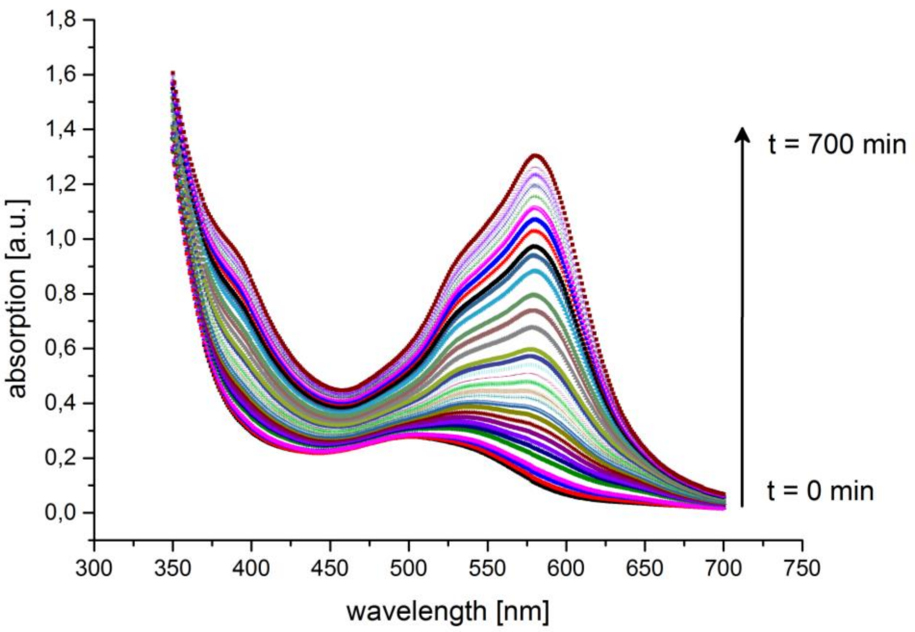

Figure S48. UV-vis spectra (acetonitrile, 1 mm cuvette, room temperature, c(**BP-2**(OTf)$_6$(BF$_4$)$_4$) = 0.9 mmol/L) of the complex-to-complex transformation of **BP-1**(OTf)$_6$(BF$_4$)$_4$ to **BP-2**(OTf)$_6$(BF$_4$)$_4$ taken every 30 minutes over a period of 700 minutes. The increasing signal located at 590 nm can be assigned to the $^1A_1 \rightarrow {}^1T_1$ transition of iron(II) cations of the diamagnetic bipyramid **BP-2**(OTf)$_6$(BF$_4$)$_4$.

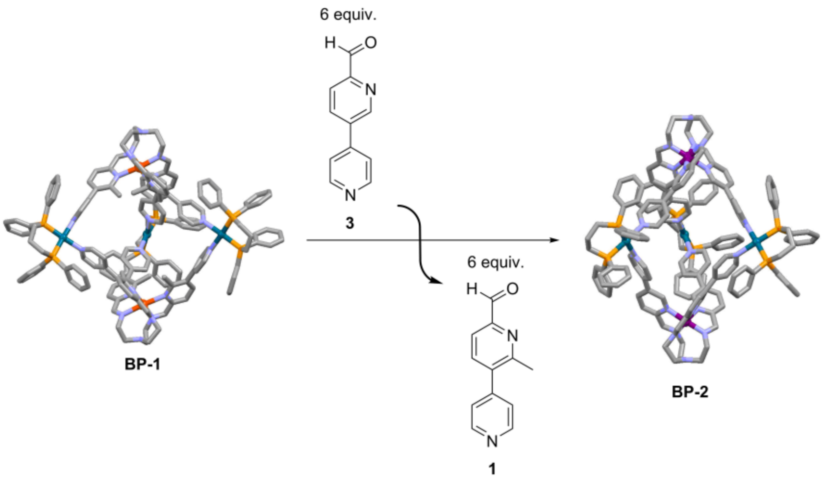

Scheme S5. Complex-to-complex transformation from **BP-1** to **BP-2** using 6 equiv of **3**.

BP-1(OTf)$_6$(BF$_4$)$_4$ (11.69 mg, 2.73 µmol, 1.00 equiv) together with 2-formyl-5-(4'-pyridyl) pyridine **3** (3.02 mg, 16.40 µmol, 6.00 equiv) was dissolved in 0.7 mL acetonitrile-d_3. The solution was degassed by applying a vacuum and flushing with argon three times. The reaction was indicated by a change of colour from bright red to dark blue only few minutes after starting the reaction. To reach equilibrium the reaction mixture was heated to 40 °C for 36 hours. Then, a ^{1}H-NMR spectrum was measured to evaluate the reaction progress without any work up. In order to isolate metal-organic compounds the reaction mixture was added drop wise to 25 mL of diethyl ether. The resulting suspension was stirred for 60 minutes at room temperature and then the bluish precipitant was collected and washed with generous amounts of diethyl ether. Drying in a stream of air gave 11.18 mg of a mixture of **BP-2**(OTf)$_6$(BF$_4$)$_4$ together with side products of unknown incorporated proportions of **1** and **3** as a blueish solid.

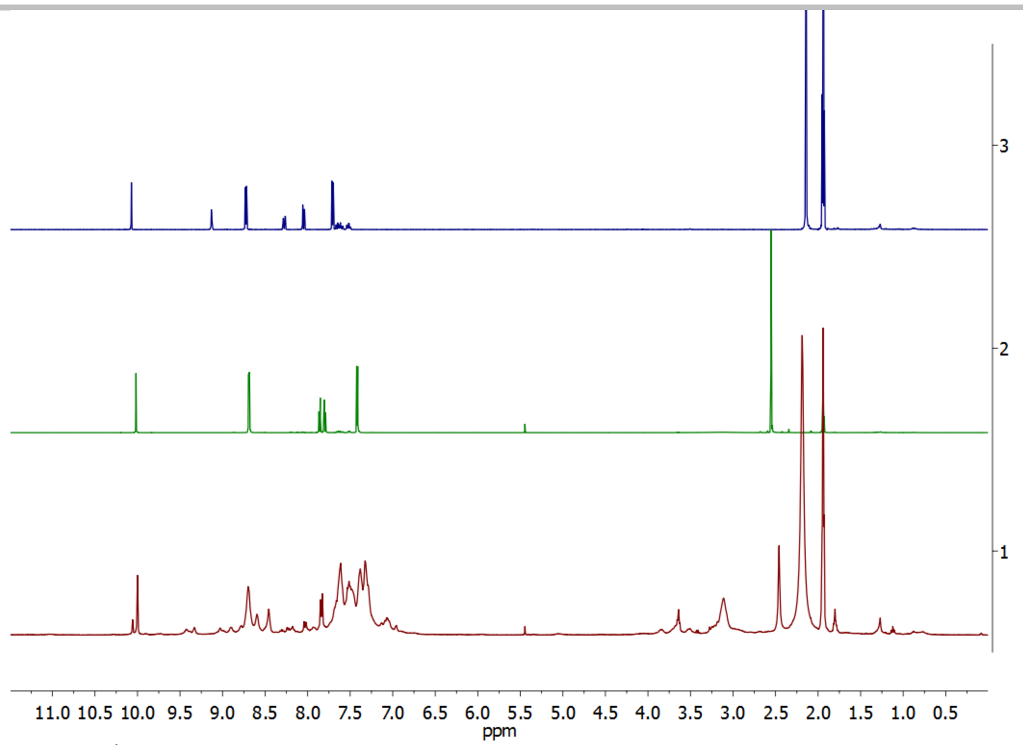

Figure S49. Stacked ^{1}H-NMR spectra of: [1] (400 MHz, acetonitrile-d_3, 298 K) the complex-to-complex transformation from **BP-1** to **BP-2**[6] (Scheme S5); [2] (500 MHz, acetonitrile-d_3, 298 K) **1**; [3] (400 MHz, acetonitrile-d_3, 298 K) **3**.

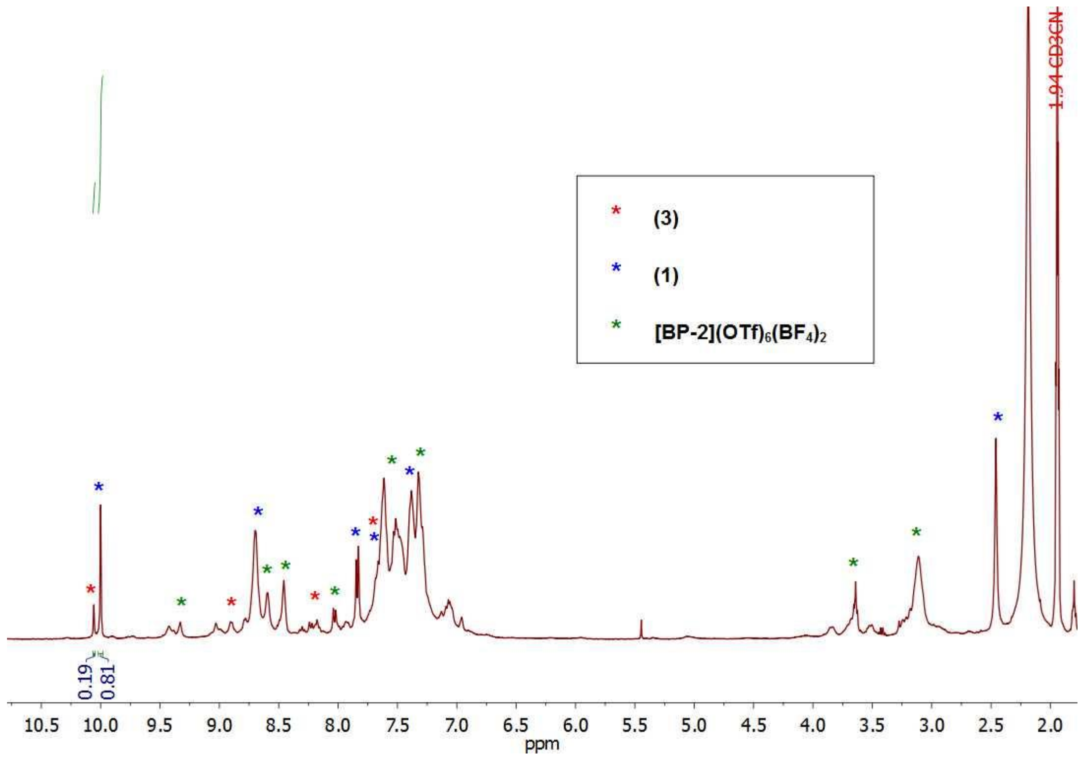

Figure S50. ^{1}H-NMR (400 MHz, acetonitrile-d_3, 298 K) of the complex-to-complex transformation from **BP-1** to **BP-2**[6] (Scheme S5). The corresponding aldehyde signals of **1** and **3** show an equilibrium ratio of 19:81.

Using only six equivalents of **3** to transform the paramagnetic bipyramid **BP-1** into the diamagnetic bipyramid **BP-2** does not lead to a quantitative reaction. About 20% of subcomponent **3** is still not incorporated into the aggregate after 36 hours, leading to some background signals of undefined structures. However, the equilibrium of the reaction is on the side of **BP-2**.

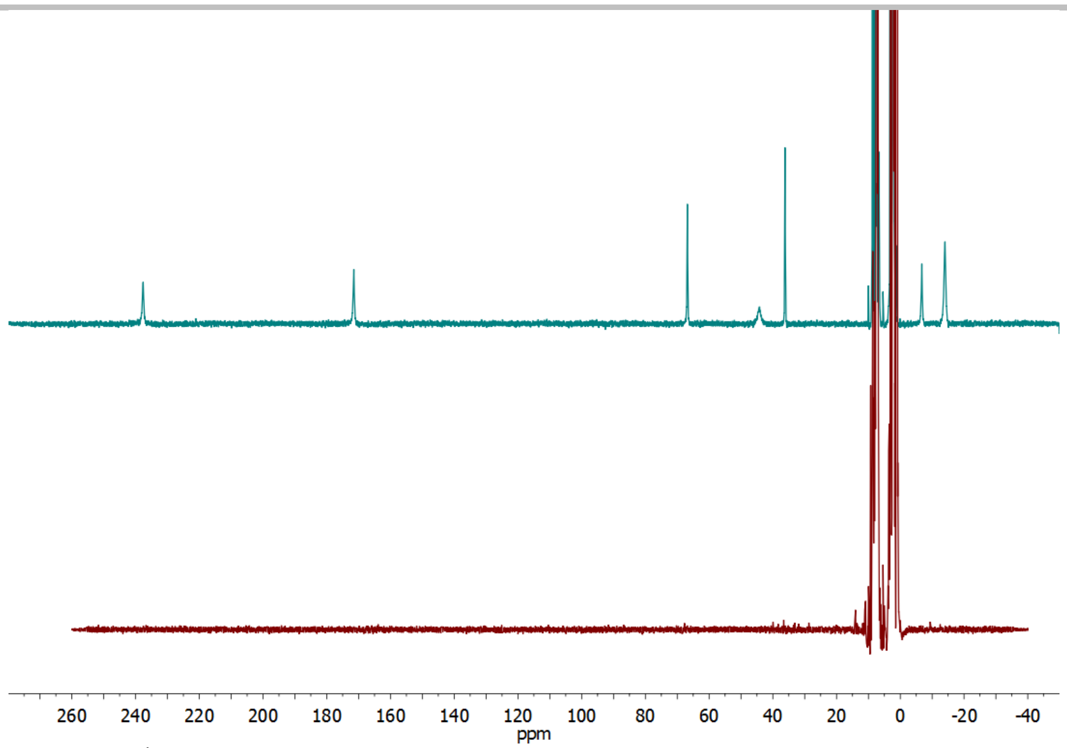

Figure S51. Stacked ^{1}H-NMR spectra of: top: (300 MHz, acetonitrile-d_3, 298 K) **BP-1**$(OTf)_6(BF_4)_4$; bottom: (300 MHz, acetonitrile-d_3, 298 K) isolated product mixture from the complex-to-complex transformation from **BP-1** to **BP-2** (Scheme S5).

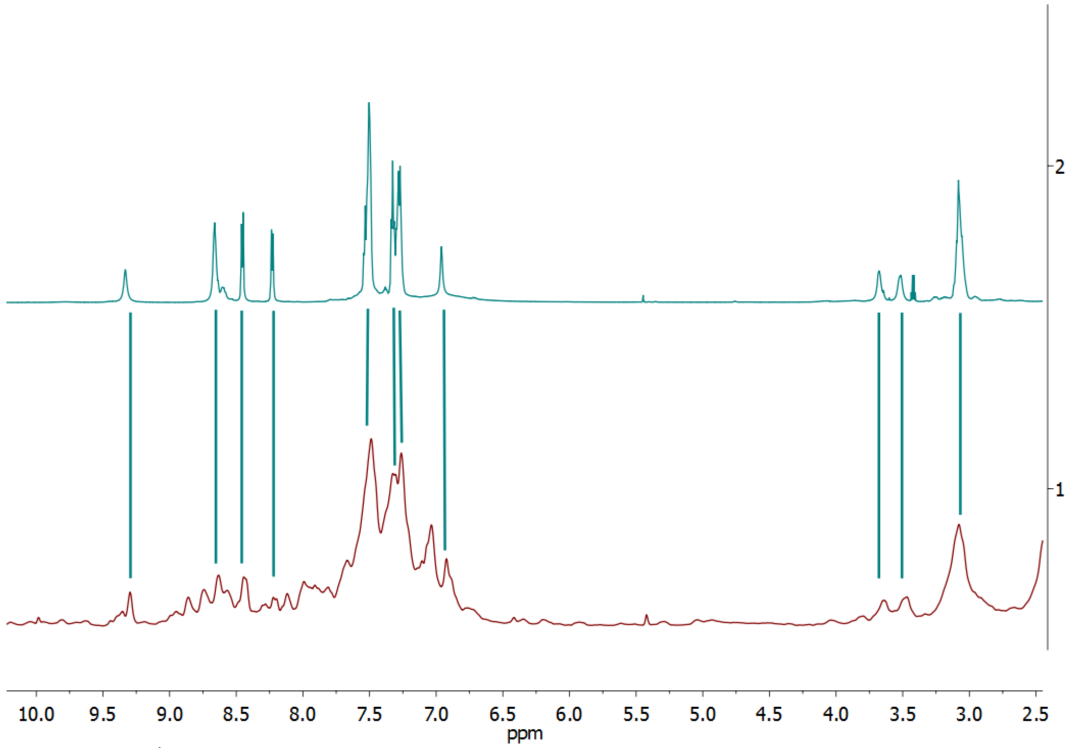

Figure S52. Stacked ^{1}H-NMR spectra of: top: (700 MHz, acetonitrile-d_3, 298 K) **BP-2**$(OTf)_6(BF_4)_4$; bottom: (300 MHz, acetonitrile-d_3, 298 K) isolated product mixture from the complex-to-complex transformation from **BP-1** to **BP-2** (Scheme S5). The blue-green lines are guides to the eye.

Comparison of the ^{1}H-NMR spectra of **BP-1**, **BP-2** and the product mixture received from the complex-to-complex transformation from **BP-1** to **BP-2** according to scheme S5 shows, that after the transformation no more signals of paramagnetic species were recorded (Figure S51) and **BP-2** was formed as a product in the reaction (Figure S52). However, the spectrum of the product mixture clearly shows that there is a significant amount of background signals, most likely

resulting from cages with unknown proportions of **1** and **3** incorporated. This is also corroborated by the finding that 20% of **3** was not incorporated into the cages after 36 hours (Figure S50).

The Evans' experiment with the isolated product mixture revealed a molar magnetic susceptibility multiplied with temperature of 1.38 cm^3 K mol^{-1} (Table S7, Figure S53) after the transformation, proving the existence of a small amount of paramagnetic species. Please note that for this calculation the molar mass of **BP-2**$(OTf)_6(BF_4)_4$ was used, since the composition of the side products in unknown. Therefore the calculated value for X_mT is not an exact value for the magnetic susceptibility.

Table S7: Magnetic susceptibility of the isolated product mixture from the transformation from **BP-1** to **BP-2** according to Scheme S5 in acetonitrile-d_3 as determined by the Evans' method. *The concentration was calculated using the molar mass of **BP-2**$(OTf)_6(BF_4)_4$.

concentration [mM]*	temperature [K]	Δδ (*t*-BuOH) [Hz]	X_mT [cm^3 K mol^{-1}]
6.69	298	39.02	1.38

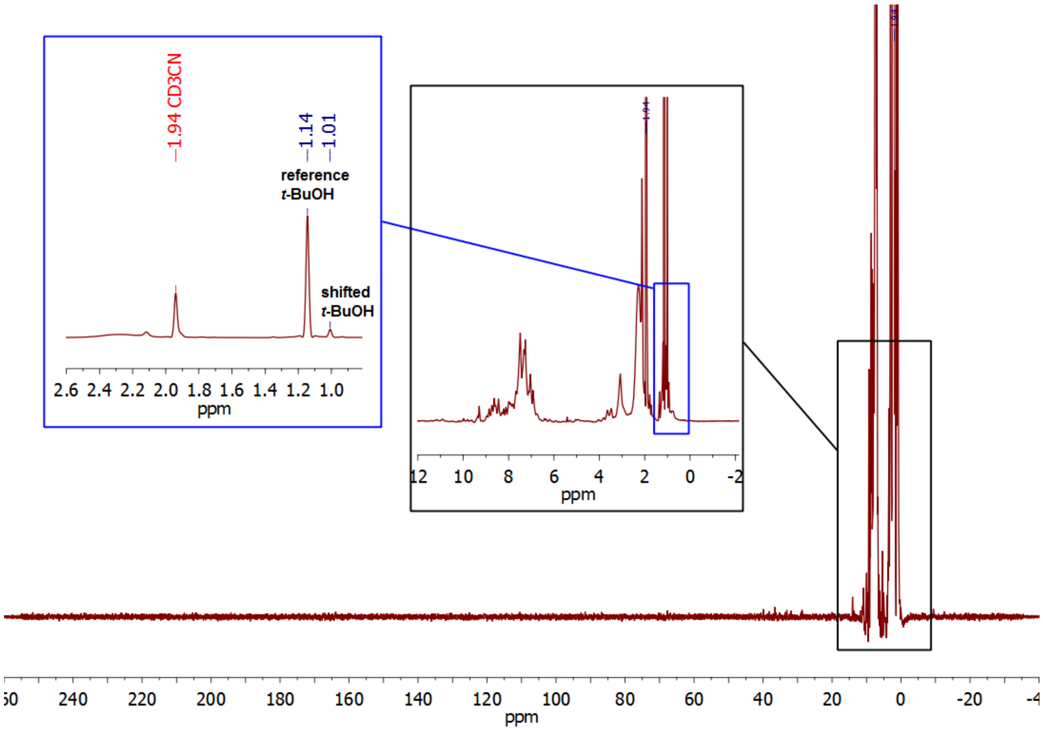

Figure S53. Evans' experiment (300 MHz, acetonitrile-d_3, 298 K) of the isolated product mixture from the complex-to-complex transformation according to Scheme 5.

CU-1$(BF_4)_{28}$ to **CU-2**$(BF_4)_{28}$

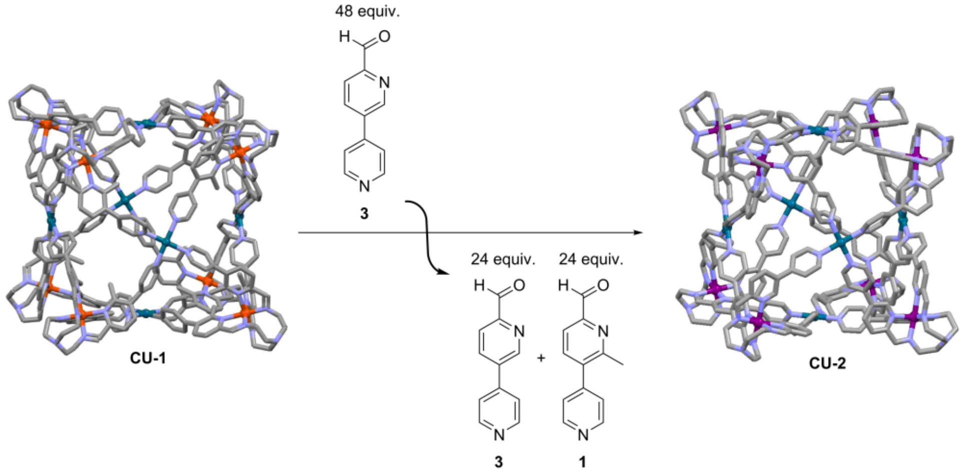

Scheme S6. Complex-to-complex transformation from **CU-1** to **CU-2** using 48 equiv of **3**.

CU-1$(BF_4)_{28}$ (6.82 mg, 0.68 µmol, 1.00 equiv) together with 2-formyl-5-(4'-pyridyl) pyridine **3** (6.03 mg, 32.70 µmol, 48.00 equiv) was dissolved in 0.7 mL acetonitrile-d_3. The solution was degassed by applying a vacuum and flushing with argon three times. The reaction was indicated by a change of colour from bright red to dark blue only few minutes after starting the reaction. To reach equilibrium the reaction mixture was heated to 50 °C for 3 days. Then, a ^{1}H-NMR spectrum was measured to evaluate the reaction progress without any work up. In order to isolate metal-organic compounds the reaction mixture was added drop wise to 25 mL of diethyl ether. The resulting suspension was stirred for 30 minutes at room temperature and then the bluish precipitant was collected and washed with generous amounts of diethyl ether. Drying in a stream of air gave 6.18 mg of a mixture of **CU-2**$(BF_4)_{28}$ together with side products of unknown incorporated proportions of **1** and **3** as a bluish solid. The product was hardly soluble in acetonitrile.

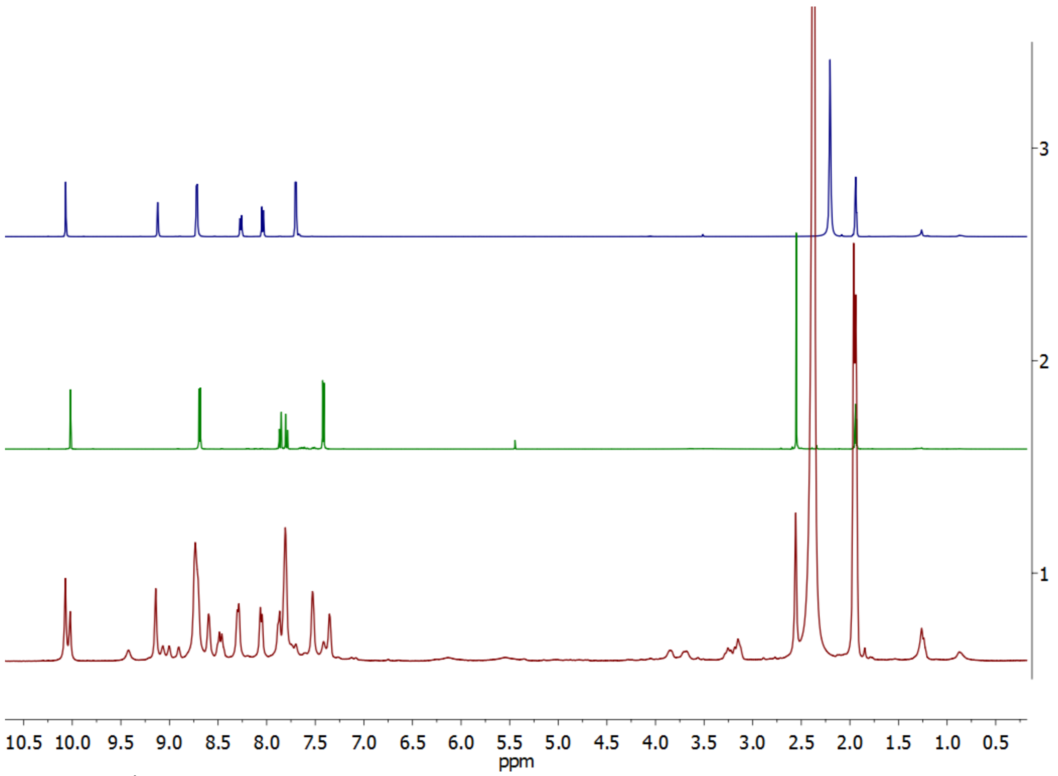

Figure S54. Stacked ^{1}H-NMR spectra of: [1] (400 MHz, acetonitrile-d_3, 298 K) the complex-to-complex transformation from **CU-1** to **CU-2**[6] (Scheme S6); [2] (500 MHz, acetonitrile-d_3, 298 K) **1**; [3] (400 MHz, acetonitrile-d_3, 298 K) **3**.

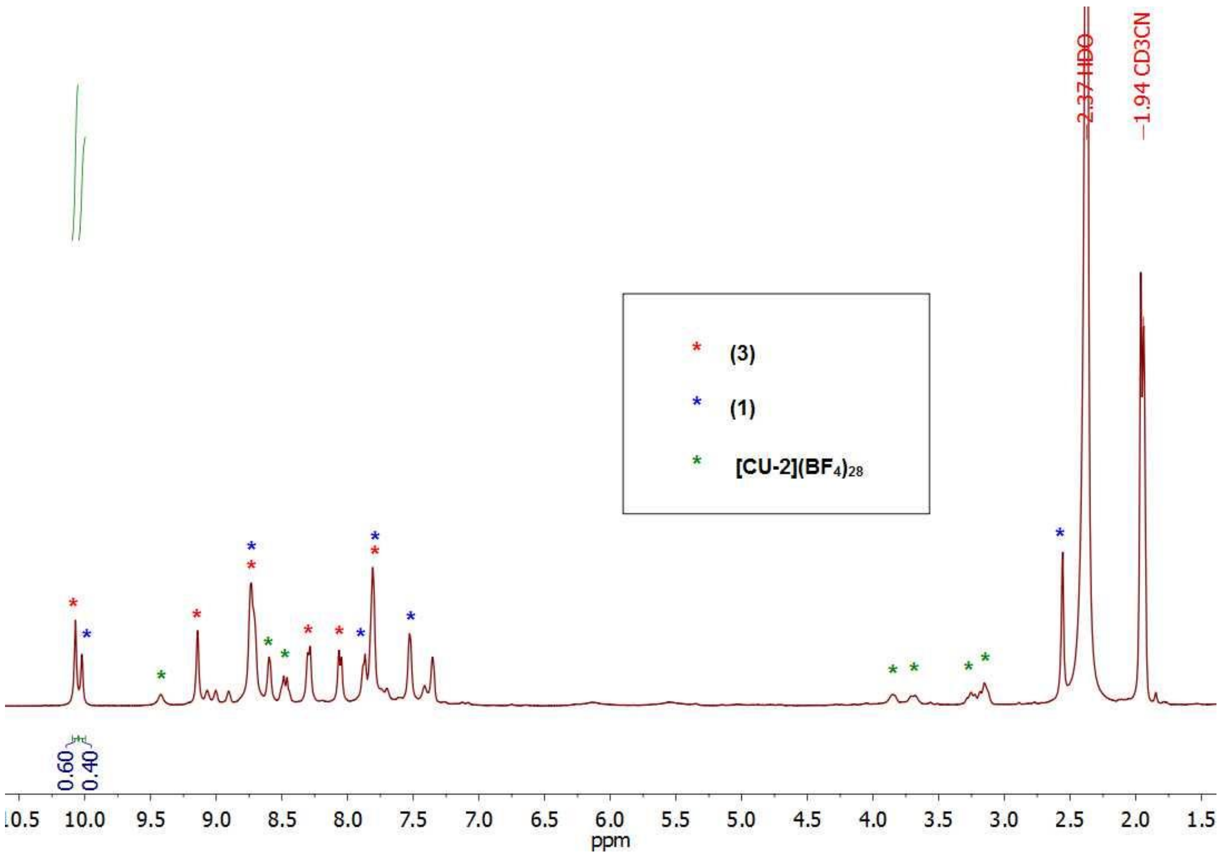

Figure S55. ^{1}H-NMR (400 MHz, acetonitrile-d_3, 298 K) of the complex-to-complex transformation from **CU-1** to **CU-2**[6] (Scheme S6). The corresponding aldehyde signals of **1** and **3** show an equilibrium ratio of 40:60.

The spectrum in Figure S55 shows that the complex-to-complex transformation from **CU-1** to **CU-2** did not lead to a quantitative subcomponent exchange, although 48 equivalents of aldehyde **3** were used. The equilibrium ratio between **1** and **3** was determined to be 40:60. As consequents some background signals that do not refer to **1**, **3** nor **CU-2** are observed and probably belong to heterometallic species with unknown proportions of **1** and **3**. Please note, that one would expect a 2:1 ratio of **3**:**1** if the exchange would happen in a statistical manner without any driving force. Therefore, this experiment shows that incorporation of **3** into the cubic assembly is slightly more favoured than incorporation of **1**, but less pronounced than in the case of the bipyramidal assemblies.

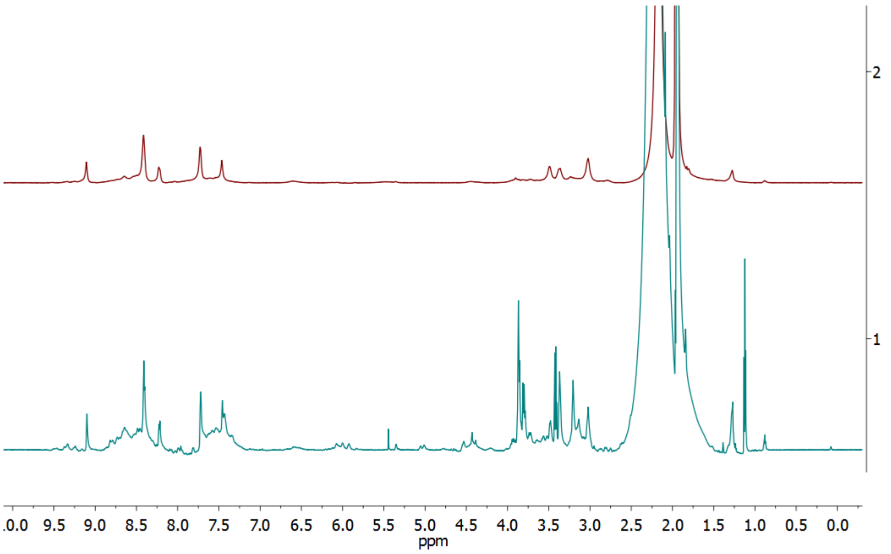

Figure S56. Stacked ^{1}H-NMR spectra of: top: (700 MHz, acetonitrile-d_3, 298 K) **CU-2**(BF_4)$_{28}$; bottom: (400 MHz, acetonitrile-d_3, 298 K) isolated product mixture from the complex-to-complex transformation from **CU-1** to **CU-2** (Scheme S6).

Comparison of the ^{1}H-NMR spectrum from the isolated product (Scheme S6) with the spectrum from **CU-2** (Figure S56) shows that **CU-2** was formed as the main product in this transformation. However, there is a significant amount of background signals, most probably from species with unknown proportions of **1** and **3** incorporated.

Unfortunately, we could not perform an Evans' experiment using the isolated complex mixture, due to the very low solubility of the complex in acetonitrile. Figure S57 shows that the complex concentration is not sufficient to observe shifted *t*-BuOH signals.

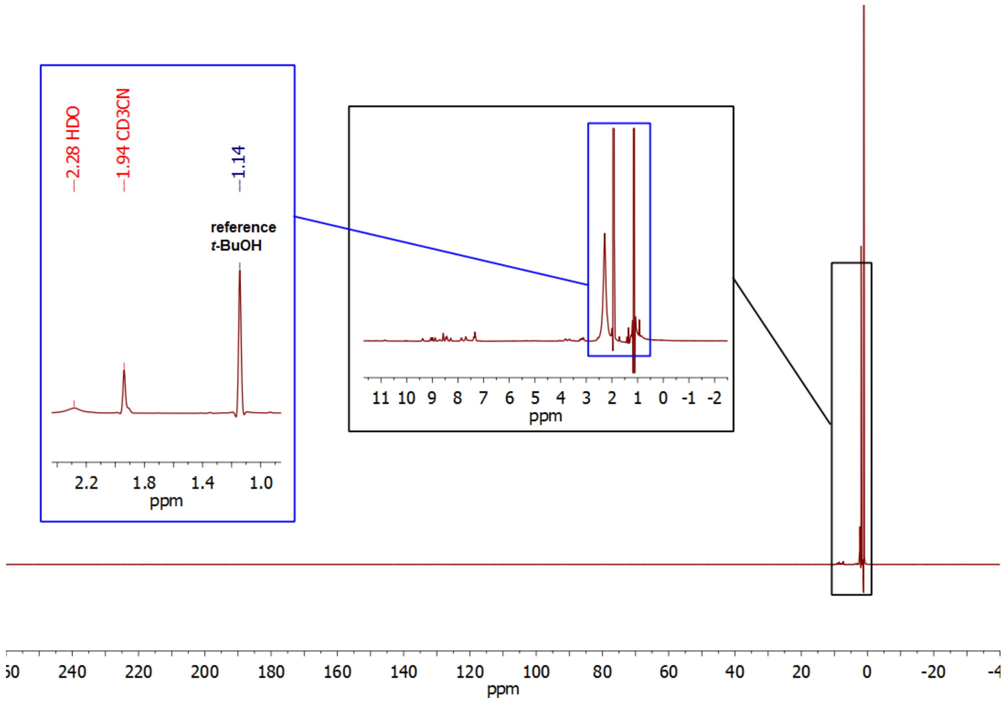

Figure S57. Evans' experiment (300 MHz, acetonitrile-d_3, 298 K) of the isolated product mixture from the complex-to-complex transformation according to Scheme 6.

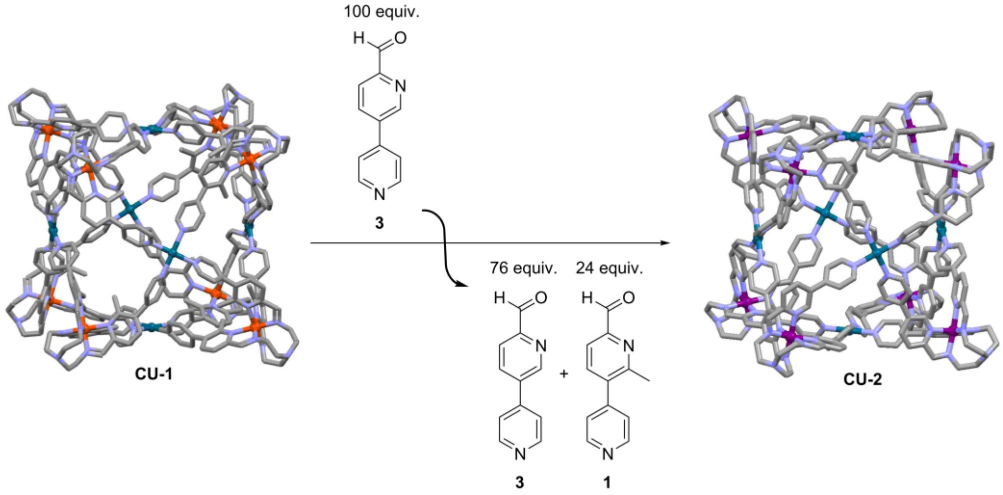

Scheme S7. Complex-to-complex transformation from **CU-1** to **CU-2** using 100 equiv of **3**.

ML-1(BF_4)$_2$ (5.00 mg, 5.46 µmol, 8.00 equiv) and *tetrakis*(acetonitrile)palladium(II) tetrafluoroborate ([Pd(CH_3CN)$_4$](BF_4)$_2$; 1.82 mg, 4.09 µmol, 6.00 equiv) in 0.7 mL of acetonitrile-d_3 was degassed by applying a vacuum and flushing with argon three times and heated under an argon atmosphere at 50 °C for 16 hours. Then, the reaction mixture was cooled down to room temperature and 2-formyl-5-(4'-pyridyl) pyridine **3** (12.66 mg, 68.20 µmol, 100.00 equiv) was added. The solution was degassed again. The reaction was indicated by a change of colour from bright red to dark blue only few minutes after starting the reaction. To reach equilibrium the reaction mixture was heated to 65 °C for 3 days. Then, a ^{1}H-NMR spectrum was measured to evaluate the reaction progress without any work up. In order to isolate metal-organic compounds the reaction mixture was added drop wise to 25 mL of diethyl ether. The resulting suspension was stirred for 30 minutes at room temperature and then the bluish precipitant was collected and washed with generous amounts of diethyl ether. Drying in a stream of air gave 6.03 mg of a mixture of **CU-2**(BF_4)$_{28}$ as main product together with side products of unknown incorporated proportions of **1** and **3** as a bluish solid. The product was hardly soluble in acetonitrile.

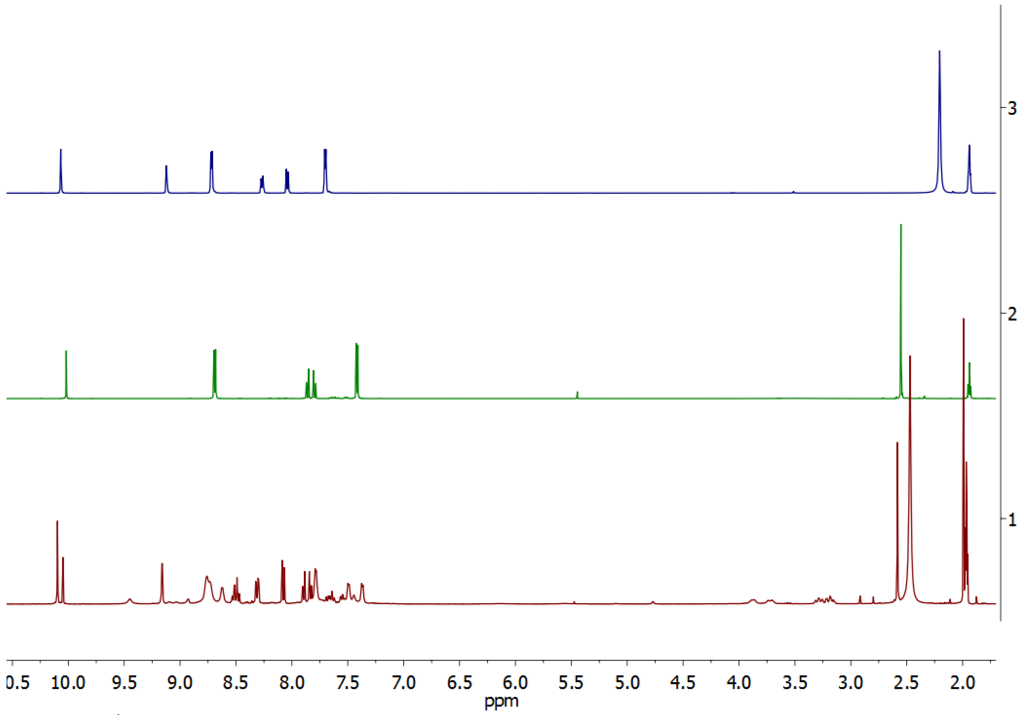

Figure S58. Stacked ^{1}H-NMR spectra of: [1] (400 MHz, acetonitrile-d_3, 298 K) the complex-to-complex transformation from **CU-1** to **CU-2**[6] (Scheme S7); [2] (500 MHz, acetonitrile-d_3, 298 K) **1**; [3] (400 MHz, acetonitrile-d_3, 298 K) **3**.

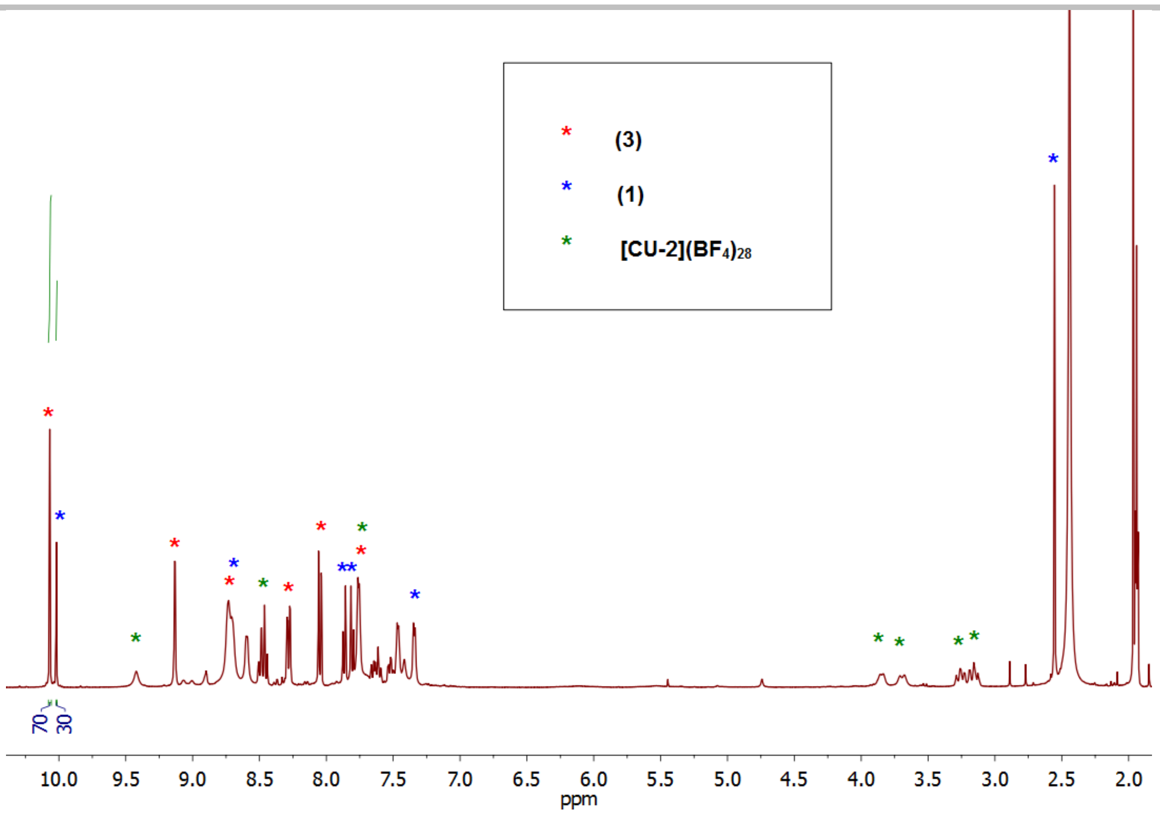

Figure S59. ^{1}H-NMR (400 MHz, acetonitrile-d_3, 298 K) of the complex-to-complex transformation from **CU-1** to **CU-2**[6] (Scheme S7). The corresponding aldehyde signals of **1** and **3** show an equilibrium ratio of 30:70.

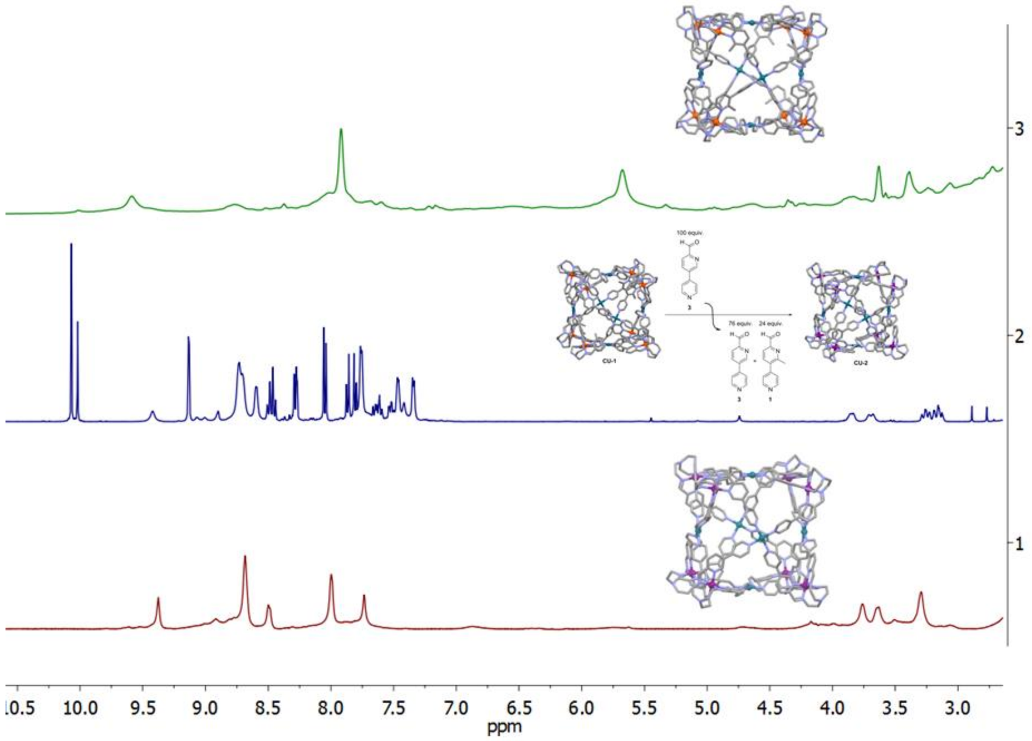

Figure S60. Excerpts of ^{1}H-NMR spectra of: Top (400 MHz, acetonitrile-d_3, 298 K) **CU-1**; Middle (400 MHz, acetonitrile-d_3, 298 K) complex-to-complex transformation from **CU-1** to **CU-2** using 100 equiv of **3**; Bottom (500 MHz, acetonitrile-d_3, 298 K) **CU-2**.

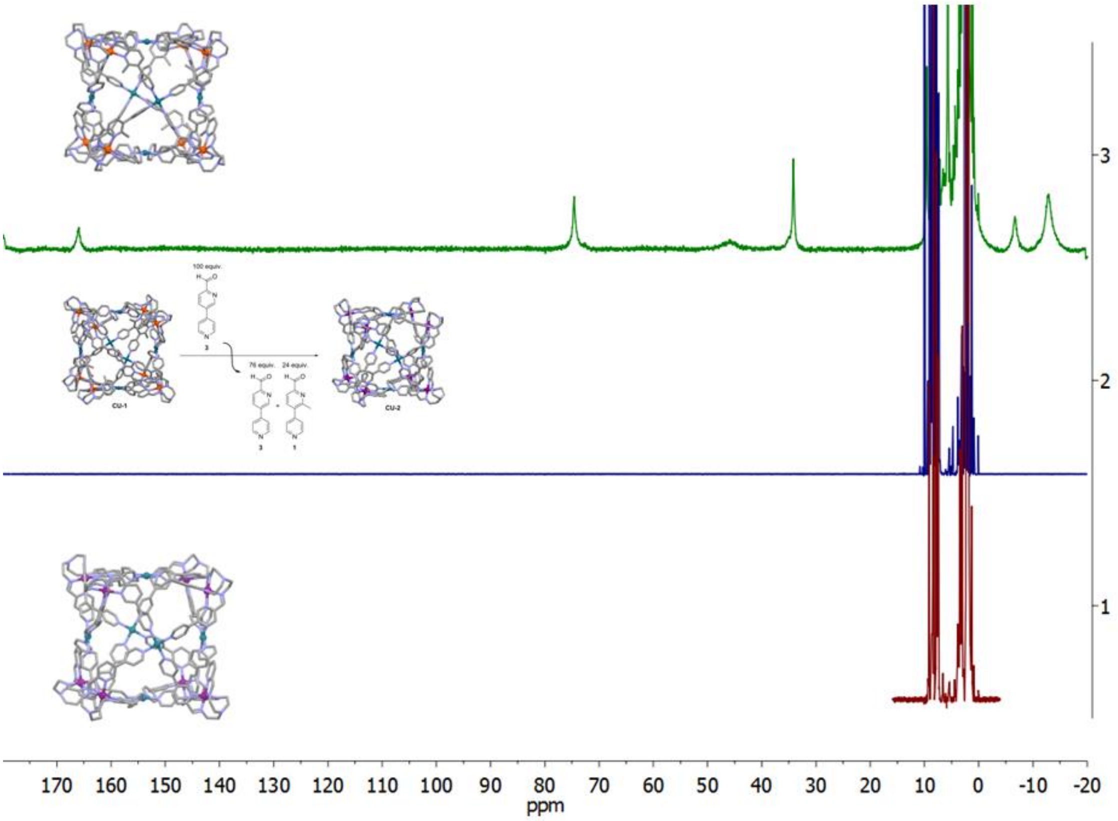

Figure S61. Overall ^{1}H-NMR spectra of: Top (400 MHz, acetonitrile-d_3, 298 K) **CU-1**; Middle (400 MHz, acetonitrile-d_3, 298 K) complex-to-complex transformation from **CU-1** to **CU-2** using 100 equiv of **3**; Bottom (500 MHz, acetonitrile-d_3, 298 K) **CU-2** not measured as a wide sweep spectrum.

The complex-to-complex transformation of **CU-1** to **CU-2** using 100 equiv of aldehyde **3** yielded a conservatively estimated ratio of **1** to **3** of 30:70 (Figure S59), corresponding to a nearly quantitative transformation with an expected ratio of 24:76. We note that we observed precipitation of a dark blue compound during the transformation experiment - this is most likely the diamagnetic **CU-2**. However, the precipitate might also contain small amounts of the less soluble aldehyde **3** which might lead to an overestimation of the relative amount of the better soluble aldehyde **1**. Please note that we could not perform experiments with higher amounts of **3** due to its limited solubility. We do not observe any signals referring to **CU-1** after the reaction (Figures S60 and S61).

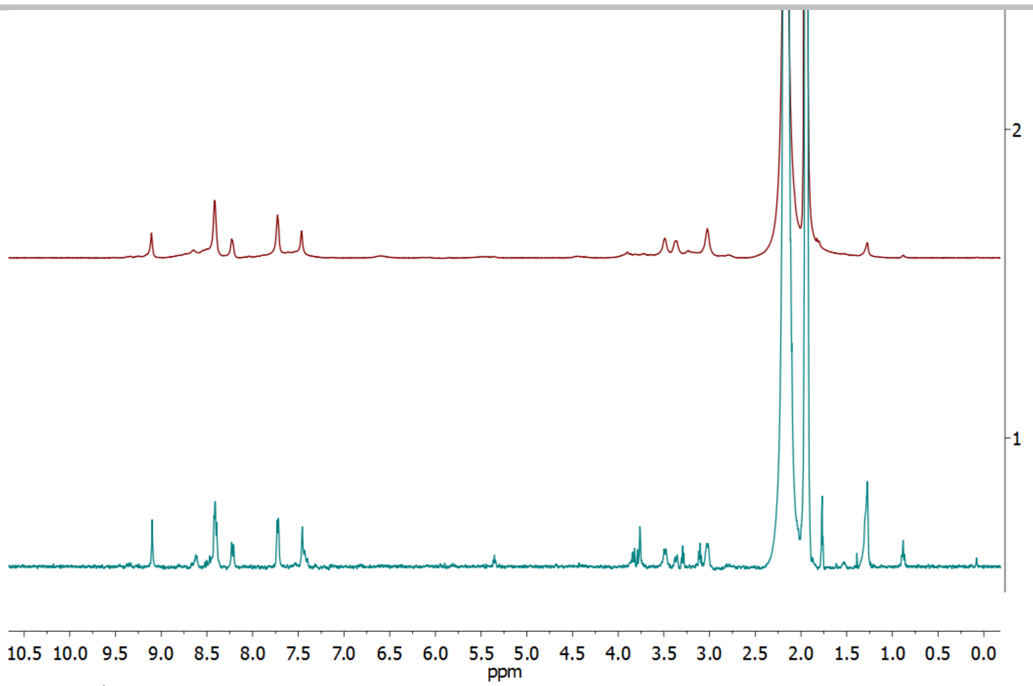

Figure S62. Stacked ^{1}H-NMR spectra of: top: (700 MHz, acetonitrile-d_3, 298 K) **CU-2**(BF_4)$_{28}$; bottom: (400 MHz, acetonitrile-d_3, 298 K) isolated product mixture from the complex-to-complex transformation from **CU-1** to **CU-2** (Scheme S7).

Comparison of the ^{1}H-NMR spectrum from the isolated product (Scheme S7) with the spectrum from **CU-2** (Figure S62) shows that **CU-2** was formed as the main product in this transformation. However, there is a minor amount of background signals that could be the result of the low solubility of **CU-2** in acetonitrile and an overestimation of impurities.

Unfortunately, we could not perform an Evans' experiment using the isolated complex mixture, due to the very low solubility of the complex in acetonitrile. Figure S63 shows that the complex concentration is hardly sufficient to observe shifted *t*-BuOH signals. However, from the aldehyde ratio observed in the previous ^{1}H-NMR spectrum (Figure S59) we would not expect to observe a splitted *t*-BuOH signal, due to the nearly quantitative consumption of aldehyde **3**.

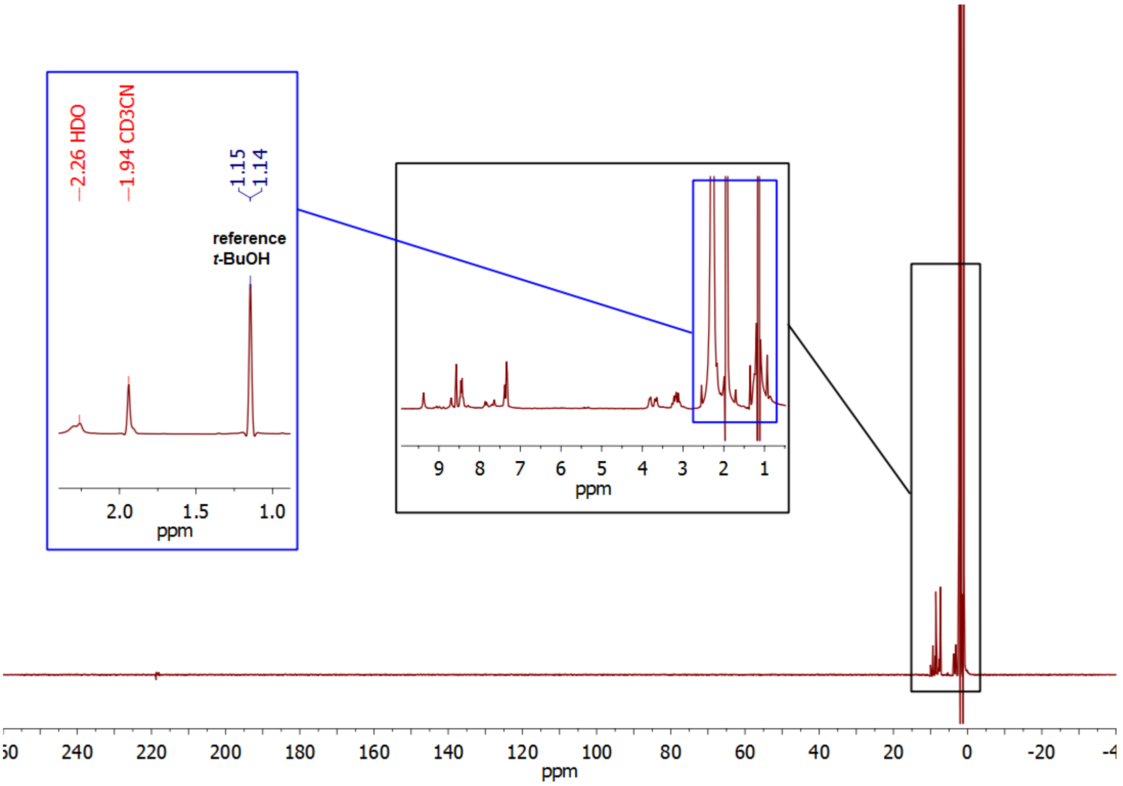

Figure S63. Evans' experiment (300 MHz, acetonitrile-d_3, 298 K) of the isolated product mixture from the complex-to-complex transformation according to Scheme 7.

CU-1(BF_4)$_{28}$ to **BP-1**(BF_4)$_{10}$ and **ML-1** (BF_4)$_2$

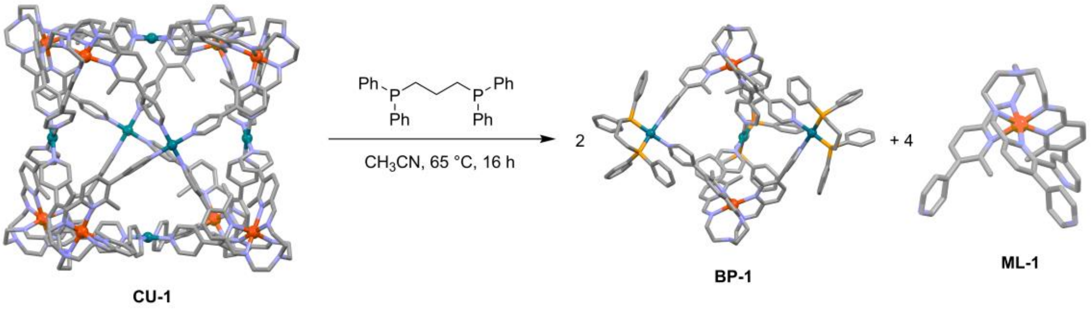

Scheme S8. Complex-to-complex transformation from **CU-1** to **BP-1** and **ML-1**.

ML-1(BF_4)$_2$ (8.00 mg, 8.73 µmol, 8.00 equiv.) together with *tetrakis*(acetonitrile)palladium(II) tetrafluoroborate ([Pd(CH_3CN)$_4$](BF_4)$_2$; 2.91 mg, 6.55 µmol, 6.00 equiv) in 0.7 mL of acetonitrile-*d₃* was degassed by applying a vacuum and flushing with argon three times and heated under an argon atmosphere at 50 °C for 16 hours to assemble **CU-1**. After cooling to room temperature 1,3-*bis*(diphenylphosphino)propane (dppp; 2.70 mg, 6.55 µmol, 6.00 equiv) was added. The resulting solution was degassed again three times and heated under an argon atmosphere at 65 °C for 16 hours. Then, a ^{1}H-NMR spectrum was measured to evaluate the reaction progress without any work up. In order to isolate the product mixture, the solution was added to 25 mL of diethyl ether drop wise and was stirred for 30 minutes. Then, the red precipitant was collected, washed with generous amounts of diethyl ether and dried in air. The product mixture was obtained as a red solid (11.6 mg).
We observed the formation of a considerable amount of insoluble red precipitate, when we heated the solution to 50 °C after addition of dppp, probably due to the formation of non-discrete oligomers. Subsequent heating to 65 °C led to even more precipitation and non-defined signals in the ^{1}H-NMR spectrum. Immediate heating of the solution to 65 °C instead, yielded a clear red solution (Figure S64).

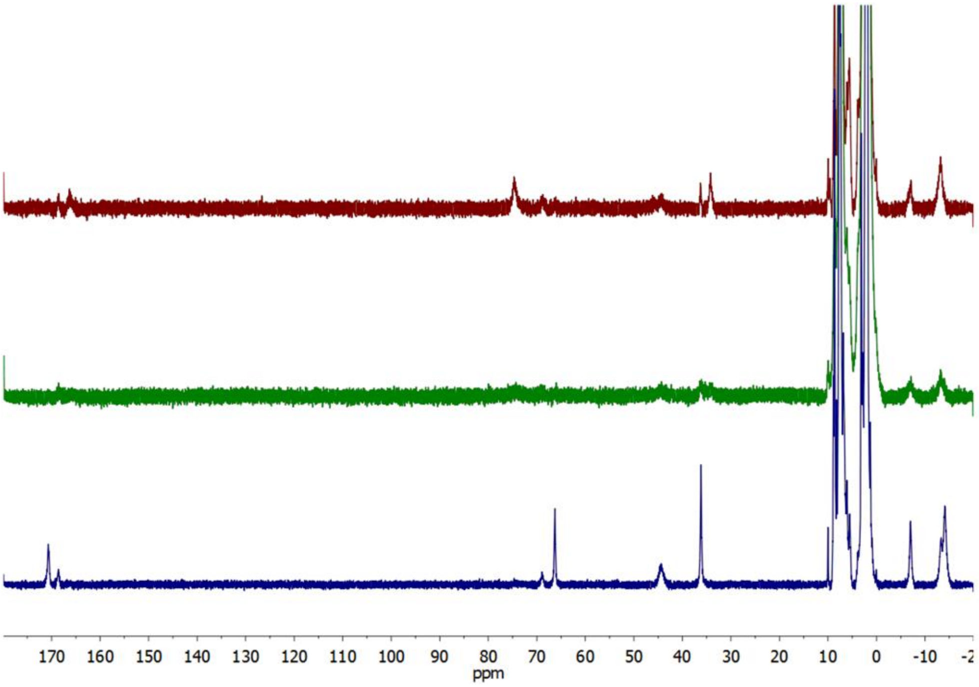

Figure S64. Wide sweep ^{1}H-NMR spectra of: Top (400 MHz, acetonitrile-d_3, 298 K) **CU-1** + 6 equiv. dppp after 16 hours at 50 °C; Middle (400 MHz, acetonitrile-d_3, 298 K) **CU-1** + 6 equiv. dppp after 16 hours at 50 °C followed by 20 hours at 65 °C; Bottom (400 MHz, acetonitrile-d_3, 298 K) **CU-1** + 6 equiv. dppp after 16 hours at 65 °C.

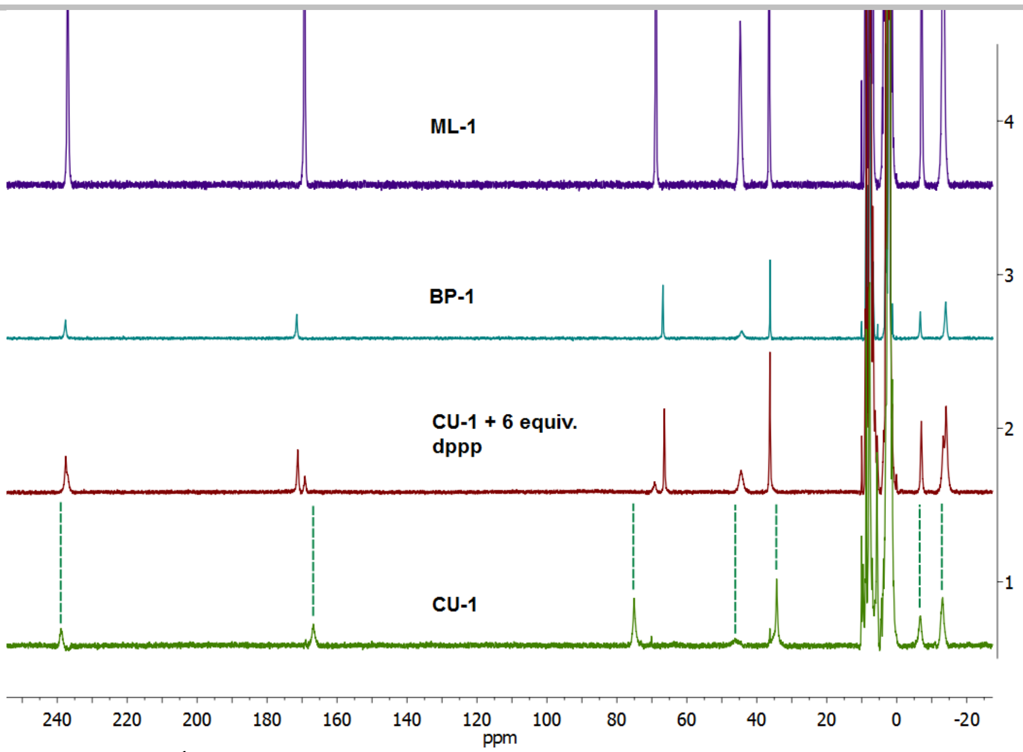

Figure S65. Wide sweep ^{1}H-NMR spectra of: Top (300 MHz, acetonitrile-d_3, 298 K) **ML-1**; Second from top (300 MHz, acetonitrile-d_3, 298 K) **BP-1**; Second from bottom (300 MHz, acetonitrile-d_3, 298 K) **CU-1** + 6 equiv. dppp after 16 hours at 65 °C; Bottom (300 MHz, acetonitrile-d_3, 298 K) **CU-1**. The green dotted lines are guides to the eye in order to show the absence of signals referring to **CU-1** after the complex-to-complex transformation.

Figure S65 shows that the wide sweep ^{1}H-NMR spectrum of the reaction solution 16 hours after addition of 6 equivalents of dppp and after heating to 65 °C does not show any signals referring to the initial cage **CU-1** anymore. Instead, the observed spectrum is an overlay of the ^{1}H-NMR spectra of the bipyramidal cage **BP-1** and metalloligand **ML-1**. Please note that we did not observe any influences of the counter anions on the ^{1}H-NMR spectrum of **BP-1**. Therefore we used the ^{1}H-NMR spectrum of **BP-1**$(OTf)_6(BF_4)_4$ for this stack.
Since the spectrum we received from this transformation is a fitting overlay of the ^{1}H-NMR spectra of **ML-1** and **BP-1**, the magnetic susceptibility should be the same as found with the pure complexes. To further prove that we performed an Evans' experiment with the 1:2 mixture of **BP-1** and **ML-1** from the complex-to-complex transformation. The calculated value for X_mT (per iron(II) cation) is 3.06 cm^{3} K mol^{-1}, which fits the theoretical value of 3.001 cm^{3} K mol^{-1} per uncoupled iron(II) cation[5] in the high-spin state, showing that the magnetic properties are maintained in this reaction (Table S8, Figure S66).

Table S8: Magnetic susceptibility of the isolated product mixture from the transformation from **CU-1** to **BP-1** and **ML-1** (in a 1:2 ratio) according to Scheme S8 in acetonitrile-d_3 as determined by the Evans' method. *The concentration is given as the concentration of iron(II) cations.

concentration [mM]*	temperature [K]	Δδ (*t*-BuOH) [Hz]	X_mT [cm^{3} K mol^{-1}]
17.42	298	225.12	3.06

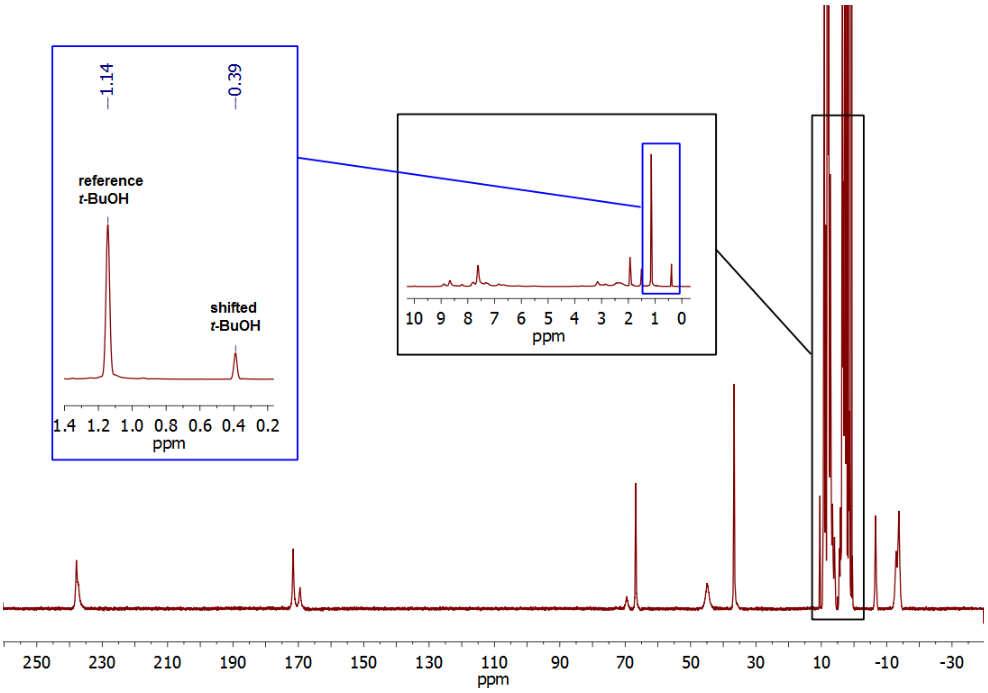

Figure S66. Evans' experiment (300 MHz, acetonitrile-d_3, 298 K) of the isolated product mixture from the complex-to-complex transformation from **CU-1** to **BP-1** and **ML-1** according to Scheme S8.

CU-2$(BF_4)_{28}$ to **BP-2**$(BF_4)_{10}$ and **ML-2**$(BF_4)_2$

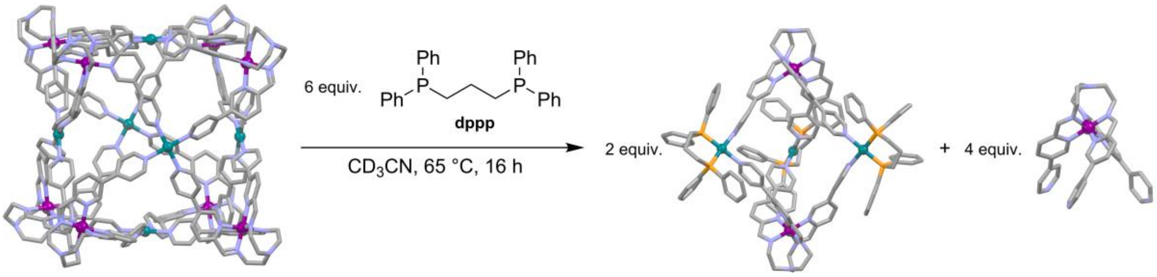

Scheme S9. Complex-to-complex transformation from **CU-2** to **BP-2** and **ML-2**.

ML-2$(BF_4)_2$ (8.00 mg, 9.15 µmol, 8.00 equiv.) together with *tetrakis*(acetonitrile)palladium(II) tetrafluoroborate ($[Pd(CH_3CN)_4](BF_4)_2$; 3.05 mg, 6.86 µmol, 6.00 equiv) in 0.7 mL of acetonitrile-d_3 was degassed by applying a vacuum and flushing with argon three times and heated under an argon atmosphere at 65 °C for 5 days to assemble **CU-2**. After cooling to room temperature 1,3-*bis*(diphenylphosphino)propane (dppp; 2.83 mg, 6.86 µmol, 6.00 equiv) was added. The resulting solution was degassed again three times and heated under an argon atmosphere at 65 °C for 16 hours. Then, a ^{1}H-NMR spectrum was measured to evaluate the reaction progress without any work up. In order to isolate the product mixture, the solution was added to 25 mL of diethyl ether drop wise and was stirred for 30 minutes. Then, the bluish precipitant was collected, washed with generous amounts of diethyl ether and dried in air. The product mixture was obtained as a dark bluish solid 11.3 mg).

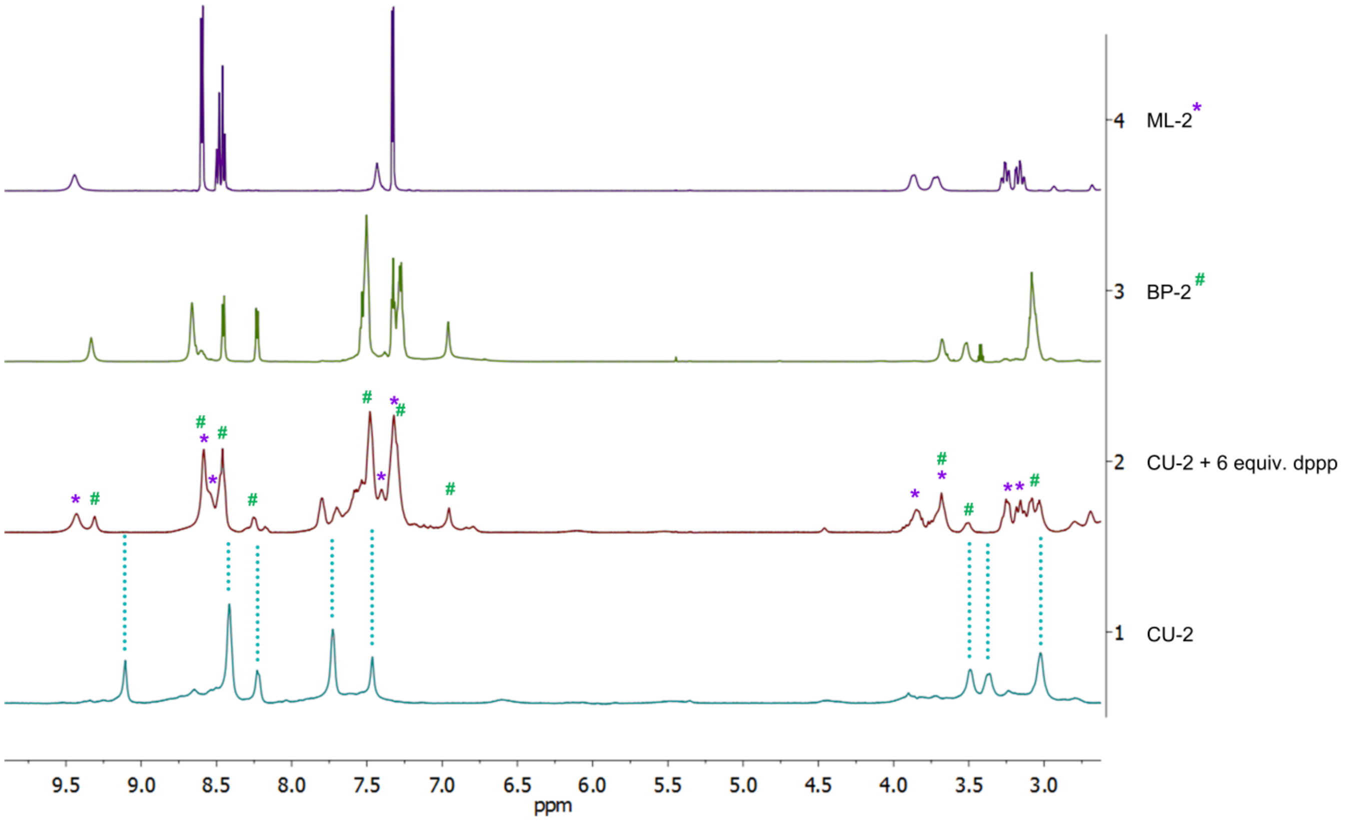

Figure S67. ^{1}H-NMR spectra of: Top (500 MHz, acetonitrile-d_3, 298 K) **ML-2**; Second from top (700 MHz, acetonitrile-d_3, 298 K) **BP-2**; Second from bottom (500 MHz, acetonitrile-d_3, 298 K) **CU-2** + 6 equiv. dppp after 16 hours at 65 °C; Bottom (500 MHz, acetonitrile-d_3, 298 K) **CU-2**. The blue dotted lines are guides to the eye in order to show the absence of signals referring to **CU-2** after the complex-to-complex transformation.

Figure S67 shows that after 16 hours at 65 °C no signals referring to the cube can be observed anymore, proving that **CU-2** was consumed quantitatively in the exchange reaction. Only the superimposed spectra of **BP-2** and **ML-2** were detected, showing the transformation to these complexes. The assembly process of **CU-2** takes 5 days, whereas the formation of **BP-2** is finished after only 16 hours, which shows that the metalloligand might be better preorganized to build up the bipyramidal cage, compared to cubic cages. This might be an additional driving force for this ligand exchange reaction.
We also investigated the temperature dependent behaviour of the reaction mixture containing **ML-2** and **BP-2** (Figures S68 and S69).

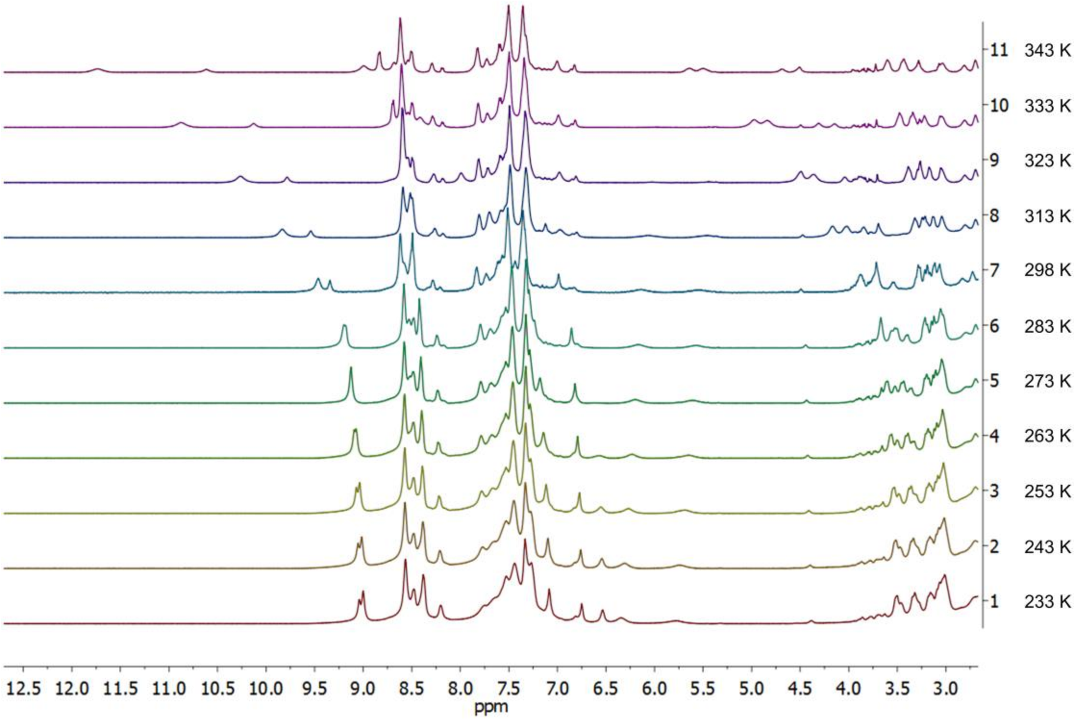

Figure S68. Temperature-dependent ^{1}H-NMR spectra (500 MHz, acetonitrile-d_3) of **CU-2** + 6 equivalents dppp, showing signals of **ML-2** and **BP-2**.

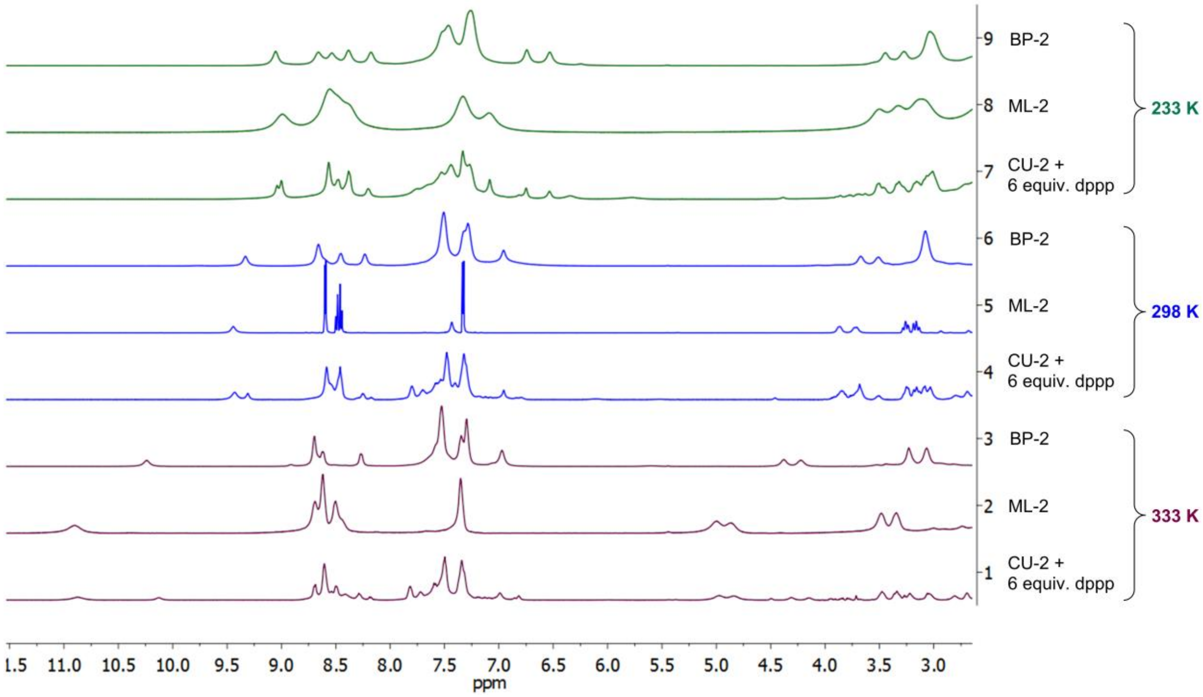

Figure S69. Comparison of ^{1}H-NMR spectra of **ML-2**, **BP-2** and **CU-2** + 6 equivalents dppp (500 MHz, acetonitrile-d_3) at selected temperatures.

Figure S68 shows that upon heating the complex solution containing **BP-2** and **ML-2** the signals experience a significant low-field shift above room temperature. We attribute this behaviour to a beginning spin crossover process, which was already

reported with other electron deficient 2-formylpyridine derivatives.[21] The electron withdrawing 4-pyridyl substituent in this work is comparable to a fluoride substituent in the same position. The reduced electron density of the ligand system facilitates the stabilization of the paramagnetic high-spin state. However, in our case we only observe the very beginning of this spin crossover process with the imine signals shifted to only 12 ppm at 343 K (Figure S70). As observed with the sterically strained complexes in this work one would expect this signal shifted to 250 ppm in case of a purely paramagnetic complex. Therefore the observations with **ML-2** and **BP-2** only show that a minor amount of iron(II) centres switched into the paramagnetic high-spin state at 343 K. Also, figure S69 shows that the mixture of **BP-2** and **ML-2** shows the same behaviour as pure samples of these complexes.

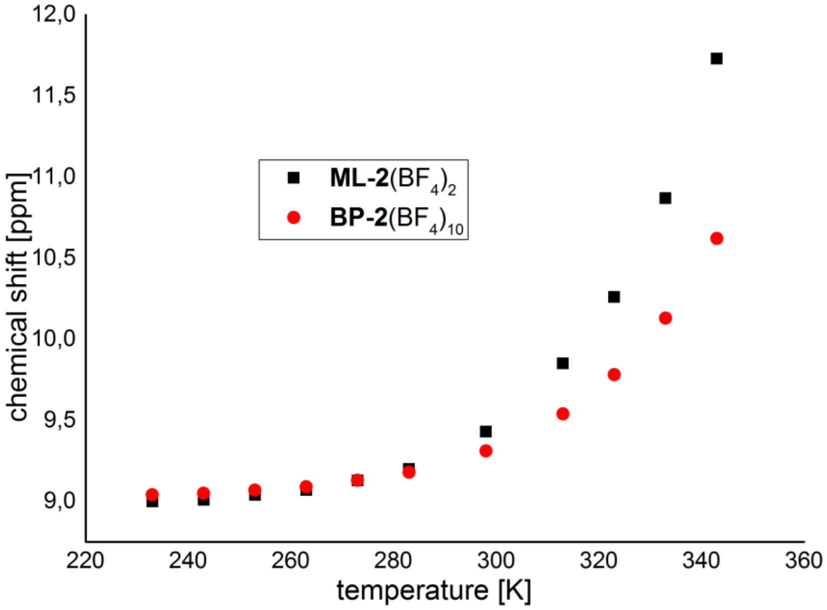

Figure S70. Temperature dependency of chemical shifts of ^{1}H-NMR imine proton signals of **ML-2** and **BP-2**.

[21] N.Struch, F. Topić, G. Schnakenburg, K. Rissanen, A. Lützen, *Inorg. Chem.* **2018**, *57*, 241 – 250.
